# Unravelling homologous recombination repair deficiency and therapeutic opportunities in soft tissue and bone sarcoma

**DOI:** 10.15252/emmm.202216863

**Published:** 2023-02-13

**Authors:** Lara Planas‐Paz, Alicia Pliego‐Mendieta, Catherine Hagedorn, Domingo Aguilera‐Garcia, Martina Haberecker, Fabian Arnold, Marius Herzog, Lorenz Bankel, Roman Guggenberger, Sabrina Steiner, Yanjiang Chen, Abdullah Kahraman, Martin Zoche, Mark A Rubin, Holger Moch, Christian Britschgi, Chantal Pauli

**Affiliations:** ^1^ Laboratory for Systems Pathology and Functional Tumor Pathology, Department of Pathology and Molecular Pathology University Hospital Zurich Zurich Switzerland; ^2^ Molecular Tumor Profiling Laboratory, Department of Pathology and Molecular Pathology University Hospital Zurich Zurich Switzerland; ^3^ Department of Medical Oncology and Haematology University Hospital Zurich Zurich Switzerland; ^4^ Diagnostic and Interventional Radiology University Hospital Zurich Zurich Switzerland; ^5^ Swiss Institute of Bioinformatics Lausanne Switzerland; ^6^ Precision Oncology Laboratory, Department for Biomedical Research Bern Center for Precision Medicine Bern Switzerland; ^7^ Medical Faculty University of Zurich Zurich Switzerland

**Keywords:** genomic instability, HRDness, HRD score, sarcoma, Cancer, Chromatin, Transcription & Genomics, DNA Replication, Recombination & Repair

## Abstract

Defects in homologous recombination repair (HRR) in tumors correlate with poor prognosis and metastases development. Determining HRR deficiency (HRD) is of major clinical relevance as it is associated with therapeutic vulnerabilities and remains poorly investigated in sarcoma. Here, we show that specific sarcoma entities exhibit high levels of genomic instability signatures and molecular alterations in HRR genes, while harboring a complex pattern of chromosomal instability. Furthermore, sarcomas carrying HRD*ness* traits exhibit a distinct SARC‐HRD transcriptional signature that predicts PARP inhibitor sensitivity in patient‐derived sarcoma cells. Concomitantly, HRD^high^ sarcoma cells lack RAD51 nuclear foci formation upon DNA damage, further evidencing defects in HRR. We further identify the WEE1 kinase as a therapeutic vulnerability for sarcomas with HRD*ness* and demonstrate the clinical benefit of combining DNA damaging agents and inhibitors of DNA repair pathways *ex vivo* and in the clinic. In summary, we provide a personalized oncological approach to treat sarcoma patients successfully.

## Graphical abstract

Defects in the homologous recombination repair pathway causing HRD*ness*, often observed in breast, ovarian, prostate and pancreatic cancer, can be detected in distinct sarcoma subtypes at the genomic and transcriptomic levels, and treated with DNA damage and repair‐based therapeutic approaches.

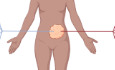

## Introduction

Genomic instability is a hallmark of many cancers. Aberrant proliferation associated with cancer cells promotes the accumulation of genomic alterations and mutations in genes regulating cell division and tumor suppression (Negrini *et al*, 2010; Nguyen *et al*, 2020). Most cells rely on the homologous recombination repair (HRR) pathway for repairing DNA double‐strand breaks (DSB) with high fidelity (Li & Heyer, 2008). Inactivation of genes belonging to the HRR pathway leads to additional genomic alterations, correlates with poor prognosis, and is associated with metastases development (Lord & Ashworth, 2016; Turajlic & Swanton, 2016; Bakhoum *et al*, 2018). The most widespread genetic biomarker of HRR deficiency (HRD) in the clinic is germline or somatic *BRCA1/2* mutation status. However, this approach overlooks partial or complete deletions of the *BRCA1/2* loci or their epigenetic silencing (Farmer *et al*, 2005; Nguyen *et al*, 2020). The prevalence of HRD extends beyond *BRCA1/2* inactivation and includes other genes of the HRR pathway such as *ATM*, *ATR*, *CHK1*, *CHK2*, *PALB2*, *RAD51*, and the *FANC* protein family (McCabe *et al*, 2006). The term BRCAness has been employed to define defects in the HRR pathway without germline *BRCA1/2* mutations, and by name is associated with breast cancer susceptibility (Lord & Ashworth, 2016). In contrast, HRD*ness* encompasses tumor‐agnostic HRR pathway deficiencies and will be used throughout this study. Accurate detection of HRD is of clinical relevance as it is indicative of sensitivity to targeted therapy with poly ADP‐ribose polymerase inhibitors (PARPi) as well as to DNA damaging agents (McCabe *et al*, 2006; Nguyen *et al*, 2020). PARPi trap PARP1/DNA nucleoprotein complexes and stall the advancement of the replication fork. Cancer cells that cannot adequately repair DSB DNA damage via the high‐fidelity HRR pathway alternatively use more error‐prone DNA repair mechanisms. Treatment with PARPi ultimately induces synthetic lethality in cancer cells with HRD, sparing normal tissue. PARPi can further enhance DNA damage and treatment eventually results in chromosomal instability (CIN), which is a form of genomic instability common in most cancers. CIN refers to the high rate of structural and numerical abnormalities in cancer cells compared with normal cells (Negrini *et al*, 2010; Sonnenblick *et al*, 2015).

HRD is frequently observed in ovarian and breast cancer, followed by prostate and pancreatic cancer (Nguyen *et al*, 2020). Recent studies have identified traits of HRD*ness* in soft tissue and bone sarcoma (Kovac *et al*, 2015; Seligson *et al*, 2019; Li *et al*, 2020). Sarcomas are rare mesenchymal cancers, accounting for approximately 1% of all malignancies (Cancer Genome Atlas Research Network, 2017b). Sarcoma comprise a very heterogeneous group of more than 100 distinct histological subtypes originating anywhere in the soft tissue of the body and in bone. To date, there are few treatment options for most sarcoma entities, which often translates into dismal prognosis. Standard of care for soft tissue sarcoma (STS) is usually radiotherapy and resection, while distinct soft tissue and mainly bone sarcoma undergo neo‐adjuvant chemotherapy, surgery and adjuvant chemotherapy (Meyer & Seetharam, 2019). Locally advanced or metastatic STS often show chemo resistance and metastatic disease is related with a poor prognosis. First‐line therapy for advanced disease includes doxorubicin as single agent or in combination with ifosfamide. Median progression‐free survival (PFS) in first‐line therapy for unresectable STS ranges between 4.5 and 6 months. Second‐line treatment is increasingly tailored toward distinct histological subtypes and encompasses the use of chemotherapeutic agents such as trabectedin, eribulin, gemcitabine and taxanes, and the multi‐targeted receptor tyrosine kinase inhibitor pazopanib (Linch *et al*, 2014; Gomez & Tsagozis, 2020). Median PFS for most second‐line therapies remains below 5 months (Meyer & Seetharam, 2019), thus highlighting the unmet medical need to identify new therapeutic venues for STS treatment.

The rarity and the molecular heterogeneity of sarcoma have prevented a detailed study of these mesenchymal cancers, which partially explains the lack of targeted and immunotherapy‐based treatment approaches for the distinct sarcoma entities. Only few sarcomas exist with a relevant alteration that can be targeted, the most common examples being activating *KIT* mutations, *NTRK* or *ALK* fusions. Complex karyotype sarcomas lack well‐understood genomic driver alterations and commonly harbor copy number alterations and loss of tumor suppressor genes such as *TP53*, *RB1*, *NF1*, and *CDKN2A*, which are challenging targets (Chen *et al*, 2020). Our understanding of defects in DNA repair mechanisms in sarcoma remains at an early stage. Recent preclinical studies in bone and STS models have shown enhanced PARPi sensitivity when combined with the DNA damaging agent trabectedin (Ordonez *et al*, 2017; Pignochino *et al*, 2017) or the alkylating agent temozolomide (Oza *et al*, 2020). In addition, PARP1, RAD51, and MCM4 expression have been suggested as promising predictive biomarkers for PARPi sensitivity, HRD phenotype and/or genomic instability (Pignochino *et al*, 2017; Castroviejo‐Bermejo *et al*, 2018; Liu *et al*, 2021).

Here, we broaden the understanding of HRD*ness* in STS and bone sarcoma by performing a comprehensive molecular analysis of HRR deficiency biomarkers and signatures in several independent cohorts of sarcoma using a multi‐omics and cross‐platform approach. We show that distinct sarcoma entities exhibit elevated HRD scores, harbor numerous alterations in common HRR pathway genes, high levels of CIN and are characterized by mutational signatures associated with HRD. Furthermore, HRD^high^ sarcoma entities show a distinct SARC‐HRD transcriptional signature that predicts PARPi sensitivity. We further validate our *in silico* findings using functional data from our own established and molecularly characterized patient‐derived *ex vivo* sarcoma models. We show a lack of RAD51 nuclear foci formation in HRD^high^ sarcoma cells suggesting defective HRR upon DNA damage together with a functional dependency toward PARPi and DNA damaging agents. Both agents in combination exert a synergistic effect in HRD^high^ sarcoma models and show a therapeutic response in a leiomyosarcoma patient, thus evidencing the clinical efficacy and feasibility of this approach. Finally, we show therapeutic benefit of targeting other DNA repair pathways, which advocates for a more general mechanism for treating HRD*ness*.

## Results

### A subset of sarcoma entities exhibit HRD*ness* characteristics

**Figure 1:**
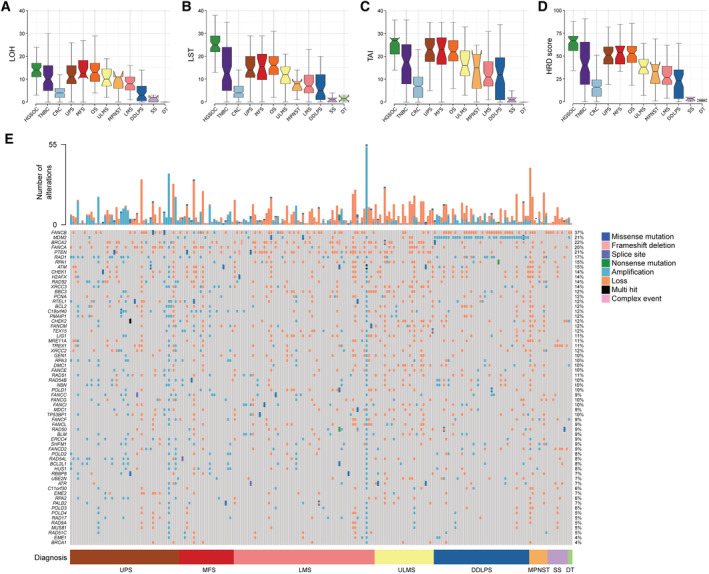
A subset of sarcoma entities exhibit elevated genomic instability signatures and a high degree of alterations in HRR genes Quantification of loss‐of‐heterozygosity (LOH) in soft tissue and bone sarcoma.Quantification of large‐scale transitions (LST) in soft tissue and bone sarcoma.Quantification of telomeric allelic imbalances (TAI) in soft tissue and bone sarcoma.Quantification of homologous recombination deficiency (HRD) score in soft tissue and bone sarcoma.Oncoprint depicting the molecular alterations in 70 HRR genes in soft tissue and bone sarcoma and the total number of alterations. HGSOC, high‐grade serous ovarian cancer; TNBC, triple‐negative breast cancer; CRC, colorectal cancer; UPS, undifferentiated pleomorphic sarcoma; MFS, myxofibrosarcoma; OS, osteosarcoma; ULMS, uterine leiomyosarcoma; LMS, extra‐uterine leiomyosarcoma; DDLPS, dedifferentiated liposarcoma; SS, synovial sarcoma; DT, desmoid tumor. Data information: Datasets from TCGA‐SARC (*n* = 247), TCGA‐OV (*n* = 61), TCGA‐BRCA (*n* = 92), TCGA‐COAD (*n* = 385) and TARGET‐OS (*n* = 69) were used; *n* indicates biological replicates. Data in (A–D) are median ± third and first quartile, the whiskers are minimum and maximum values. Source data are available online for this figure.

Cancers with defects in HRR exhibit characteristic chromosomal changes reflecting the use of alternative and more error‐prone DNA repair pathways (Chopra *et al*, 2020). To investigate whether STS and bone sarcoma subtypes exhibit such HRD*ness* features, we calculated the genomic instability signatures loss of heterozygosity (LOH; Abkevich *et al*, 2012), telomeric allelic imbalance (TAI; Birkbak *et al*, 2012) and large‐scale transitions (LST; Popova *et al*, 2012) in the TCGA‐SARC cohort consisting of 247 cases (Data ref: Cancer Genome Atlas Research Network, 2017a) and the TARGET‐OS cohort of 69 cases (TARGET Osteosarcoma Project, 2009). The HRD score is the sum of all three genomic instability signatures and predicts PARPi sensitivity (Marquard *et al*, 2015; Hodgson *et al*, 2018; Smeby *et al*, 2020). HRD scores for high‐grade serous ovarian cancer (HGSOC), triple‐negative breast cancer (TNBC), and colorectal cancer (CRC) from the TCGA cohorts were previously reported and were used here for comparison (Cancer Genome Atlas Network, 2012a; Data ref: Cancer Genome Atlas Research Network, 2011b, 2012b; Data ref: Cancer Genome Atlas Network, 2012b; Marquard *et al*, 2015; Takaya *et al*, 2020). Our analysis confirmed the reported HRD scores for HGSOC, TNBC, and CRC and showed striking differences in the genomic instability signatures and HRD scores in the different STS and bone sarcoma entities (Fig 1A–D). Undifferentiated pleomorphic sarcoma (UPS), osteosarcoma (OS), myxofibrosarcoma (MFS) and uterine leiomyosarcoma (ULMS) exhibited the highest LOH, LST, and TAI values followed by malignant peripheral nerve sheath tumor (MPNST), extra‐uterine leiomyosarcoma (LMS) and dedifferentiated liposarcoma (DDLPS). While the sarcoma entities with high levels of genomic instability signatures are known to carry complex karyotypes including complex rearrangements in their genomes, the pathogenesis of synovial sarcoma (SS) is associated with a unique chromosomal translocation that results in the expression of the fusion gene *SS18‐SSX*, and mutations in the *CTNNB1* or *APC* genes are the most common cause of desmoid tumors (DT; Cancer Genome Atlas Research Network, 2017b; Kim *et al*, 2018; Knijnenburg *et al*, 2018), most likely explaining the observed differences in the signatures.

We next investigated the somatic mutational landscape of genes that play a role in core reactions of HRR, as well as genes that are associated with the pathway and might confer PARPi sensitivity (Appendix Table S1; Cancer Genome Atlas Research Network, 2011a). Despite the fact that only 4% of sarcoma carry mutations in *BRCA1*, the best characterized HRR pathway gene, we found several other alterations, most in the form of amplifications and losses, in essential and associated members of the HRR pathway, particularly in UPS, MFS, LMS, ULMS, DDLPS and MPNST (Fig 1E). The Fanconi anemia genes *FANCB* and *FANCA* exhibited recurrent amplifications and losses in 37 and 20% sarcoma cases, respectively. In striking contrast to *BRCA1*, *BRCA2* alterations were found in 22% sarcoma. *MDM2* amplifications were found in DDLPS cases and accounted for 21% of all cases. 21% sarcoma cases showed mostly missense mutations and losses of the tumor suppressor *PTEN*, and 17% were characterized predominantly by amplifications of the cell cycle checkpoint gene *RAD1*. Consistent with our previous analysis, SS and DT harbor fewer alterations in the reported HRR genes (Fig 1E).

**Figure EV1:**
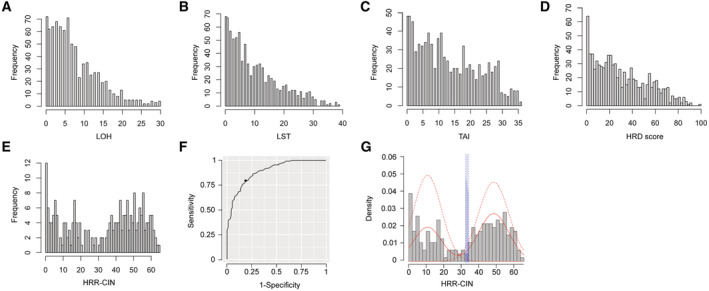
HRD score cut‐off based on genomic alterations in HRR genes (HRR‐CIN) A–DHistograms depicting the frequency of LOH, LST, TAI, and HRD score in sarcoma.EBimodal distribution of HRR‐CIN in sarcoma.FReceiver operating characteristic (ROC) curve of HRD scores from TCGA‐SARC cohort using HRR‐CIN for binary classification.GImplementation of the Youden index on the ROC curve (F) to select the optimal cut‐off value for the HRD score 32 in soft tissue sarcoma. Datasets from TCGA‐SARC (*n* = 247) were used. Source data are available online for this figure.

The total number of alterations in HRR genes per sarcoma in the TCGA‐SARC cohort showed a bimodal distribution, which was not observed for any of the genomic instability signatures (compare Fig EV1E with Fig EV1A–D). Applying a finite mixture model, which is a probabilistic model for representing the presence of subpopulations within an overall population, we classified each sample status as high or low for HRR‐CIN. As the HRD score is one of the most clinically used biomarkers to define HRD*ness*, we plotted a receiver operating characteristic (ROC) curve with HRD score data considering the predicted HRR‐CIN status. We next applied the Youden index to summarize the ROC curve and infer the optimum HRD score cut‐off point as the value with maximum potential effectiveness of a given biomarker. The optimal cut‐off value for the HRD score of soft tissue sarcoma was set at 32. Employing the same method to infer LOH considering the HRD score status, we obtained a cut‐off at 10. The HRD score cut‐off was implemented in the TCGA‐SARC cohort to stratify each STS case (Fig EV1F and G). While 81.3% UPS, 87.5% MFS, 91.3% OS, 70.4% ULMS, 66.7% MPNST, 40.4% DDLPS and 38.4% LMS cases were HRD^high^, all SS or DT were HRD^low^.

**Figure 2:**
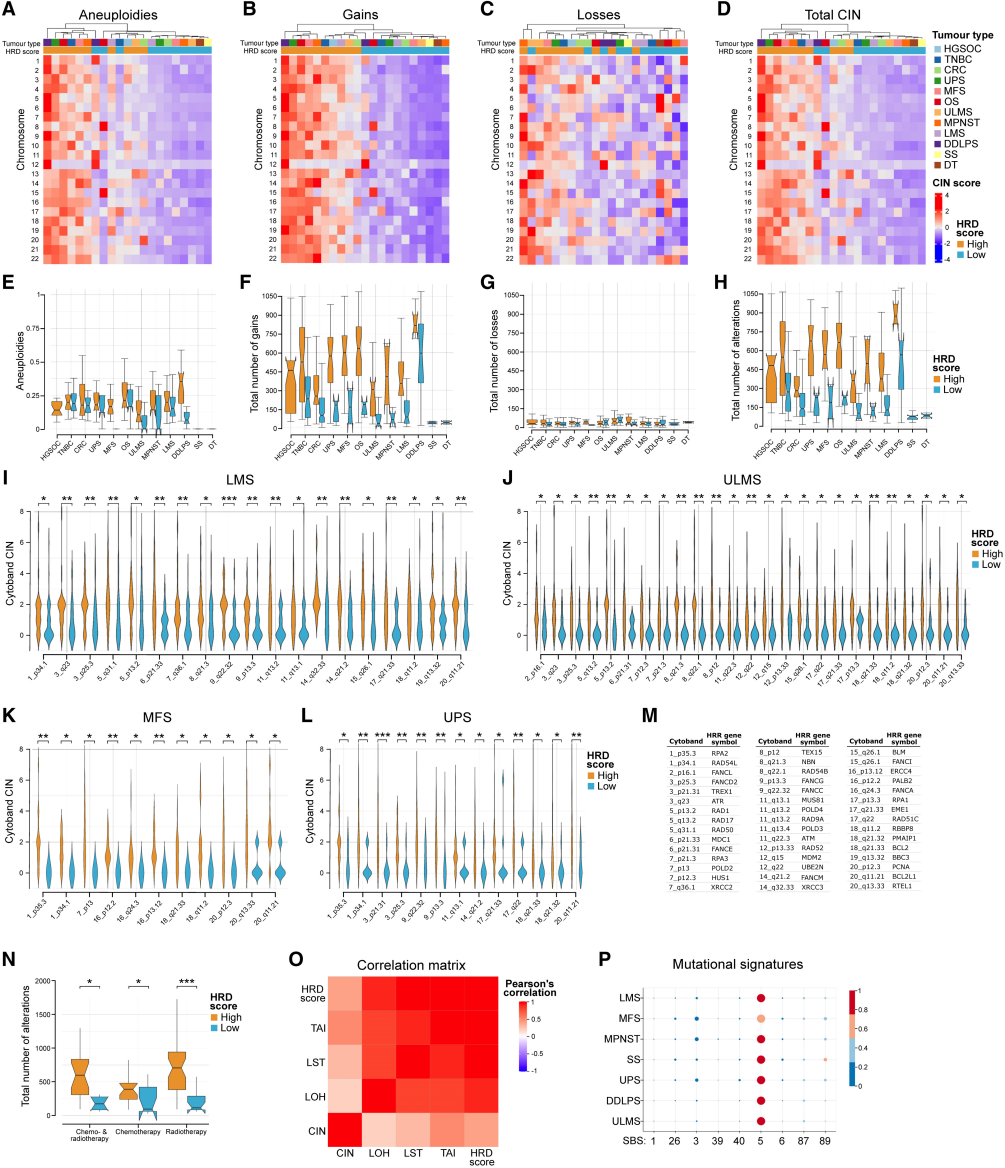
A subset of sarcoma entities exhibit high levels of chromosomal instability and mutational signatures of HRD*ness* A–DHeatmaps with hierarchical clustering showing aneuploidies (A), gains (B), losses (C) and total CIN (D) per chromosome in HRD^high^ and HRD^low^ soft tissue and bone sarcoma, HGSOC, CRC and TNBC.E–HQuantification of aneuploidies (E), gains (F), losses (G) and total number of alterations including gains and losses (H) in HRD^high^ and HRD^low^ soft tissue and bone sarcoma, HGSOC, CRC and TNBC.I–LCIN of cytobands including HRR genes in HRD^high^ LMS (I), ULMS (J), MFS (K) and UPS (L) compared to HRD^low^.MList of the chromosomal cytobands that include HRR genes and exhibit increased CIN in HRD^high^ compared with HRD^low^ sarcoma.NTotal number of alterations in STS patients previously treated with chemotherapy, radiotherapy or both.OCorrelation matrix showing genomic instability signatures and CIN.PMatrix dot plot of mutational signatures in STS. Data information: Datasets from TCGA‐SARC (*n* = 247), TCGA‐OV (*n* = 61), TCGA‐BRCA (*n* = 92), TCGA‐COAD (*n* = 385) and TARGET‐OS (*n* = 69) were used; *n* indicates biological replicates. Data in (A–D) are mean‐centre and scaled, in (E–H) and (N) are median ± third and first quartile, the whiskers are minimum and maximum values. Statistical significance in (I–L) and (N) was determined using Mann–Whitney U‐test and showed statistical differences in (N): Chemo‐ & radiotherapy, *P* = 0.008; Chemotherapy, *P* = 0.047; and Radiotherapy, *P* = 0.0001; *P < 0.05; **P < 0.01; ***P < 0.001. Exact *P*‐values for (I–L) can be found in Appendix Table S3. Source data are available online for this figure.

### A subset of sarcoma entities exhibit high levels of chromosomal instability

Most solid tumors have a form of genomic instability termed CIN that is commonly associated with cancer recurrence and multi‐drug resistance (Negrini *et al*, 2010; Bakhoum *et al*, 2018). CIN refers to the increased rate by which chromosome structure and number changes over time in cancer cells in comparison with normal cells, including gain or loss of whole chromosomes or large chromosomal fragments (Negrini *et al*, 2010). To perform a differential characterization of CIN levels in HRD^high^ and HRD^low^ STS and bone sarcoma cases, we computed several features of CIN in the TCGA‐SARC and TARGET‐OS cohorts (Fig 2). HGSOC and TNBC with *BRCA1* or *BRCA2* loss are characterized by high levels of CIN due to defects in HRR and were used as positive controls for comparison. In contrast, most colorectal cancer (CRC) cases are HR‐proficient (Kuo *et al*, 2009; Data ref: Cancer Genome Atlas Research Network, 2011a, 2012a, 2012b; Data ref: Cancer Genome Atlas Research Network, 2012b; Telli *et al*, 2016; Chen *et al*, 2020) and were also included in the analysis as a negative control. Soft tissue and bone sarcoma cohorts showed high levels of aneuploidies mainly due to chromosomal duplications in HRD^high^ UPS, MFS, OS, ULMS, MPNST, LMS and DDLPS, but not in SS and DT cases (Fig 2A and E). Consistent with the HRD score, the known genomic complexity of the high CIN sarcoma entities compared to the low CIN likely reflects the observed differences.

We next computed the number of gains (Fig 2B and F) and losses (Fig 2C and G) within each chromosome as well as the sum of both (total CIN; Fig 2D and H). While complex karyotype sarcomas were characterized by frequent copy number alterations (CNA) mainly due to high levels of amplifications, fusion‐driven sarcomas such as SS and the mutation‐driven DT displayed considerably fewer CNAs. Higher CIN levels were found in sarcoma entities bearing high HRD scores (Fig 2A–H). Chromosome 12 of DDLPS harbored the highest level of amplifications and CIN (Fig 2B, D, F, and H) irrespective of the HRD score status, confirming previous reports showing highly recurrent focal amplifications at 12q13‐15 known to contain several genes involved in its pathogenesis such as *MDM2* and *CDK4* (Knijnenburg *et al*, 2018). While well‐differentiated LPS (WDLPS) is also characterized by amplification in the same chromosomic region, only the dedifferentiated counterpart acquires more genomic alterations (Coindre *et al*, 2010). When assessing CIN at the level of each chromosome cytoband, we observed significant differences based on the HRD score status. Cytobands in which 52 core or associated genes of the HRR pathway are located, among other genes, showed significantly higher CIN levels in HRD^high^ compared to HRD^low^ sarcoma cases (Fig 2I–M; Appendix Table S2). Higher CIN levels were also identified in patients with high HRD scores after radio‐ and chemotherapy or both combined (Fig 2N). We observed a high correlation among the different genomic instability signatures as well as between the genomic instability signatures and CIN (Fig 2O). Taken together, we identified high levels of aneuploidies, a complex pattern of amplifications and deletions and a high proportion of HRD^high^ cases with multiple alterations in HRR genes across sarcoma entities. Altogether, these results provide a rationale for employing targeted treatments against DNA damage and repair pathways for sarcoma with HRD*ness* traits.

### Mutational signatures suggestive of HRD*ness* in distinct sarcoma entities

To gain better insights into the mutational processes specific to sarcoma, we next investigated the most relevant signatures based on single base substitutions (SBS) across sarcoma entities (Fig 2P). The mutational signature SBS5 showed the highest percentage contribution across STS and was reported to correlate with the age of individuals (Alexandrov *et al*, 2013). SBS3 was the second most prevalent mutational signatures in UPS, MFS and MPNST have been linked with defective HR‐based DNA damage repair, which manifests predominantly as small deletions and insertions and genome rearrangements due to abnormal DSB repair (Nik‐Zainal *et al*, 2012). SBS3 is strongly associated with *BRCA1/2* mutations and correlates with platinum therapy response and therapeutic approaches that exploit HRD. Since tumors with unstable genomes and HRR defects have been shown to respond to agents that further enhance DNA damage, these data also support the implementation of such therapeutic strategies in a personalized manner for sarcoma patients exhibiting HRD*ness* features. In contrast, the fusion‐driven SS exhibited a lower contribution of the SBS3 signature, in line with our previous results that show low CIN and genomic instability signatures as well as few alterations in the HRR pathway in this entity (Figs 1 and 2A–H).

### Additional sarcoma cohorts show a similar pattern of HRD*ness* and chromosomal instability in distinct sarcoma entities

**Figure 3:**
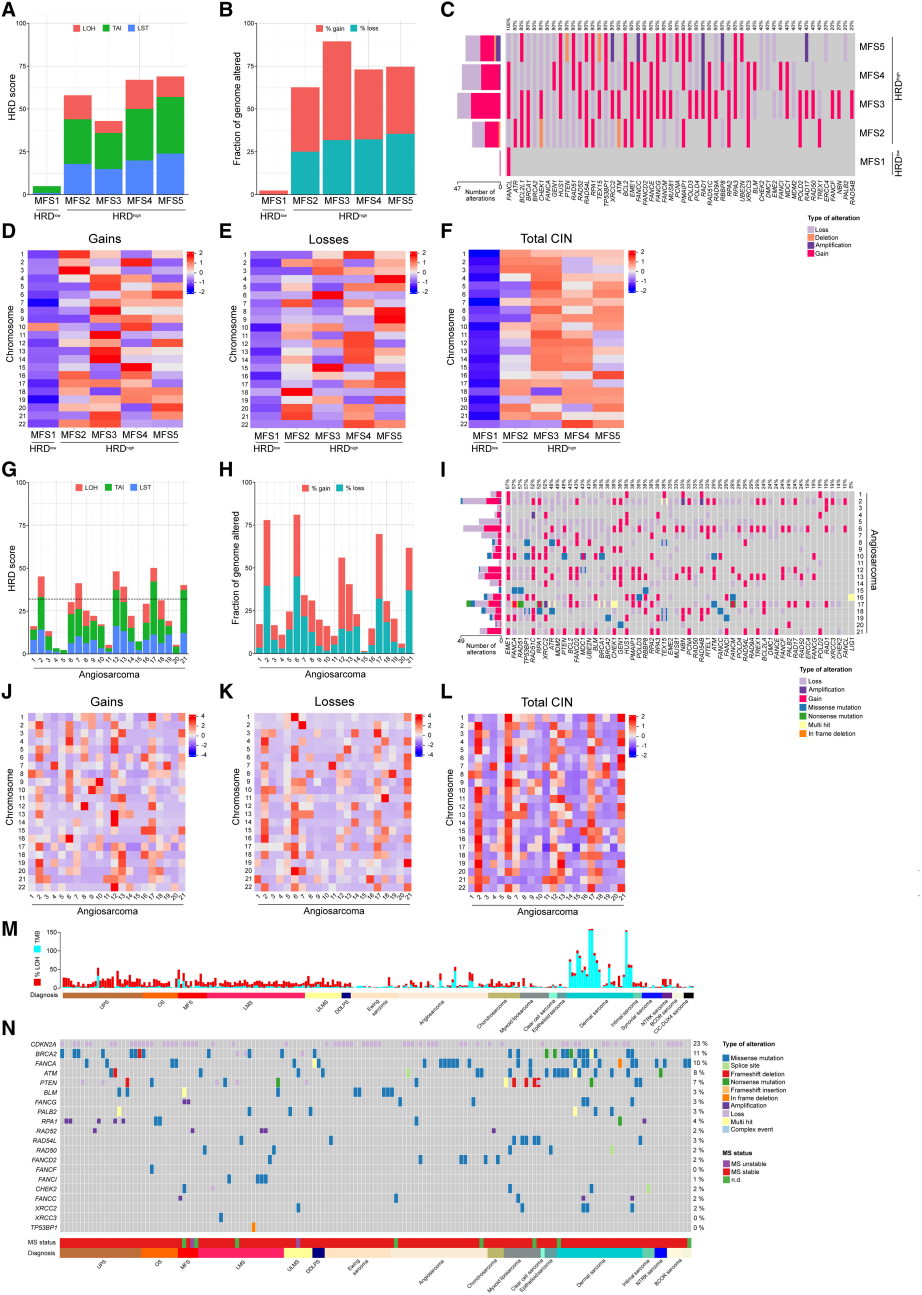
Additional sarcoma cohorts show a similar pattern of HRD*ness* and chromosomal instability in distinct sarcoma entities AQuantification of LOH, LST, TAI and HRD score in MFS.BFraction of genome with gains and losses in an MFS cohort.COncoprint depicting the molecular alterations in HRR genes in MFS and the total number of alterations.D–FHeatmaps of gains (D), losses (E), and total CIN (F) per chromosome in MFS.GQuantification of LOH, LST, TAI and HRD score in angiosarcoma. Dotted line indicates the HRD score cut‐off of 32.HFraction of genome with gains and losses in an angiosarcoma cohort.IOncoprint depicting the molecular alterations in HRR genes in angiosarcoma and the total number of alterations.J–LHeatmaps of gains (J), losses (K) and total CIN (L) per chromosome in angiosarcoma.MLOH and tumor mutational burden (TMB) across several soft tissue and bone sarcoma cohorts. LOH values are reported as genome‐wide percentage of LOH events. TMB values are reported as mutations per megabase.NOncoprint depicting molecular alterations in HRR genes (included in the FoundationOne®HEME assay) in several sarcoma entities as well as their corresponding microsatellite (MS) status. Data information: Datasets from patient cohorts of the University Hospital Zurich were used; *n* = 5 (A–F), 21 (G‐L) and 282 (M, N); *n* indicates biological replicates. Data in (D–F) and (J–L) were mean‐centre and scaled. Source data are available online for this figure.

To further validate our finding that MFS exhibited characteristics of HRD*ness*, we characterized a cohort of five treatment naïve MFS patients by whole‐genome sequencing, including one low‐grade and four high‐grade tumors compared with paired normal samples, and investigated genomic instability signatures and fraction of genome altered within this cohort. The average HRD score for the high‐grade MFS cases (MFS2–MFS5) was 59.3 ± 11.8, and the low‐grade MFS1 presented an HRD score of 5 (Fig 3A). In addition, HRD^high^ MFS2‐MFS5 were characterized by a high degree of their genome exhibiting chromosomal gains and losses (Fig 3B). To gain further insight into the mutational landscape in MFS, we investigated copy number variations among 70 genes involved in HRR regulation (Appendix Table S1). The HRR pathway genes *ATR*, *BCL2L1*, *BRCA1*, *BRCA2*, *CHEK1*, *FANCA*, *FANCL*, *GEN1*, *HUS1*, *PTEN*, *RAD51*, *RAD52*, *RAD54L*, *RPA1*, *TEX15*, *TP53BP1*, and *XRCC2* showed copy number alterations (gains, amplifications, losses, and deletions) in all HRD^high^ MFS. The only altered HRR gene in the HRD^low^ MFS1 was *FANCL* (Fig 3C). We next performed in‐depth analysis of chromosomal instability features and identified a complex pattern of amplifications and deletions in regions of most chromosomes, exclusively in the four cases with higher HRD scores (Fig 3D–F).

**Figure EV2:**
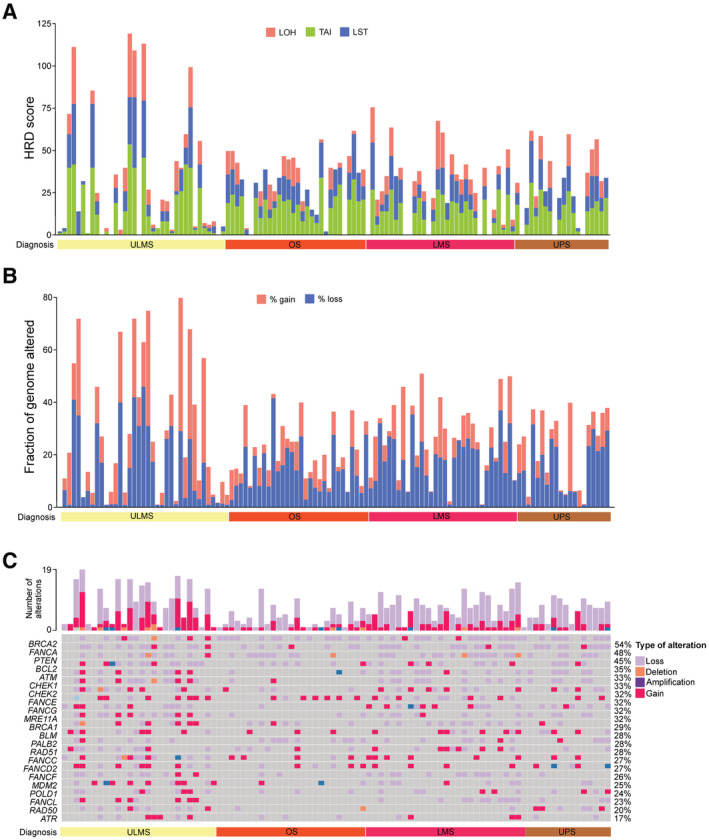
HRD*ness* in soft tissue and bone sarcoma cohorts is associated with molecular alterations in HRR pathway genes LOH, LST, TAI, and HRD score in OS, LMS, ULMS and UPS cohorts.Fraction of genome with gains and losses in OS, LMS, ULMS and UPS cohorts.Oncoprint depicting gains and losses in chromosomal regions of HRR genes (included in the Affymetrix Genome‐wide Human SNP 6.0 arrays and Oncoscan array) and the total number of alterations in OS, LMS, ULMS and UPS cohorts. Data information: Datasets from GSE33153, GSE154591, and GSE119043 were used; *n* = 30 OS, 34 ULMS, 30 LMS, and 20 UPS. Source data are available online for this figure.

We expanded our investigation of genomic instability in STS with an additional cohort of 21 cases of angiosarcoma, a malignancy that affects blood and lymphatic vessels, that were profiled using whole‐genome sequencing. One third of the angiosarcoma cases were HRD^high^ (Fig 3G), were associated with a high percentage of gains and losses in their genome (Fig 3H), and characterized by numerous copy number alterations in HRR genes. *EME1*, *FANCA*, *RAD51*, *TP53BP1*, *RAD51C*, *RPA1*, and *XRCC2* exhibited alterations in more than half the cases of the angiosarcoma cohort (Fig 3I). Elevated levels of gains, losses, and total CIN were observed specially in HRD^high^ angiosarcoma cases (Fig 3J–L). Analysis of available OS, UPS, LMS and ULMS cohorts profiled using genome‐wide microarrays (Data ref: Gultekin *et al*, 2021; Data ref: Kuijjer *et al*, 2012; Data ref: Lesluyes *et al*, 2019) further showed numerous gains and losses of chromosomal regions within HRR genes in HRD^high^ cases as well as an average genomic instability across their genomes of 21% in OS, 22% in UPS, 26% in LMS, and 32% in ULMS (Fig EV2A–C).

We additionally characterized 282 STS and bone sarcoma patients by clinical genomic profiling of a panel of 409 genes, for which all types of genomic alterations in cancer can be detected, including base pair substitutions, insertions, deletions, CNAs and rearrangements. Assessment of the genomic instability signature LOH confirmed UPS, OS, MFS, LMS, ULMS and DDLPS as sarcoma entities with high levels of this biomarker. Furthermore, we identified a subset of angiosarcoma and Ewing sarcoma that also exhibited high LOH values (Fig 3M). Targeted panel sequencing detected mutations and structural alterations in 19 HRR essential and associated genes out of 22 included in the panel. 56.6% of all analyzed sarcoma samples carried alterations in HRR pathway genes; *BRCA2*, *FANCA*, and *ATM* were found among the top altered genes in 11, 10, and 8% of all cases, respectively (Fig 3N). Tumor mutational burden (TMB), defined as the total number of somatic mutations per coding area of a tumor genome, can predict response to checkpoint blockade in certain cancers (Galuppini *et al*, 2019). Typically, high TMB occurs in cancer types developed as a consequence of exposure to carcinogens like tobacco or mutagens such as ultraviolet (UV) light in melanoma (Kang *et al*, 2020). Within our soft tissue and bone sarcoma cohort, dermal sarcoma and a subset of angiosarcoma found in sun‐exposed locations exhibited high TMB, consistent with a UV‐induced mutational pattern (Fig 3M). Taken together, the genomic analysis of several independent cohorts revealed a similar pattern of genomic instability signatures, alterations in HRR genes and CIN features across a broad spectrum of soft tissue and bone sarcoma. Notably, sarcomas with high HRD scores consistently exhibited molecular alterations in genes of the HRR pathway.

### HRD^high^ sarcomas show a distinct SARC‐HRD gene signature and enrichment in DNA repair and cell cycle control pathways

**Figure 4:**
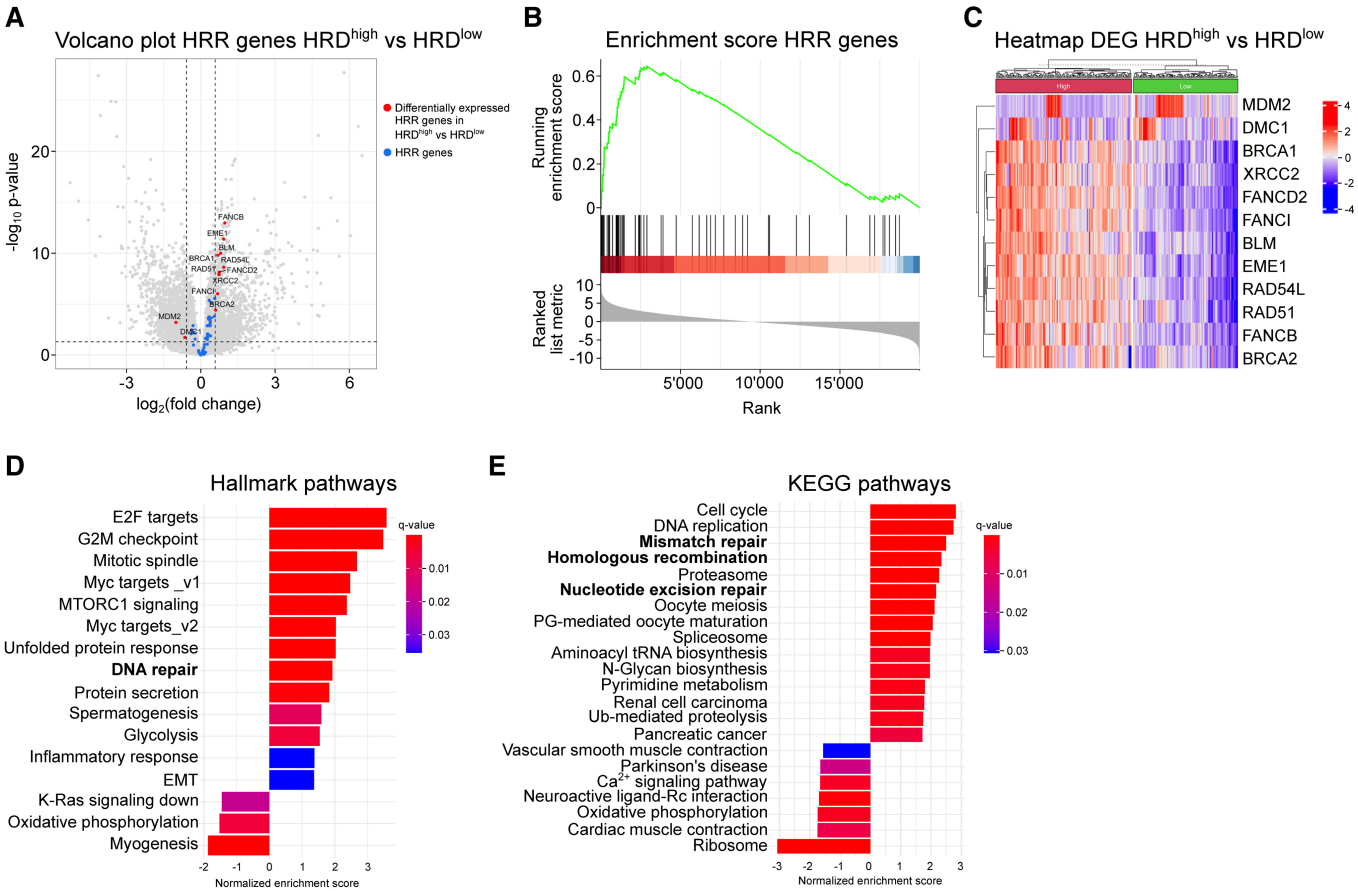
HRD^high^ sarcoma exhibit a distinct SARC‐HRD transcriptional signature and enrichment in DNA repair and cell cycle control pathways AVolcano plot showing enrichment of HRR genes in HRD^high^ compared with HRD^low^ sarcoma cases.BEnrichment score of HRR genes in HRD^high^ compared with HRD^low^ sarcoma cases. Normalized enrichment *P*‐value = 2.5e10^−10^.CHeatmap with hierarchical clustering of differentially expressed genes in HRD^high^ compared with HRD^low^ sarcoma cases.D, EGSEA showing hallmark (D) and KEGG (E) pathways enriched in HRD^high^ compared with HRD^low^ sarcoma cases. Data information: Datasets from TCGA‐SARC (*n* = 247) were used; *n* indicates biological replicates. Source data are available online for this figure.

**Figure EV3:**
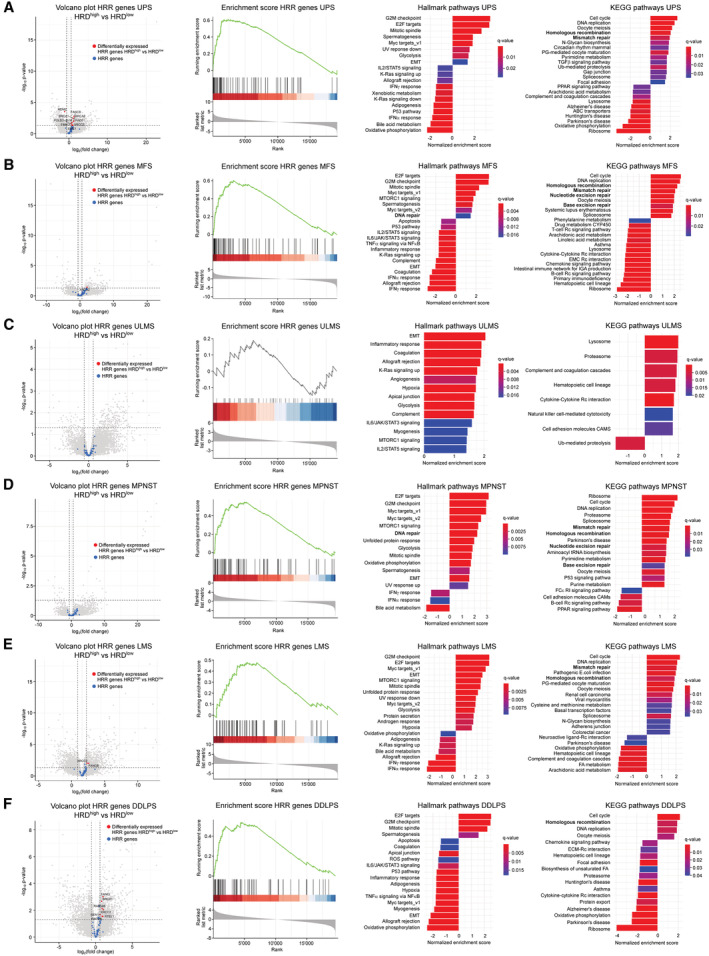
Differential expression of HRR genes and gene set enrichment analysis in HRD^high^ compared with HRD^low^ sarcoma across sarcoma histotypes A–FVolcano plot showing HRR genes, enrichment score of HRR genes, GSEA showing hallmark and KEGG pathways enriched in HRD^high^ compared with HRD^low^ UPS (A), MFS (B), ULMS (C), MPNST (D), LMS (E) and DDLPS (F). Normalized enrichment *P*‐values < 0.01 for UPS, MFS, MPNST, LMS, and DDLPS. Datasets from TCGA‐SARC (*n* = 247) were used. Source data are available online for this figure.

To explore the transcriptional landscape of sarcoma with HRD*ness* traits, we performed a differential gene expression analysis in HRD^high^ cases compared with HRD^low^ of the TCGA‐SARC cohort. Our analysis showed an upregulation of 10 genes in HRD^high^ sarcoma: *BRCA1*, *BRCA2*, *BLM*, *EME1*, *FANCB*, *FANCD2*, *FANCI*, *RAD51*, *RAD54L* and *XRCC2* (Figs 4A and C, and EV3), which we named SARC‐HRD signature, as well as enrichment of genes in the HRR pathway (Fig 4B). *MDM2* and *DMC1* were significantly downregulated in HRD^high^ sarcoma, but showed a sarcoma histotype‐specific expression. While *MDM2* was upregulated in all DDLPS regardless of the HRD status, it appeared significantly downregulated only in UPS (Figs 4A and C, and EV3A). *DMC1* was upregulated in an LMS cluster and unchanged in all other sarcoma entities (Figs 4C and EV3). We next performed a gene set enrichment analysis (GSEA) in HRD^high^ cases compared with HRD^low^ cases, which showed an enrichment in DNA repair pathways including the HRR pathway, among others, across all sarcoma entities except for ULMS (Fig 4D and E, and compare Fig EV3A, B, and D–F with Fig EV3C). In addition, most HRD^high^ sarcoma entities exhibited enriched gene sets associated with cell cycle‐related targets of MYC and E2F transcription factors as well as gene sets involved in the G2/M checkpoint and mTORC1 signaling, some of which were also found to be upregulated in HGSOC (Sohn *et al*, 2021). In contrast, we found the p53 pathway and interferon immune responses among the common downregulated gene sets (Fig EV3). Altogether, we showed an upregulation of 10 HRR genes creating a specific SARC‐HRD gene signature, which was further corroborated at the pathway level. Interestingly, the upregulation of the HRR pathway was accompanied by a general enrichment of DNA damage repair pathways, such as mismatch repair and nucleotide excision repair in HRD^high^ sarcoma (Fig 4E).

### Genomic and transcriptomic characterization of patient‐derived *ex vivo* sarcoma cell models

**Figure 5:**
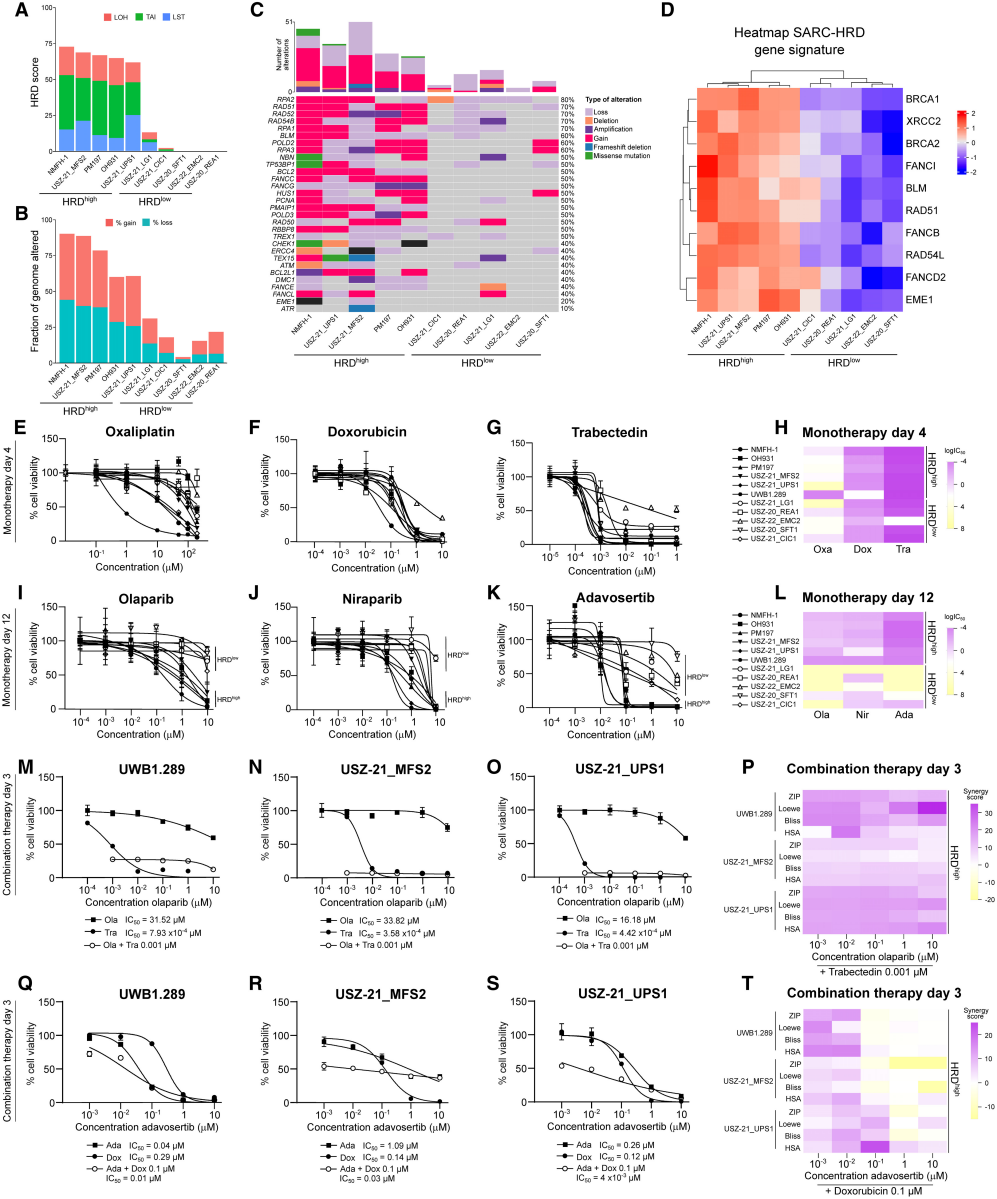
HRD^high^ sarcomas show sensitivity to PARP and WEE1 inhibition and synergy with chemotherapy drugs AQuantification of LOH, LST, TAI and HRD score in patient‐derived sarcoma cell models.BFraction of genome with gains and losses in patient‐derived sarcoma cell models.COncoprint depicting the molecular alterations in HRR genes in patient‐derived sarcoma cell models and the total number of alterations.DHeatmap with hierarchical clustering of the SARC‐HRD gene signature in HRD^high^ compared with HRD^low^ sarcoma cell models. Data was mean‐centre and scaled.E–G
*Ex vivo* treatment of HRD^high^ (NMFH‐1, OH931, PM197, USZ‐21_MFS2 and USZ‐21_UPS1) sarcoma models compared with HRD^low^ (USZ‐20_REA1, USZ‐21_LG1, USZ‐22_EMC2, USZ‐20_SFT1 and USZ‐21_CIC1) sarcoma models for 4 days with six doses of the chemotherapy agents oxaliplatin, doxorubicin, and trabectedin.HHeatmap of IC_50_ showing drug sensitivity responses in all cell models.I, JHRD^high^ sarcoma cell models show sensitivity to the PARPi olaparib (I) and niraparib (J) when treated for 12 days.KHRD^high^ sarcoma cell models show sensitivity to the WEE1 inhibitor adavosertib when treated for 12 days.LHeatmap of IC_50_ showing sensitivity to PARPi and WEE1i in HRD^high^ but not HRD^low^ sarcoma cell models. The ovarian carcinoma cell line UWB1.289 with *BRCA1* mutations was used as positive control for PARPi response and HRD*ness*.M–OHRD^high^ sarcoma cell models and UWB1.289 treated for 3 days with five doses of olaparib alone and in combination with 1 nM trabectedin. Note that no sensitivity to olaparib in monotherapy was observed at 3 days but from 8 days on (see Figs EV5A and B).PHeatmap of the synergy scores ZIP, Loewe, Bliss and HSA showing synergy in the combinatorial modality.Q–SHRD^high^ sarcoma cell models and UWB1.289 treated for 3 days with five doses adavosertib alone and in combination with 100 nM doxorubicin.THeatmap of the synergy scores ZIP, Loewe, Bliss and HSA showing synergy in the combinatorial modality. Data information: *n* = 5 HRD^high^ and 5 HRD^low^ sarcoma cell models (A–L), 2–3 repetitions in technical triplicates (A–S); *n* indicates biological replicates. Data in (E–G), (I–K), (M–O) and (Q–S) are mean ± s.d. Source data are available online for this figure.

**Figure EV4:**
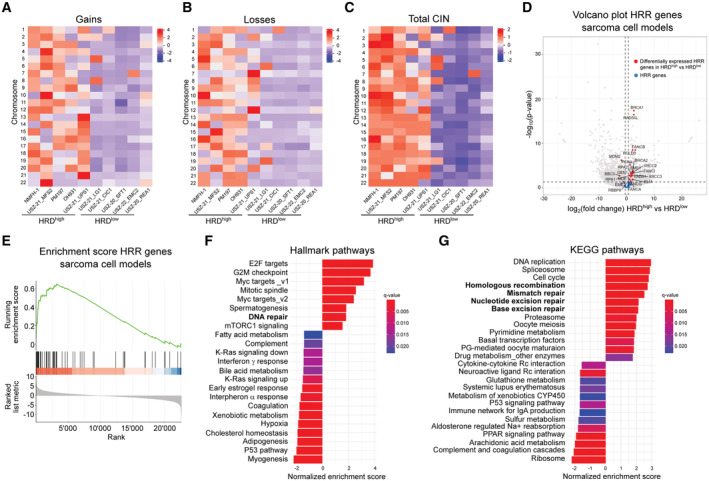
Molecular characterization of patient‐derived sarcoma cell models A–CHeatmaps of gains (A), losses (B) and total CIN (C) per chromosome in sarcoma cell models.DVolcano plot showing enrichment of HRR genes in HRD^high^ compared with HRD^low^ sarcoma cell models.EEnrichment score of HRR genes in HRD^high^ compared with HRD^low^ sarcoma cell models. Normalized enrichment *P*‐value < 0.01.F, GGSEA showing hallmark (F) and KEGG (G) pathways enriched in HRD^high^ compared with HRD^low^ sarcoma cell models. Data information: *n* = 5 HRD^high^ and 5 HRD^low^ sarcoma cell models. Data in (A–C) are mean‐centre and scaled. Source data are available online for this figure.

To investigate novel therapeutic strategies for sarcoma with HRD*ness* traits, we first established genomically and transcriptomically characterized patient‐derived *ex vivo* sarcoma cell models. All cell models faithfully recapitulated the molecular alterations from their corresponding tumor of origin (Fig 5A and B; Bangerter *et al*, 2022; Chen *et al*, 2022; Pauli *et al*, 2022). We molecularly characterized four human MFS cell models, namely NMFH1, OH931, PM197, USZ‐21_MFS2, and one UPS cell model (USZ‐21_UPS1) by whole‐genome sequencing. NMFH‐1 showed loss of *TP53*; OH931, PM197 and USZ‐21_MFS2 exhibited loss of *CDKN2A* and *CDKN2B*. In addition, USZ‐21_MFS2 carried mutations in *ATR*, *MDM2* and *TP53*, and loss of *MLH1*. USZ‐21_UPS1 showed loss of *ATRX* and *RB1*. Genomic profiling also revealed high levels of genomic instability signatures (HRD scores ranging from 62 to 73), a high degree of genomic gains and losses and multiple molecular alterations in HRR pathway genes (Figs 5A–C and EV4A–C). We also established five HRD^low^ models, a *BCOR*‐rearranged sarcoma (USZ‐20_REA1), an unclassified low‐grade sarcoma (USZ‐21_LG1), a fusion‐driven CIC‐DUX sarcoma (USZ‐21_CIC1), a solitary fibrous tumor (USZ‐20_SFT1) with an NAB2‐STAT6 fusion and an extraskeletal myxoid chondrosarcoma (USZ‐22_EMC2) harboring a TAF15‐NR4A3 fusion. The HRD scores for these sarcoma cell models scores were 0, 13, 4, 0, and 2, respectively (Fig 5A). The fraction of altered genome and total number of molecular alterations in HRR genes were lower in HRD^low^ models when compared with HRD^high^ (Fig 5B and C). In addition, we performed differential gene expression analysis comparing the transcriptome of the five genomically characterized patient‐derived HRD^high^ MFS and UPS cell models with five HRD^low^ sarcoma cell models. Our analysis identified an upregulation of the SARC‐HRD gene signature, enrichment for HRR genes and DNA repair pathways in HRD^high^ sarcoma cells (Figs 5D and EV4D–G). The expression of all genes belonging to the SARC‐HRD signature was significantly upregulated and the expression of *MDM2* significantly decreased in patient‐derived HRD^high^ sarcoma cells compared with HRD^low^ (Figs 5D and EV4D), thus validating the SARC‐HRD gene signature in an independent sarcoma cohort.

### HRD^high^ sarcomas show sensitivity to PARP inhibition

**Figure EV5:**
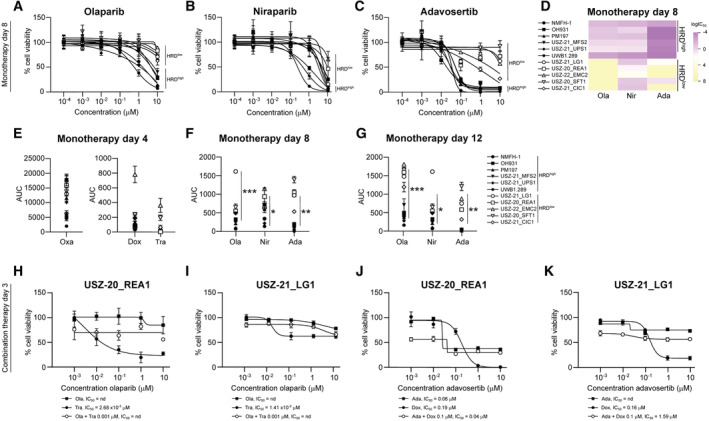
PARPi and WEE1i monotherapy and combination therapy in patient‐derived sarcoma cell models A, BHRD^high^ sarcoma cell models show sensitivity to the PARPi olaparib (A) and niraparib (B) when treated for 8 days.CHRD^high^ sarcoma cell models show sensitivity to the WEE1 inhibitor adavosertib when treated for 8 days.DHeatmap of IC_50_ showing sensitivity to PARPi and WEE1i in HRD^high^ but not HRD^low^ sarcoma cell models. The ovarian carcinoma cell line UWB1.289 with *BRCA1* mutations was used as positive control for PARPi response and HRD*ness*.EArea under the curve (AUC) for oxaliplatin, doxorubicin and trabectedin upon 4 days treatment; corresponding dose–response curves depicted in Fig 5E–G.FAUC for olaparib, niraparib and adavosertib upon 8 days treatment; corresponding dose–response curves depicted in Fig EV5A–C.GAUC for olaparib, niraparib and adavosertib upon 12 days treatment; corresponding dose–response curves depicted in Fig 5I–K.H, IHRD^low^ sarcoma cell models treated for 3 days with five doses olaparib alone and in combination with 1 nM trabectedin.J, KHRD^low^ sarcoma cell models treated for 3 days with five doses adavosertib alone and in combination with 100 nM doxorubicin. Data information: *n* = 5 HRD^high^ and 5 HRD^low^ sarcoma cell models (A–G), *n* indicates biological replicates. Data are mean ± s.d. One‐tailed unpaired *t*‐test comparing AUC of HRD^high^ and HRD^low^ sarcoma cell models showed significant differences in olaparib, niraparib and adavosertib response in HRD^high^ and HRD^low^ cell models; *P < 0.05; **P < 0.01; ***P < 0.0001; ns, not significant (*P* > 0.05); *P* = 2.75 × 10^−5^ (Ola, F), *P* = 0.039 (Nir, F), *P* = 1.49 × 10^−4^ (Ada, F), *P* = 1.17 × 10^−5^ (Ola, G), *P* = 0.028 (Nir, G), *P* = 6.82 × 10^−4^ (Ada, G). Source data are available online for this figure.

PARP1 and PARP2 play critical roles in single‐strand DNA break repair and in maintenance of genomic stability mainly through the base excision repair (BER) pathway. PARPi block the BER pathway and, in cells with HRD, cells rely on the error‐prone non‐homologous end joining (NHEJ) pathway for DNA repair (McCabe *et al*, 2006). As a result, cells accumulate massive genetic damage, which ultimately leads to cell death. *In vitro* sensitivity to DNA double‐strand break‐inducing drugs, such as platinum salts, is also a feature of HRD cells. We have identified sarcoma entities with HRD*ness* characteristics, thus supporting the use of PARPi and platinum salts for treating sarcoma patients with an HRD^high^ status. We subjected 10 patient‐derived *ex vivo* sarcoma cell models to treatment with two PARPi, olaparib and niraparib, as well as to the standard chemotherapy agents doxorubicin, an anthracycline antibiotic that blocks topoisomerase 2, and the alkylating agents oxaliplatin and trabectedin (Fig 5E–L). To evaluate cellular responses to PARP inhibition, we treated cells for 8 and 12 days with six doses of PARPi and performed ATP measurements (CellTiter‐Glo) as a surrogate for metabolically active and thus viable cells. We employed the ovarian cancer cell line UWB1.289 that presents a pathogenic frameshift mutation in *BRCA1* and an HRD score of 67 as a control to evaluate the drug response of the HRD^high^ sarcoma models. Our five patient‐derived HRD^high^ MFS and UPS cell models show sensitivity to both PARPi, with IC_50_ in a comparable range as the IC_50_ in *BRCA1*‐mutated ovarian carcinoma cells. In contrast, HRD^low^ sarcoma models exhibited significantly lower sensitivity to olaparib and niraparib treatment compared with the HRD^high^ models (Figs 5I, J, and L, and EV5A, B, D, F, and G). This data shows that MFS and UPS cell models characterized by high HRD scores are susceptible to PARP inhibition in a dose–response manner. We found no major differences between HRD^high^ and HRD^low^ sarcoma models in their response to oxaliplatin, doxorubicin, and trabectedin in monotherapy (Figs 5E–H and EV5E). We next assessed whether a combinatorial modality of trabectedin and olaparib resulted in synergism in HRD^high^ sarcoma. After 3 days combinatorial drug therapy, we observed a synergistic effect of the combined drugs in HRD^high^ sarcoma cells as well as in *BRCA1*‐mutated ovarian carcinoma cells, but not in two HRD^low^ sarcoma cells (compare Fig 5M–O with Fig EV5H and I). We employed SynergyFinder to compute the four drug synergy scores zero interaction potency (ZIP), Loewe, Bliss and highest single agent (HSA), which evidenced the synergistic effect between olaparib and trabectedin (Fig 5P). These data highlight the therapeutic potential of trabectedin and olaparib combination in sarcoma with HRD*ness*, which is currently explored in clinical trials (NCT04076579).

### Inhibition of WEE1 is a new therapeutic strategy for HRD^high^ sarcoma

We hypothesized that our patient‐derived UPS and MFS cell models might show a generalized susceptibility to other agents targeting DNA damage and repair pathways. To identify additional drug susceptibilities in HRD^high^ sarcoma based on their genomic instability traits, we focused on the WEE1 protein, which belongs to the serine/threonine family of protein kinases. WEE1 acts by inhibiting CDK1 and CDK2 and thus promotes temporary cell cycle arrest and DNA damage repair. WEE1 exhibits a high number of CNA in sarcoma and its inhibition has been linked to increased genomic instability (Dominguez‐Kelly *et al*, 2011; Martin *et al*, 2011). In ovarian and endometrial cancers, WEE1 inhibition renders *TP53*‐deficient cells sensitive to radiation and DNA‐damaging agents such as PARPi and chemotherapeutic agents (Meng *et al*, 2018). To investigate the effect of WEE1 inhibition in HRD^high^ sarcoma, we performed a six‐point dose–response curve with the WEE1 inhibitor adavosertib in our *ex vivo* patient‐derived sarcoma models. HRD^high^, but not HRD^low^, sarcoma cells showed adavosertib sensitivity with IC_50_ at the nanomolar range after 8 and 12 days treatment (Figs 5K and L, and EV5C, D, F, and G). The response of the HRD^high^ sarcoma models to WEE1 inhibition was comparable with the *BRCA1*‐mutated ovarian carcinoma cells. We next assessed whether doxorubicin increased the sensitivity to adavosertib in HRD^high^ sarcoma cells. We observed a synergistic effect of adavosertib together with 0.1 μM doxorubicin in HRD^high^ UPS and MFS but not in HRD^low^ sarcoma (compare Fig 5Q–T with Fig EV5J and K). Altogether, these data show that targeting DNA damage response and DNA repair pathways are powerful therapeutic strategies for sarcoma with HRD*ness* traits.

### Absence of RAD51 nuclear foci in patient‐derived HRD^high^ sarcoma cells upon trabectedin and olaparib‐induced damage

**Figure 6:**
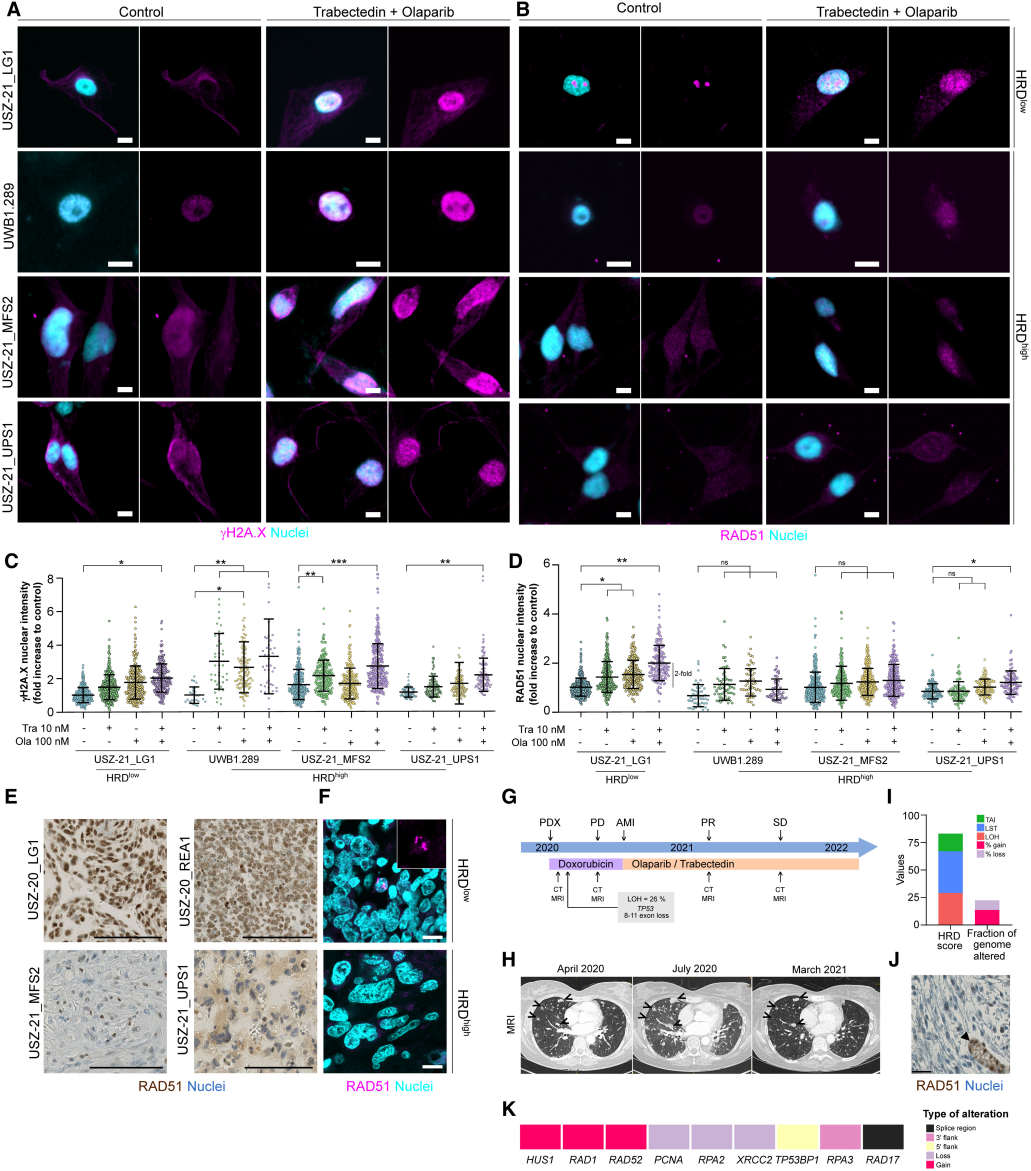
Absence of RAD51 nuclear foci in patient‐derived HRD^high^ sarcoma cells upon trabectedin and olaparib‐induced DNA damage AImmunofluorescence showing nuclear expression of the DNA damage marker γH2A.X (magenta) upon 6 h treatment with 10 nM trabectedin and 100 nM olaparib in combination in HRD^high^ and HRD^low^ sarcoma cell models as well as ovarian carcinoma UWB1.289 cells.BImmunofluorescence showing RAD51 nuclear foci (magenta) upon 6 h treatment with 10 nM trabectedin and 100 nM olaparib in combination only in the HRD^low^ sarcoma cell model (USZ‐21_LG1).C, DQuantification of γH2A.X (C) or RAD51 (D) nuclear intensity per cell compared to untreated control cells.EImmunohistochemistry (IHC) in human tissue sections showing RAD51 (brown) expression in HRD^low^ but not HRD^high^ sarcoma patients. Small‐nucleated lymphocytes and/or proliferating cells appeared RAD51‐positive in HRD^high^ models.FImmunofluorescence in human tissue sections showing RAD51 (magenta) nuclear foci in an HRD^low^ but not an HRD^high^ sarcoma patient.GTimeline of LMS patient diagnosis and treatment.HMagnetic resonance imaging (MRI) of LMS patient since primary diagnosis. Open arrowheads point at metastatic lesions. PDX, primary diagnosis; PD, progressive disease; AMI, acute myocardial infarction; PR, partial response; SD, stable disease; CT, computer tomography; MRI, magnetic resonance imaging.IGenomic profiling of a gastric metastasis showing HRD score and fraction of genome altered.JRAD51 IHC in the metastatic patient's tissue. Compare RAD51 nuclear expression in normal tissue (arrowhead points at gastric gland) but not in tumorous gastric tissue.KOncoprint depicting the molecular alterations in HRR genes in the metastatic sample. Data information: *n* = 2–3 biological replicates in technical triplicates (A–D), *n* indicates biological replicates. Scale bars, 100 μm (E), 25 μm (J), 10 μm (A, B, F). Data are mean ± s.d. A one‐way ANOVA with Sidak's multiple comparison test revealed statistically significant differences in (C) (*F*(3, 8) = 3, *P* = 0.09 for USZ‐21_LG1; *F*(3, 8) = 12.6, *P* = 0.002 for UWB1.289; *F*(3, 8) = 52.9, *P* < 0.0001 for USZ‐21_MFS2; *F*(3, 8) = 7.4, *P* = 0.01 for USZ‐21_UPS1) and (D) (*F*(3, 8) = 10.4, *P* = 0.004 for USZ‐21_LG1; *F*(3, 8) = 2.2, *P* = 0.17 for UWB1.289; *F*(3, 8) = 1.3, *P* = 0.34 for USZ‐21_MFS2; *F*(3, 8) = 37.4, *P* < 0.0001 for USZ‐21_UPS1). *P < 0.05; **P < 0.01; ***P < 0.0001; ns, not significant (*P* > 0.05). Source data are available online for this figure.

To functionally assess HRR *ex vivo*, we investigated biomarkers of HRR in our patient‐derived sarcoma models and their corresponding originating tumor tissue (Fig 6). The DNA repair protein RAD51 is a central player in the core mechanism of HRR, catalyzing the recognition of homology and strand exchange between homologous DNA partners to join a processed DNA break and the repair template. The presence of RAD51 nuclear foci is considered a functional biomarker of HRR and can be predictive of PARPi resistance (Li & Heyer, 2008; Castroviejo‐Bermejo *et al*, 2018; Cruz *et al*, 2018). We evaluated the formation of RAD51 nuclear foci using immunofluorescence in *ex vivo* MFS and UPS sarcoma cell models upon trabectedin and olaparib‐induced DNA damage. We visualized the extent of DNA damage by staining for the phosphorylated histone variant H2A.X forming γH2A.X, which marks DNA DSB (Mah *et al*, 2010). High levels of γH2A.X are also associated with high sensitivity to agents that further enhance DNA damage (Huang & Zhou, 2020). 10 nM trabectedin and 100 nM olaparib in monotherapy increased γH2A.X nuclear expression, and the combination regimen further enhanced it in all our tested cell models (Fig 6A and C; Appendix Fig S1A). In contrast, whereas we observed a 2‐fold increase in RAD51 nuclear intensity as well as formation of nuclear foci in the HRD^low^ USZ‐21_LG1, neither formation of RAD51 nuclear foci nor increased RAD51 nuclear intensity were observed in the MFS and ovarian carcinoma cell models (Fig 6B and D; Appendix Fig S1B). The slight increase (0.4‐fold) in RAD51 nuclear expression in UPS cells treated with the combinatorial modality did not translate into formation of nuclear foci (Fig 6B and D; Appendix Fig S1B), which is a key step for its function (Haaf *et al*, 1999). Moreover, RAD51 expression and nuclear foci were reduced in HRD^high^ UPS and MFS tissue compared with both HRD^low^ sarcoma patient samples (Fig 6E and F). Our combined results demonstrate defective HRR in sarcoma cell models with increased genomic instability levels and PARPi sensitivity.

### Trabectedin and olaparib combination treatment show clinical benefit in a leiomyosarcoma patient

A 47‐year‐old female patient presented with multiple lesions in lungs and liver in April 2020, but the location of the primary tumor was unknown. Pathological review confirmed metastatic LMS, most likely of uterine origin. The patient immediately underwent a systemic therapy with pegylated liposomal doxorubicin (PLD). Due to the widespread metastatic disease, broad molecular profiling was performed, which evidenced a loss of exons 8–11 and a rearrangement in intron 7 within the *TP53* gene. In addition, targeted panel sequencing also revealed a stable microsatellite status, TMB of 7 Mut/Mb and an LOH score of 26%, which was considered high in the hospital‐based molecular tumor board (Fig 6G). Treatment with PLD had to be terminated because the patient suffered from a myocardial infarction and underwent cardiopulmonary resuscitation, due to an anthracycline‐induced cardiomyopathy. From October 2020, the patient was treated off‐label with a combination of trabectedin and olaparib and remained in a lasting radiological partial remission for one and a half years (Fig 6G and H). Genomic profiling of a stomach metastasis in late 2022 revealed an HRD score of 83 and 22% of structural genomic alterations (Fig 6I). Genomic gains in the HRR genes *HUS1*, *RAD1* and *RAD52* as well as losses in *PCNA*, *RPA2* and *XRCC2*, and absence of nuclear RAD51 expression in the metastatic tissue further supported a defect in HRR mechanisms (Fig 6J and K). This case highlights the potential benefits of extending the therapeutic indications for these drugs alone or in combination. Altogether, our data provide the groundwork for clinical testing of DNA damaging agents and DNA repair targeting drugs in new sarcoma indications.

## Discussion

To date, very few targeted options exist to successfully treat sarcoma, thus highlighting the need to investigate specific disease mechanisms for the distinct sarcoma entities to guide development of new personalized therapies. Here, we performed a comprehensive and systematic analysis of genomic instability biomarkers in sarcoma and cross‐validated our results using multiple independent sarcoma cohorts. We identified high levels of CIN and other genomic instability signatures in sarcoma with complex karyotypes. Determining the genomic instability scars LOH, LST and TAI as well as the HRD score has proven clinically relevant as it can predict sensitivity to PARPi, the gold‐standard approach to treat HRD tumors. Commercial clinical tests such as the MyriadMyChoice®CDx assay and the FoundationOne®CDX assay are widely used to detect HRD at the genomic level. The MyriadMyChoice®CDx assay uses an HRD cut‐off value of 42 to predict response to platinum‐containing neoadjuvant chemotherapy in patients with TNBC (Telli *et al*, 2016) and recurrent ovarian cancers treated with niraparib (Mirza *et al*, 2016; Gonzalez‐Martin *et al*, 2019). The FoundationOne®CDX assay uses a cut‐off value of LOH > 16% to predict response to PARPi in ovarian cancers (Coleman *et al*, 2017). Currently, no clinically validated diagnostic test exists for determining HRD*ness* in soft tissue or bone sarcoma and the designated threshold for the different genomic signatures in ovarian and breast cancer is not necessarily applicable to other tumor entities, as has been reported for pancreatic cancer (Zhuang *et al*, 2021). By assessing the CIN levels in HRR genes, the only signature that showed a bimodal distribution pattern, we could infer an optimal cut‐off value for the HRD score in STS applying the Youden index. We defined an HRD score higher or equal to 32 to classify the HRD status of each sarcoma case. This cut‐off correlates with high PARPi sensitivity in patient‐derived *ex vivo* sarcoma models. An independent study defined a cut‐off of 35 based on sarcoma patient survival curves (Li *et al*, 2020). Nevertheless, a clinical trial addressing PARPi sensitivity is required for validating the clinical utility of this biomarker for soft tissue and bone sarcoma.

An alternative mechanism of HRD*ness* was reported in Ewing sarcoma. Interestingly, this fusion‐driven bone sarcoma lacks genomic instability but shows PARPi sensitivity (Gorthi & Bishop, 2018). The proposed underlying mechanism is an exacerbated transcription that is considered to cause a widespread accumulation of R‐loops, which in turn prevents BRCA1 relocation to sites of damage and HRR (Gorthi *et al*, 2018). Moreover, complex chromosomal rearrangements resembling chromothripsis were observed in LMS, OS and LPS (Chudasama *et al*, 2018; Cortes‐Ciriano *et al*, 2020). Chromothripsis is another form of genomic instability observed across many tumor types in particular those with *TP53* loss. It is characterized by a catastrophic chromosomal event that causes clustered genomic rearrangements in a few cell divisions (Maciejowski *et al*, 2015; Cortes‐Ciriano *et al*, 2020).

Our mutational analysis evidenced that most STS patients carry alterations in genes involved in the HRR pathway and mutational signatures based on single‐base substitutions common in HR‐deficient cancer. *BRCA1/2* mutations are well‐known causes of HRD, 4 and 22% of sarcoma patients carry mutations in *BRCA1* and *BRCA2*, respectively. Notably, reversion mutations in *BRCA2* mediating PARPi resistance have been reported in ovarian, breast, pancreatic and prostate cancer, highlighting the benefit of implementing molecular profiling to pre‐emptively detect them in clinical settings. Moreover, two members of the Fanconi anemia core complex, *FANCA* and *FANCB*, the *TP53* regulator *MDM2*, the tumor suppressor *PTEN*, cell cycle checkpoint regulators *RAD1* and *CHEK1*, DNA damage and replication proteins *ATM*, *RPA1* and *H2AFX* completed the list of the 10 top altered HRR genes in the TCGA‐SARC cohort. Our genomic analyses are in line with recently published work (Gounder *et al*, 2022; Nacev *et al*, 2022) that described molecular patterns and genomic instability signatures in distinct sarcoma histotypes using a large cohort of sarcoma patients. In contrast to their findings, we identified a significant number of gene alterations in an extended HRR gene set, thus contributing to elucidate potential molecular mechanisms for HRD*ness* in distinct sarcoma histotypes. Although molecular alterations in HRR genes besides *BRCA1/2* are less established surrogate markers for HRD, mutations in *ATM* and *PALB2* but not in other HRR genes were shown to be associated with sensitivity to PARPi in prostate carcinoma in a phase 3 clinical trial (Hussain *et al*, 2020). In addition, not only *BRCA1/2*‐mutant patients but also *BRCA1/2* wild type exhibiting high LOH were shown to benefit from PARPi therapy (Coleman *et al*, 2017). How individual genetic aberrations or the overall changes within the HRR molecular landscape in sarcoma contribute to creating HRD*ness* or predict platinum and PARPi sensitivity is yet to be shown.

In addition to our comprehensive genomic analyses, we also identified a distinct SARC‐HRD gene expression signature that was able to predict PARPi and WEE1i sensitivity in *ex vivo* sarcoma cell models. The SARC‐HRD gene signature includes 10 HRR genes whose expression was significantly increased across all HRD^high^ sarcoma cases from the TCGA‐SARC cohort. The activation of the HRR pathway might be due to HR‐deficient cells trying to compensate at the transcriptional level for the dysfunction in the pathway. Similarly, upregulation of HRR genes was previously reported for osteosarcoma (Barenboim *et al*, 2021). We could validate a significant increase in the 10 genes of the SARC‐HRD signature in our patient‐derived sarcoma models. Thus, the SARC‐HRD signature shows promise and warrants further corroboration and clinical validation. In contrast to the other soft tissue sarcoma entities, the transcriptomic profiling analysis of HRD^high^ ULMS showed an enrichment neither in HRR nor in other DNA repair pathways. ULMS has been shown to separate from other sarcomas into a distinct molecular cluster (Cancer Genome Atlas Research Network, 2017b), likely explaining the observed differences. Interferon responses were commonly downregulated in most HRD^high^ sarcoma histotypes. Notably, PARPi treatment was shown to induce the innate immune interferon response via the cGAS‐STING pathway (Chopra *et al*, 2020; Kim *et al*, 2020; Bruand *et al*, 2021), which underlines a potential additional mechanism of action of this drug.

RAD51 is a key player for restarting stalled replication forks via HRR, thus reducing DNA damage and protecting cells from apoptosis. We demonstrated a functional impairment in HRR in patient‐derived sarcoma cell models harboring multiple alterations in HRR genes and high HRD scores. HRD^high^ UPS and MFS cells lacked RAD51 nuclear expression and foci formation upon DNA damage elicited with a combinatorial modality of PARPi and chemotherapy. In addition, we showed that HRD^high^ UPS and MFS patient tissue have reduced RAD51 nuclear expression compared with HRD^low^ low grade and a BCOR‐rearranged sarcoma patient tissue. Formation of RAD51 nuclear foci was proposed as a predictive biomarker for platinum and PARPi response in pre‐clinical studies (Castroviejo‐Bermejo *et al*, 2018; Pellegrino *et al*, 2022). Our work also supports the use of RAD51 as a potential and easily affordable biomarker for HRD*ness*, particularly in diagnostic settings where NGS is not readily available. Interestingly, RAD51 overexpression contributes to genomic instability and increased RAD51 levels in *BRCA*‐deficient cells are a described mechanism of resistance to DNA damaging treatment (Richardson *et al*, 2004; Hannay *et al*, 2007; Liu *et al*, 2017). Additional candidate biomarkers whose clinical utility warrants further clinical validation are PARP1 (Pignochino *et al*, 2017), MCM4 (Liu *et al*, 2021) and high levels of pH2AX and MAP17 (Perez *et al*, 2020).

By using patient‐derived *ex vivo* sarcoma cell models, we showed that PARPi was effective against HR‐deficient sarcoma cells that lacked *BRCA* mutations but exhibited (i) elevated levels of CIN in core and associated genes of the HRR pathway, and (ii) high genomic instability scores. Our HRD^high^ sarcoma cell models also showed high sensitivity to WEE1 inhibition, a tyrosine kinase involved in G_2_ checkpoint signaling. Either combination of the PARPi olaparib with the alkylating agent trabectedin or the WEE1i adavosertib with the anti‐tumor antibiotic doxorubicin elicited a high degree of drug synergy in HRD^high^ sarcoma cells, demonstrating the efficacy of two different therapeutic approaches for treating HR‐deficient sarcoma cells. The efficacy of both olaparib and trabectedin combination therapy as well as adavosertib in combination with chemotherapeutic agents are currently under clinical investigation in either metastatic or advanced sarcoma (NCT04076579), recurrent ovarian cancer (NCT02101775) or relapsed or refractory solid tumors in pediatric patients (NCT02095132). We have already demonstrated that an off‐label use of such drug combinations is beneficial in an LMS patient, providing additional support for such clinical trials.

Interestingly, we observed high levels of CIN and high HRD scores in patients that had previously undergone radio‐, chemotherapy or both, which can exacerbate genomic instability (Hendry, 2001). Whether PARPi is a viable therapeutic option for treatment‐acquired HRD*ness* is yet to be clinically proven. Measuring the degree of genomic instability in a treatment naïve tumor may prove relevant to guide personalized treatment. Beyond PARP inhibition, other targeted agents have been explored in HR‐deficient tumors, such as against topoisomerase II and c‐Abl (Siddiqui *et al*, 2021), PLK1 and CHEK (Yoshida *et al*, 2022), mTOR alone or in combination with DNA repair inhibitors (Mo *et al*, 2016; El Botty *et al*, 2018), among others.

In summary, we provide a comprehensive genomic, transcriptomic and functional investigation of HRD*ness* in sarcoma. The use of an HRR‐CIN score or the newly identified SARC‐HRD gene expression signature might enhance the identification of patients that benefit from DNA damage and DNA repair‐based therapies and therefore warrants further investigation to personalize treatment for sarcoma patients. Future research could benefit from exploring specific mechanisms of PARPi resistance in sarcoma and focus on the identification of treatment combination regimens to prevent and overcome development of resistance.

## Materials and Methods

### Data collection

Data license was acquired for TCGA data portal (https://portal.gdc.cancer.gov) and VCF files, allele‐specific copy number, gene‐level copy number and RNA‐sequencing raw counts were downloaded. A total of 247 soft tissue sarcoma including 54 dedifferentiated liposarcoma (DDLPS), 2 desmoid tumors (DT), 73 extra‐uterine leiomyosarcoma (LMS), 24 myxofibrosarcoma (MFS), 9 malignant peripheral nerve sheath tumors (MPNST), 10 synovial sarcoma (SS), 27 uterine leiomyosarcoma (ULMS) and 48 undifferentiated pleomorphic sarcoma (UPS) were analyzed (Data ref: Cancer Genome Atlas Research Network (2017a)). In addition, 61 high‐grade ovarian adenocarcinoma and 92 triple‐negative breast cancer with *BRCA1* or *BRCA2* mutations, and 385 colorectal carcinoma from TCGA (Data ref: Cancer Genome Atlas Research Network, 2011b; Data ref: Cancer Genome Atlas Network, 2012b; Data ref: Cancer Genome Atlas Research Network, 2012b), as well as 69 osteosarcoma (OS) cases from the TARGET project (TARGET Osteosarcoma Project (2009)) were included in the analysis.

Targeted next‐generation sequencing data obtained with the FDA‐approved, broad molecular diagnostic test FoundationOne®HEME assay (Foundation Medicine Inc.) for the following cases: 41 angiosarcoma, 5 *BCOR*‐rearranged sarcoma, 14 chondrosarcoma, 4 *CIC‐DUX4* sarcoma, 3 clear cell sarcoma, 4 DDLPS, 36 dermal sarcoma, 4 epithelioid sarcoma, 20 Ewing sarcoma, 4 intimal sarcoma, 46 LMS, 12 MFS, 13 myxoid liposarcoma, 4 *NTRK*‐fused sarcoma, 12 OS, 9 SS, 16 ULMS and 35 UPS. The assay sequences DNA of the complete exons of 409 cancer‐related genes for the detection of short variants (single nucleotide variants, insertions and deletions), copy number alterations as well as microsatellite status and tumor mutational burden (TMB), selected introns and promotor regions of 31 genes involved in rearrangements, in addition to RNA sequencing of 265 genes. Differently to LOH scores reported by scarHRD (Sztupinszki *et al*, 2018), LOH scores determined by the FoundationOne®HEME assay are measured as genome‐wide percentage of LOH events. Tumor tissue DNA was profiled without paired normal control DNA, which may lead to unintentional inclusion of germline variants. Short nucleotide polymorphism (SNP) analysis detects sample purity and possible contaminations, thus allowing for detection and exclusion of any cross‐contaminated samples.

An OS cohort and a mixed cohort of UPS and LMS profiled using Affymetrix Genome‐wide Human SNP 6.0 arrays were downloaded from the Gene Expression Omnibus database repository with accession numbers GSE33153 and GSE154591, respectively (Data ref: Kuijjer *et al*, 2012; Data ref: Lesluyes *et al*, 2019). In addition, the ULMS cohort with accession number GSE119043 profiled using the Oncoscan array was also downloaded (Data ref: Gultekin *et al*, 2021). After filtering low‐quality samples, 30 OS, 34 ULMS, 30 LMS and 20 UPS were analyzed.

### Whole‐genome sequencing (WGS)

DNA was extracted with the Maxwell® 16 DNA/RNA Purification Kits (Promega) from fresh frozen native tumor tissue or FFPE together with matched normal tissue. DNA quantification was performed via Nanodrop (ThermoFisher Scientific) and Qubit ds DNA BR Assay Kit (ThermoFisher Scientific) or Qubit 3.0 Fluorometer (ThermoFisher Scientific). 200 ng of dsDNA was fragmented into ~ 200 bp fragments by sonication (Covaris system) prior to purification (AMPure XP Beads; Agencourt). Library construction was performed using NEBNext kits (NEB, E6040S). DNA extracted from four high‐grade and one low‐grade MFS tissues together with matched normal samples were sequenced using the Illumina HiSeq X instrument as described by the manufacturer. Primary tumor tissue derived from UPS, MFS, *BCOR*‐rearranged sarcoma, unclassified low‐grade sarcoma, fusion‐driven CIC‐DUX sarcoma, solitary fibrous tumor and extraskeletal myxoid chondrosarcoma patients, their respective patient‐derived cell models (USZ‐21_UPS1, USZ‐21_MFS2, USZ‐20_REA1, USZ‐21_LG1, USZ‐21_CIC1, USZ‐20_SFT1 and USZ‐22_EMC2), as well as an angiosarcoma cohort consisting of 21 cases together with matched normal samples were sequenced using Illumina NovaSeq 6000 instrument following the manufacturer's instructions. Three MFS cell models (NMFH‐1, OH931 and PM197) and ovarian carcinoma cells (UWB1.289) were also subjected to whole‐genome sequencing for genomic instability signature and copy‐number variation analysis. Cells, tumor and normal sample sequencing was performed with 60 or 30‐fold coverage depth and analyzed with the Dragen Bio‐IT platform v3.9.5 (Illumina).

### Segmentation and allele‐specific copy number (ASCN) calling

Raw data (.CEL) from SNP arrays (Oncoscan and SNP6.0) were processed using the R package EaCoN (https://github.com/gustaveroussy/EaCoN). BAF and LRR data were used as input in ASCAT copy number R package (https://github.com/cancerit/ascatNgs), which infers a sample's ASCN profiles. Gamma values were selected for each sample based on the goodness of fit curve plots. BAM files from tumor and normal pairs were input in ascatNgs to generate allele‐specific copy number data from WGS. The reference SNP positions for both array and WGS data were recommended by ASCAT.

### Genomic instability signatures LOH, LST, TAI and HRD score

HRD score was computed using the R package scarHRD (Sztupinszki *et al*, 2018). This score is the unweighted, linear sum of genetic alterations embracing loss‐of‐heterozygosity (LOH), large‐scale transitions (LST) and telomeric allelic imbalance (TAI). LOH implies to the loss of genomic regions larger than 15 Mb without covering whole chromosomes. LST includes chromosomal breaks between adjacent regions of at least 10 Mb, with an inter breakage distance no larger than 3 Mb. TAI is the number of unequal contributions of allele sequences in the telomeric regions of chromosomes.

### Genomic instability

Chromosomal instability (CIN) from TCGA data was computed on the allele specific copy number segments by adding the number of gains (CNV > 2) and the number of losses (CNV < 2) per sample and chromosome. The same threshold method was applied at the level of specific cytobands in order to compute their genomic instability.

For array and WGS data, gains and losses were computed overall on the segments based on the L2R values. Segments with a positive L2R greater than 0.1 were considered as gains, whilst negative segments less than −0.1 were considered as losses. To identify high level amplifications and homozygous deletions, copy number variations (CNV) with log2R > 0.7 (high level gain) and < −0.7 (deep deletions) were implemented as used by convention in cBioPortal (Cerami *et al*, 2012). Total genomic instability was computed as the fraction of the whole genome that presented gains and losses. The same was implemented at the chromosome level, where the genomic instability was computed as the fraction of every chromosome that presented genomic aberrations of gains or losses.

Chromosomal aneuploidy events were analyzed using the CNV for each segment. Events with a loss or gain greater than 90% found within a chromosome were considered as aneuploidy.

### HRR‐CIN score and HRD score cut‐off

The HRR‐CIN score was calculated from the copy number profiles by computing the CIN specifically in genes belonging to the HRR pathway. A finite mixture model to assess bimodal data was used and thereafter a cut‐off value that separates the two peaks in the bimodal distribution was implemented as described in the R package cut‐off (https://github.com/choisy/cutoff). The confidence interval from the cut‐off was computed by Monte Carlo simulations and resulted in an HRR‐CIN cut‐off value of 37. Sample population was stratified into HRR‐CIN^high^ and HRR‐CIN^low^. In order to infer a cut‐off for the HRD score, we plotted a receiver operating characteristic (ROC) curve with HRD score data. An ROC curve illustrates the diagnostic ability of a binary classifier as the classification thresholds are varied. ROC curves show the trade‐off between sensitivity (x axis: true positive rate) and specificity (y axis: false positive rate, 1‐specificity). While a random classifier gives curves along the diagonal, classifiers that fives curves closer to the top‐left corner indicate better performance. Subsequently, the Youden index was applied using the R package cutpointr as it summarizes the ROC curve and shows the maximum potential effectiveness of a biomarker, which in this case resulted in an optimal value of 32 for the HRD score. The same method applied to LOH data from the TCGA‐SARC cohort and resulted in an optimal cut‐off value of 10 for LOH.

### Oncoprints and mutational signatures

Oncoprints including genes from the HRR pathway (Appendix Table S1; Cancer Genome Atlas Research Network, 2011a) were generated using maftools (Mayakonda *et al*, 2018) from MAF (mutation annotation format) files downloaded from TCGA. Additionally, copy number amplifications and copy number deletions were computed as described above and added to the oncoprints. For the analysis of WGS, VCF files were filtered to include somatic variants and variants with a quality equal to PASS. Filtered VCF files were annotated by VEP (https://github.com/Ensembl/ensembl‐vep) and converted to MAF using vcf2maf tool (https://github.com/mskcc/vcf2maf). From samples sequenced with the FoundationOne^®^HEME assay, somatic mutations of known significance, reported amplifications and deletions as well as reported tumor mutational burden and percentage LOH were included. Of note, not all HRR pathway genes are covered in this assay.

Mutational signatures were computed using the R package MutationalPatterns (Manders *et al*, 2022).

### RNA‐sequencing

RNA from patient‐derived sarcoma cell models was extracted using the Maxwell RSC simplyRNA Tissue kit (Promega AS1340). The extracted RNA was quantified with a Nanodrop (ThermoFisher Scientific) and the High Sensitivity RNA ScreenTape Assay for TapeStation systems (Agilent 5067). RNA‐sequencing libraries were prepared using the Illumina Stranded mRNA sample preparation protocol following the manufacturer's instructions. Each library was sequenced in paired‐end mode using the NovaSeq 6000 platform and resulting reads were mapped to the human transcriptome.

Differential gene expression between HRD^high^ and HRD^low^ sarcoma samples from the TCGA cohort was investigated by downstream processing of raw counts using the R package DESeq2 (Love *et al*, 2014). In‐house RNA‐sequencing data were processed using the R package tximportData to import the quantification files generated by *Salmon* into *DESeq2*. Genes with a two‐fold change and a *P*‐value equal to or smaller than 0.05 were considered differentially expressed.

Gene set enrichment analysis (GSEA) was used to determine significantly different gene sets between HRD^high^ and HRD^low^ samples. ‘h.all.v7.5.1.symbols’ and ‘c2.cp.kegg.v7.5.1.symbols’ gene sets were downloaded from the Molecular Signatures Database (MSigDB database; Liberzon *et al*, 2015) for GSEA analysis. The ClusterProfiler R package (Yu *et al*, 2012) was used for KEGG pathway and MSigDB Hallmark pathway enrichment analysis. Adjusted *P*‐value < 0.01 was considered statistically significant.

### Patient‐derived sarcoma cell models

Patient‐derived sarcoma cell models were authenticated and established as previously described (Bangerter *et al*, 2022; Chen *et al*, 2022) from both male and female patients to avoid gender bias. Informed consent was obtained from all subjects. The present study was conducted following regional/cantonal and institutional guidelines and in compliance with the WMA Declaration of Helsinki and after approval by our cantonal ethical review board Zurich (BASEC‐2021‐00417).

### Cell culture

Patient‐derived sarcoma cells were genomically characterized by the FoundationOne®HEME assay and Infinium Human Methylation EPIC 850 k array (Illumina) targeted gene sequencing panel and maintained in DMEM (Gibco) with 10% fetal bovine serum (Gibco). Ovarian carcinoma cells UWB1.289 (ATCC, CRL‐2945, RRID:CVCL_B079) were genomically characterized by the FoundationOne^®^CDX assay targeted gene sequencing panel, which confirmed a pathogenic frameshift mutation in *BRCA*1 (2475delC). Cells were maintained in 1:1 RPMI‐1640 medium (Gibco) and Mammary Epithelial Cell Growth Medium (MEGM Bullet kit, Lonza, CC‐3150). All cells were passaged when sub‐confluent conditions were reached in cell culture‐treated flasks, incubated at 37°C in a humidified atmosphere with 5% CO_2_ and routinely tested for *Mycoplasma*.

### Functional testing

Cells were detached from the cell culture flasks by adding TrypLE Express (ThermoFisher, 12604) followed by a brief incubation at 37°C in a humidified atmosphere with 5% CO_2_. Medium‐containing serum was added, cells were collected and centrifuged for 3 min at 500 *g* at RT. Cell pellets were reconstituted in medium and cells were counted using the Countess automated cell counter (ThermoFisher Scientific). Depending on the assay duration, a total of 500–1,000 cells/well were seeded onto collagen‐coated 96‐well plates (Corning, 354407). The following chemotherapeutic and targeted agents were added to the cells 24 h later spanning 6 dose levels: doxorubicin (Selleckchem, S1208), oxaliplatin (Selleckchem, S1224), trabectedin (Selleckchem, S7758), olaparib (Selleckchem, S1060), niraparib (Selleckchem, S2741), adavosertib (Selleckchem, S1525) and cells were subsequently incubated at 37°C in a humidified atmosphere with 5% CO_2_. For short‐term treatment, cell viability was measured 4 days later with CellTiter‐Glo 2.0 (Promega, G9241) following the manufacturer's instructions. For longer treatments, cells were retreated every 4 days and cell viability was measured eight and 12 days after the first treatment. All experiments were performed in triplicates and repeated. Luminescence readout was performed on white 96‐well LumiNunc plates (VWR, 732‐2698) for 1,000 ms using the Multimode Plate Reader Infinite 200 Pro (Tecan). All data were normalized to untreated control, a non‐linear regression was used to calculate IC_50_ and area under the curve in GraphPad Prism (GraphPad Software, Inc). SynergyFinder web application was used to investigate synergistic drug effects (Zheng *et al*, 2022).

### Immunofluorescence and immunohistochemistry

Cells were seeded into μ‐slide eight‐well chamber slides (Ibidi, 80806) or black 96‐well plates with clear glass bottom (ThermoFisher, 165035). On the next day, cells were treated with 10 nM trabectedin and 100 nM olaparib as single agents or in combination for 6 h at 37°C in a humidified atmosphere with 5% CO_2_. Following three washes in 1x PBS, cells were fixed in 4% paraformaldehyde or cold methanol for 20 min. at RT and washed again three times in 1x PBS. Fixed cells were permeabilized and blocked in 0.2% Triton X‐100 (Sigma‐Aldrich), 3% bovine serum albumin (BSA; Sigma‐Aldrich, A3912) for 1 h at RT under gentle agitation. Immunofluorescence was performed with the following antibodies: rabbit anti‐RAD51 (Abcam, ab133534 [EPR4030], RRID:AB_2722613) diluted 1/250 or rabbit anti‐γH2A.X (phospho S139; Abcam, ab81299, RRID:AB_1640564) diluted 1/250 in blocking/permeabilization solution o/n at 4°C under gentle agitation. Following three washes in 1x PBS, cells were incubated with donkey anti‐rabbit secondary antibodies conjugated to Alexa488 (Jackson ImmunoResearch, 711‐545‐152) for 45 min at RT under gentle agitation. Finally, cells were washed three times in 1x PBS and stored at 4°C. Imaging was performed in the ImageXpress Pico imaging system (Molecular Devices) using the same settings for each experiment. Brightness and contrast were adjusted equally to all images in each experiment using Fiji. A macro was implemented to automatically identify cell nuclei and measure nuclear RAD51 and γH2A.X intensity in each cell. Nuclear staining intensity per cell was normalized to untreated control and fold increase of nuclear RAD51 and γH2A.X intensity was plotted in GraphPad Prism (GraphPad Software, Inc).

To evaluate RAD51 expression in patient material, immunofluorescence and immunohistochemistry were performed on deparaffinized, rehydrated sections from archived FFPE patient blocks. Following antibody‐specific epitope retrieval, immunohistochemistry was performed on 2 μm thick tissue sections using the automated Ventana Benchmark (Roche) system. Primary antibodies against RAD51 (Abcam, ab133534 [EPR4030], RRID:AB_2722613) diluted 1/500 and HRP‐coupled secondary antibodies were used. For RAD51 immunofluorescence, 5 μm thick tissue sections were subjected to heat‐mediated antigen retrieval (EDTA buffer, pH 8.4) at 95°C for 20 min and blocked/permeabilized for 1 h at RT. Immunofluorescence was performed with rabbit anti‐RAD51 (Abcam, ab133534 [EPR4030], RRID:AB_2722613) antibodies diluted 1/250 incubated o/n at 4°C followed by three washes in 1× PBS, 45 min incubation with donkey anti‐rabbit Alexa488 antibodies and three washes in 1× PBS. Coverslips were mounted on top of tissue sections with a few drops of FluoroMount aqueous mounting medium (Sigma‐Aldrich, F4680) and protected from light. Imaging was performed in the Leica SP8 confocal microscope using the same settings for all samples in a blinded fashion. Brightness and contrast were adjusted equally to all images in Fiji.

### Statistical analysis and reproducibility

All statistical tests for the *in silico* studies were done using R. Statistical analysis of CIN to compare HRD^high^ and HRD^low^ samples per sarcoma was performed using the Mann–Whitney U‐test. Correlations were calculated following the Spearman's correlation method.

Functional drug testing, immunohistochemistry and immunofluorescence assays were repeated multiple times for each cell model and performed in triplicates. Dose–response curves and fluorescent images are representative of multiple experiments. For immunofluorescence image acquisition and analysis, the investigators were blinded by a third party. Unblinding was performed immediately before final data analysis. No experimental method was used to predetermine sample size. No samples were excluded from the analysis. Data are expected to have normal distribution and are shown as mean ± standard deviation (s.d.) unless otherwise reported in the figure legend. GraphPad Prism was used for statistical analysis. Statistical significance was determined as *P* < 0.05. One‐way ANOVA with Sidak's multiple comparisons test was used for determining statistical significance when comparing staining intensities upon drug treatment. One‐tailed unpaired *t*‐test was used for determining statistical significance when comparing area under the curve in the functional assays.

**The paper explained**

**Problem**

Genomic instability is a hallmark of many cancers. Aberrant proliferation in cancer cells leads to the accumulation of alterations in genes that belong to the homologous recombination (HR) DNA double‐strand break (DSB) repair pathway. Deficiency in HR‐mediated repair (HRR) exacerbates genomic instability and correlates with poor prognosis and development of metastases. Determining HRR deficiency (HRD) in a given tumor is of major clinical relevance as it is associated with therapeutic vulnerabilities. HRD has been widely investigated in certain tumor types such as ovarian and breast cancer but remains greatly unexplored in sarcoma, a rare and heterogeneous group of mesenchymal cancers.

**Results**

Specific sarcoma entities are characterized by high levels of genomic instability signatures and a wide range of molecular alterations in HRR genes, while exhibiting a complex pattern of chromosomal instability features. Furthermore, sarcomas carrying HRD*ness* traits exhibit a distinct SARC‐HRD transcriptional signature that predicts PARP inhibitor sensitivity in patient‐derived *ex vivo* sarcoma models. Concomitantly, HRD^high^ sarcoma cell models lack RAD51 nuclear foci formation upon DNA damage, further evidencing defects in HR‐mediated DNA repair. The WEE1 kinase was discovered as a therapeutic vulnerability for sarcomas with HRD*ness* traits. Finally, we demonstrated clinical benefit of combining DNA damaging agents and inhibitors of DNA repair pathways in patient‐derived *ex vivo* cell models and in a leiomyosarcoma patient.

**Impact**

This work provides the most comprehensive analysis of HRD*ness* in sarcoma to date and offers strategies to successfully treat sarcoma patients. The use of an HRR‐CIN score or the newly identified SARC‐HRD gene expression signature might enhance the identification of patients that benefit from DNA damage and DNA repair‐based therapies to personalize treatment for sarcoma patients.

## Author contributions

**Lara Planas‐Paz:** Conceptualization; data curation; formal analysis; supervision; funding acquisition; validation; investigation; visualization; writing—original draft; project administration; writing—review and editing. **Alicia Pliego‐Mendieta:** Conceptualization; data curation; software; formal analysis; investigation; visualization; methodology; writing—original draft; writing—review and editing. **Catherine Hagedorn:** Validation; investigation; methodology; writing—review and editing. **Domingo Aguilera‐Garcia:** Software; formal analysis; methodology; writing—review and editing. **Martina Haberecker:** Resources; validation; investigation; writing—review and editing. **Fabian Arnold:** Conceptualization; data curation; writing—review and editing. **Marius Herzog:** Validation; writing—review and editing. **Lorenz Bankel:** Resources; writing—review and editing. **Roman Guggenberger:** Formal analysis; investigation; writing—review and editing. **Sabrina Steiner:** Validation; writing—review and editing. **Yanjiang Chen:** Writing—review and editing. **Abdullah Kahraman:** Supervision; methodology; writing—review and editing. **Martin Zoche:** Writing—review and editing. **Mark A Rubin:** Resources; writing—review and editing. **Holger Moch:** Resources; writing—review and editing. **Christian Britschgi:** Resources; writing—review and editing. **Chantal Pauli:** Conceptualization; resources; data curation; formal analysis; supervision; funding acquisition; methodology; writing—original draft; project administration; writing—review and editing.

## Disclosure and competing interests statement

C.P. reports a consulting/advisory role for F. Hoffman‐La Roche AG outside this work. M.A.R. is on the SAB for Neogenomics, Inc., and receives research funding from H. Hoffman‐La Roche AG and Genentech, Inc. No competing interest, financial or otherwise, is declared by all other authors.

## Supporting information



Appendix

Expanded View Figures PDF

Source Data for Expanded View and Appendix

PDF+

Source Data for Figure 1

Source Data for Figure 2

Source Data for Figure 3

Source Data for Figure 4

Source Data for Figure 5

Source Data for Figure 6

## Data Availability

Bulk mRNA sequencing data are available at the Gene Expression Omnibus (GEO) repository under the identifier GSE221532 (https://www.ncbi.nlm.nih.gov/geo/query/acc.cgi?acc=GSE221532).
