# Supplementary material for: Unravelling homologous recombination repair deficiency and therapeutic opportunities in soft tissue and bone sarcoma
Source: EMBO Mol Med. 2023 Feb 13;15(4):e16863. doi: 10.15252/emmm.202216863 (PMC10086583; doi:10.15252/emmm.202216863)
Supplement: Supplementary file 1 — Appendix [file EMMM-15-e16863-s009.pdf]

Table of contents

Appendix Table S1. List of core and associated HRR pathway genes — Page 2

Appendix Table S2. Chromosomal cytobands including HRR genes that exhibit increased CIN in HRD^{high} DDLPS and MPNST compared with HRD^{low} — Page 3

Appendix Table S3. Chromosomal cytobands including HRR genes that exhibit increased CIN in HRD^{high} LMS, ULMS, MFS and UPS compared with HRD^{low} — Page 4

Appendix Figure S1. Absence of RAD51 nuclear foci in olaparib- or trabectedin-treated UPS and MFS sarcoma cells — Page 5

Gene symbol
ATM
ATR
BBC3
BCL2
BCL2L1
BLM
BRCA1
BRCA2
BTBD12
C11orf30
C19orf40
CHEK1
CHEK2
DMC1
EME1
EME2
ERCC4
FANCA
FANCB
FANCC
FANCD2
FANCE
FANCF
FANCG
FANCI
FANCL
FANCM
GEN1
GIYD1
H2AFX
HUS1
LIG1
MDC1
MDM2
MRE11A
MUS81
NBN
OBFC2A
OBFC2B
PALB2
PCNA
PMAIP1
POLD1
POLD2
POLD3
POLD4
PTEN
RAD1
RAD17
RAD50
RAD51
RAD51C
RAD51L1
RAD51L3
RAD52
RAD54B
RAD54L
RAD9A
RBBP8
RPA1
RPA2
RPA3
RTEL1
SHFM1
TEX15
TP53BP1
TREX1
UBE2N
XRCC2
XRCC3

Appendix Table S1. List of core and associated HRR pathway genes

Cytoband chromosome	HRR gene symbol	Sarcoma type	p-value
1_p34.1	RAD54L	DDLPS	1.02E-02
10_q23.31	PTEN	DDLPS	1.14E-04
11_q13.1	MUS81	DDLPS	1.96E-02
11_q13.2	POLD4	DDLPS	1.62E-02
11_q13.2	RAD9A	DDLPS	1.62E-02
11_q13.4	POLD3	DDLPS	8.29E-03
11_q22.3	ATM	DDLPS	8.29E-03
12_p13.33	RAD52	DDLPS	3.16E-03
13_q13.1	BRCA2	DDLPS	1.14E-04
14_q21.2	FANCM	DDLPS	3.99E-03
14_q32.33	XRCC3	DDLPS	4.68E-02
15_q15.1	RAD51	DDLPS	6.53E-05
15_q15.3	TP53BP1	DDLPS	6.53E-05
15_q26.1	BLM	DDLPS	3.25E-05
15_q26.1	FANCI	DDLPS	3.25E-05
16_p13.3	EME2	DDLPS	5.83E-05
16_p13.12	ERCC4	DDLPS	6.83E-04
16_p12.2	PALB2	DDLPS	2.11E-03
16_q24.3	FANCA	DDLPS	1.33E-02
17_p13.3	RPA1	DDLPS	9.17E-07
17_q21.33	EME1	DDLPS	1.17E-03
17_q22	RAD51C	DDLPS	3.19E-04
18_q11.2	RBBP8	DDLPS	8.97E-04
18_q21.32	PMAIP1	DDLPS	2.13E-04
18_q21.33	BCL2	DDLPS	1.20E-05
19_q13.32	BBC3	DDLPS	6.53E-05
19_q13.33	LIG1	DDLPS	2.89E-04
19_q13.33	POLD1	DDLPS	2.89E-04
2_p24.2	GEN1	DDLPS	2.49E-03
2_p16.1	FANCL	DDLPS	5.79E-03
20_p12.3	PCNA	DDLPS	5.38E-03
20_q11.21	BCL2L1	DDLPS	5.17E-04
20_q13.33	RTEL1	DDLPS	3.53E-06
22_q12.1	CHEK2	DDLPS	2.50E-02
22_q13.1	DMC1	DDLPS	2.61E-04
3_p25.3	FANCD2	DDLPS	8.20E-04
3_p21.31	TREX1	DDLPS	3.35E-02
3_q23	ATR	DDLPS	3.35E-02
5_p13.2	RAD1	DDLPS	1.14E-04
5_q13.2	RAD17	DDLPS	1.14E-04
5_q31.1	RAD50	DDLPS	1.51E-03
6_p21.33	MDC1	DDLPS	4.64E-03
6_p21.31	FANCE	DDLPS	1.51E-03
7_p21.3	RPA3	DDLPS	2.13E-04
7_p13	POLD2	DDLPS	1.20E-05
7_p12.3	HUS1	DDLPS	1.05E-05
7_q36.1	XRCC2	DDLPS	1.51E-03
8_q21.3	NBN	DDLPS	1.36E-05
8_q22.1	RAD54B	DDLPS	2.61E-04
9_p13.3	FANCG	DDLPS	3.19E-04
9_q22.32	FANCC	DDLPS	4.63E-05
14_q32.33	XRCC3	MPNST	3.33E-02

Appendix Table S2. Chromosomal cytobands including HRR genes that exhibit increased CIN in HRD^{high} DDLPS and MPNST compared with HRD^{low}. Statistical significance was determined using Mann-Whitney U test and exact p-values are given. Datasets from TCGA-SARC (n = 247) were used.

LMS	
Cytoband	p-value
1_p34.1	0.01446562
3_q23	0.00256647
3_p25.3	0.00638562
5_q31.1	0.01043981
5_p13.2	0.00623314
6_p21.33	0.00440363
7_q36.1	0.00110631
8_q21.3	0.02446355
9_q22.32	0.00014232
9_p13.3	0.00387331
11_q13.2	0.00308489
11_q13.1	0.01672353
14_q32.33	2.82E-05
14_q21.2	0.00734499
15_q26.1	0.01816222
17_q21.33	0.0037196
18_q11.2	0.01358234
19_q13.32	0.01057557
20_q11.21	0.00262609

ULMS	
Cytoband	p-value
2_p16.1	0.01907558
3_q23	0.01286805
3_p25.3	0.01791754
5_q13.2	0.00384605
5_p13.2	0.00668466
6_p21.31	0.03888217
7_p12.3	0.02407407
7_p21.3	0.02303295
8_q21.3	0.01940944
8_q22.1	0.00267555
8_p12	0.0014296
11_q22.3	0.03220516
12_q22	0.00918911
12_q15	0.02709375
12_p13.33	0.03951925
15_q26.1	0.01225472
17_q22	0.01618614
17_q21.33	0.02709375
17_p13.3	0.01563448
18_q21.33	0.00722558
18_q11.2	0.00430602
18_q21.32	0.00723399
20_p12.3	0.01505907
20_q11.21	0.01166003
20_q13.33	0.02727426

MFS	
Cytoband	p-value
1_p35.3	0.0092349
1_p34.1	0.01462218
7_p13	0.01012756
16_p12.2	0.00435036
16_q24.3	0.01008287
16_p13.12	0.00489162
18_q21.33	0.03310827
18_q11.2	0.0147134
20_p12.3	0.03509377
20_q13.33	0.0178588
20_q11.21	0.0302643

UPS	
Cytoband	p-value
1_p35.3	0.01026524
1_p34.1	0.00226768
3_p21.31	0.00079888
3_p25.3	0.00082745
9_q22.32	0.00558509
9_p13.3	0.00203171
11_q13.1	0.03368223
14_q21.2	0.01747337
17_q21.33	0.02961924
17_q22	0.00216882
18_q21.33	0.01406455
18_q21.32	0.01746248
20_q11.21	0.00547747

Appendix Table S3. Chromosomal cytobands including HRR genes that exhibit increased CIN in HRD^{high} LMS, ULMS, MFS and UPS compared with HRD^{low}. Statistical significance was determined using Mann-Whitney U test and exact p-values are given. Datasets from TCGA-SARC (n = 247) were used.

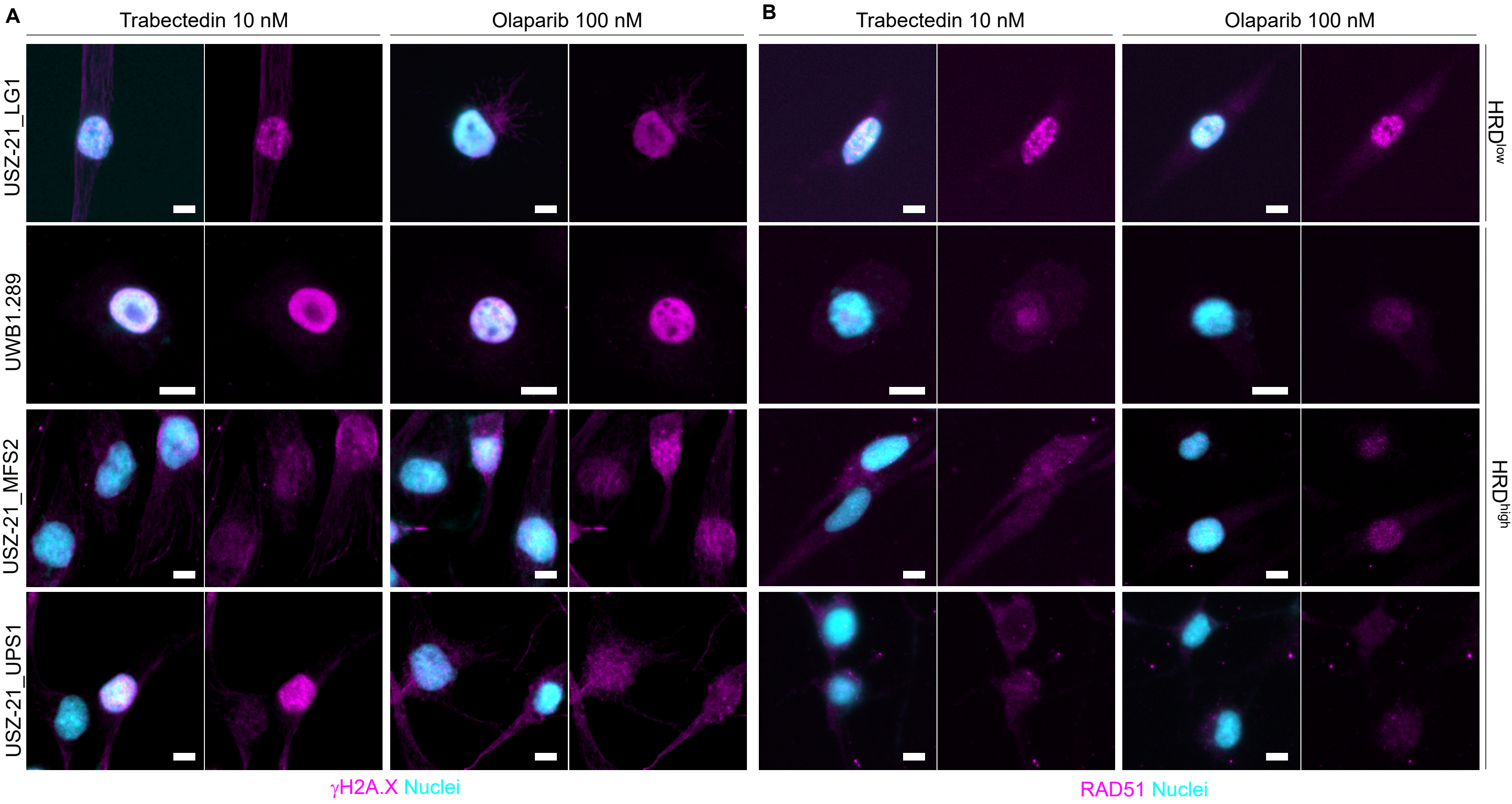

Appendix Figure S1. Absence of RAD51 nuclear foci in olaparib- or trabectedin-treated UPS and MFS sarcoma cells.
A, Immunofluorescence showing nuclear expression of the DNA damage marker γH2A.X (magenta) upon 6 h treatment with 10 nM trabectedin or 100 nM olaparib in HRD^{high} and HRD^{low} sarcoma cell models as well as ovarian carcinoma UWB1.289 cells.
B, Immunofluorescence showing RAD51 nuclear foci (magenta) upon 6 h treatment with 10 nM trabectedin or 100 nM olaparib only in the HRD^{low} sarcoma cell model (USZ-21_LG1). Scale bars, 10 μm.
