# Supplementary material for: Unravelling homologous recombination repair deficiency and therapeutic opportunities in soft tissue and bone sarcoma
Source: EMBO Mol Med. 2023 Feb 13;15(4):e16863. doi: 10.15252/emmm.202216863 (PMC10086583; doi:10.15252/emmm.202216863)
Supplement: Supplementary file 2 — Expanded View Figures PDF [file EMMM-15-e16863-s008.pdf]

Expanded View Figures

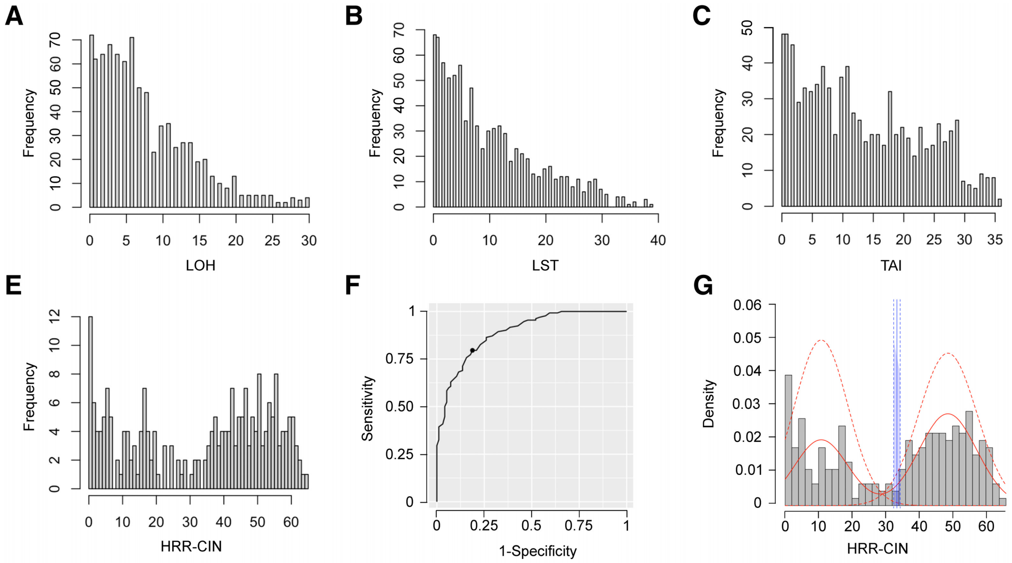

Figure EV1. HRD score cut-off based on genomic alterations in HRR genes (HRR-CIN).

A–D Histograms depicting the frequency of LOH, LST, TAI, and HRD score in sarcoma.

E Bimodal distribution of HRR-CIN in sarcoma.

F Receiver operating characteristic (ROC) curve of HRD scores from TCGA-SARC cohort using HRR-CIN for binary classification.

G Implementation of the Youden index on the ROC curve (F) to select the optimal cut-off value for the HRD score 32 in soft tissue sarcoma. Datasets from TCGA-SARC (n = 247) were used.

Source data are available online for this figure.

 © 2023 The Authors

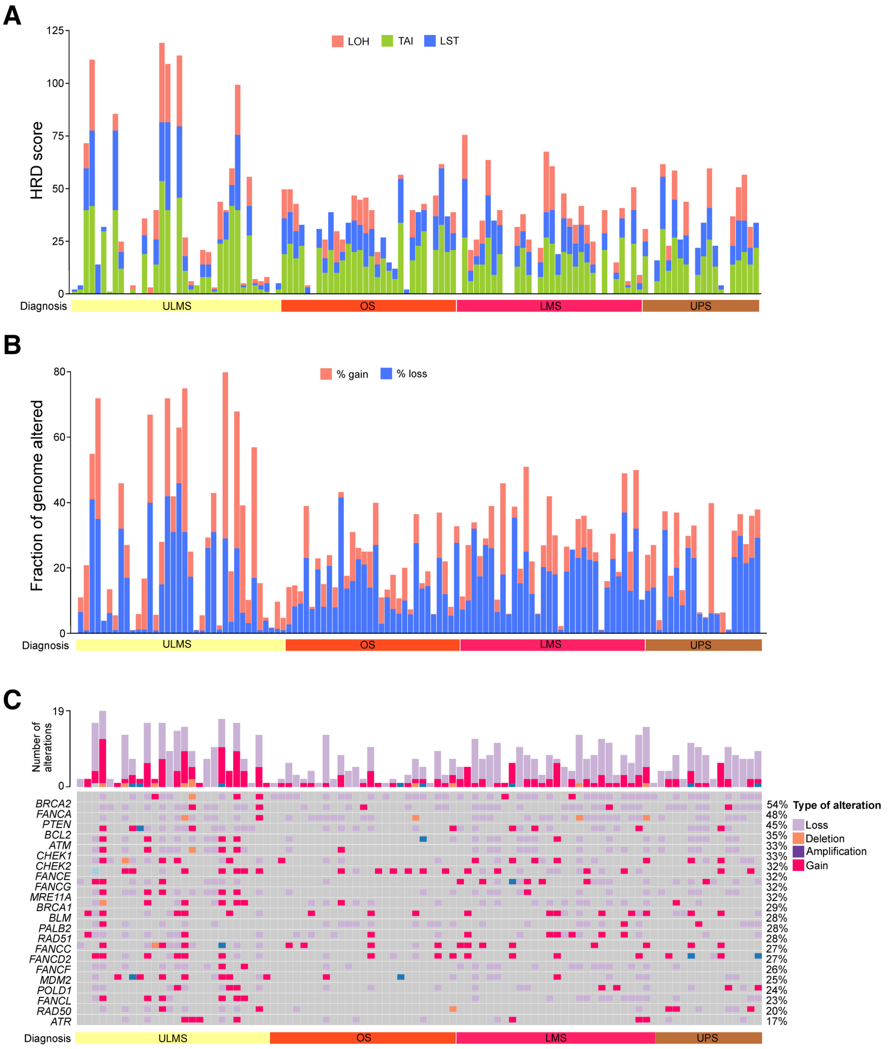

Figure EV2. HRD*ness* in soft tissue and bone sarcoma cohorts is associated with molecular alterations in HRR pathway genes.

A LOH, LST, TAI, and HRD score in OS, LMS, ULMS and UPS cohorts.

B Fraction of genome with gains and losses in OS, LMS, ULMS and UPS cohorts.

C Oncoprint depicting gains and losses in chromosomal regions of HRR genes (included in the Affymetrix Genome-wide Human SNP 6.0 arrays and Oncoscan array) and the total number of alterations in OS, LMS, ULMS and UPS cohorts.

Data information: Datasets from GSE33153, GSE154591, and GSE119043 were used; *n* = 30 OS, 34 ULMS, 30 LMS, and 20 UPS.
Source data are available online for this figure.

Figure EV3. Differential expression of HRR genes and gene set enrichment analysis in HRD^{high} compared with HRD^{low} sarcoma across sarcoma histotypes.

A–F Volcano plot showing HRR genes, enrichment score of HRR genes, GSEA showing hallmark and KEGG pathways enriched in HRD^{high} compared with HRD^{low} UPS (A), MFS (B), ULMS (C), MPNST (D), LMS (E) and DDLPS (F). Normalized enrichment *P*-values < 0.01 for UPS, MFS, MPNST, LMS, and DDLPS. Datasets from TCGA-SARC (*n* = 247) were used.

Source data are available online for this figure.

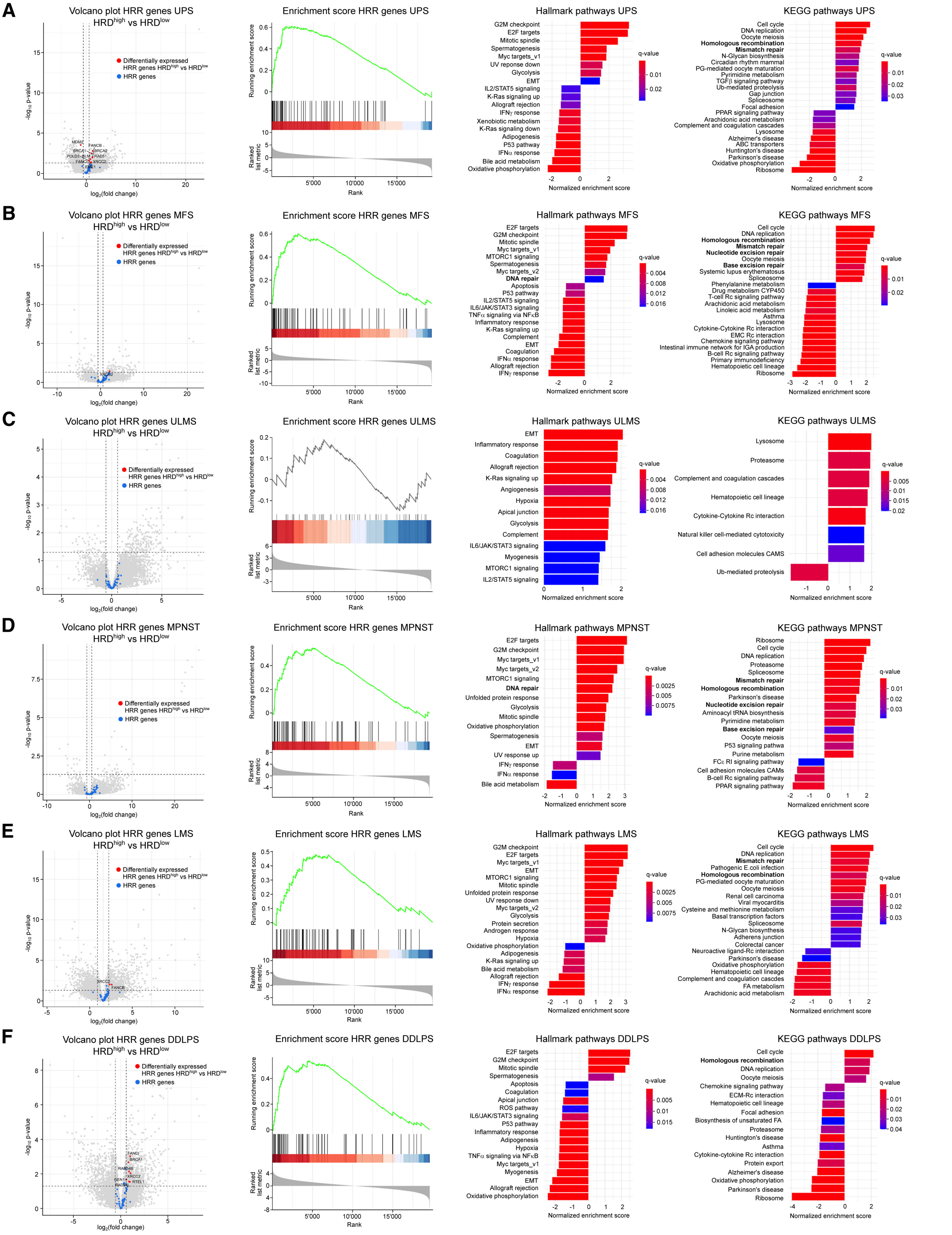

Figure EV3.

 © 2023 The Authors

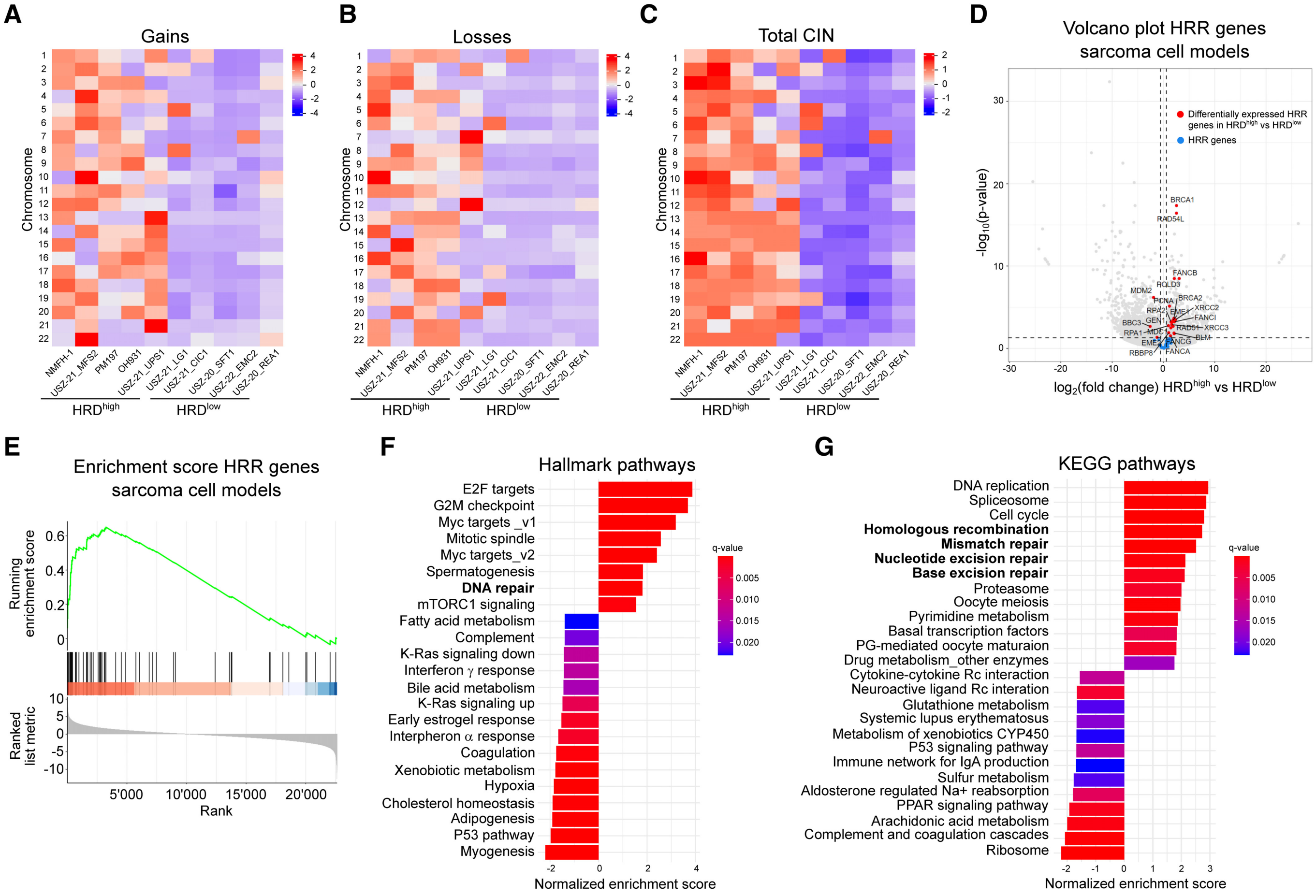

Figure EV4. Molecular characterization of patient-derived sarcoma cell models.

A–C Heatmaps of gains (A), losses (B) and total CIN (C) per chromosome in sarcoma cell models.
D Volcano plot showing enrichment of HRR genes in HRD^{high} compared with HRD^{low} sarcoma cell models.
E Enrichment score of HRR genes in HRD^{high} compared with HRD^{low} sarcoma cell models. Normalized enrichment P-value < 0.01.
F, G GSEA showing hallmark (F) and KEGG (G) pathways enriched in HRD^{high} compared with HRD^{low} sarcoma cell models.

Data information: $n = 5$ HRD^{high} and 5 HRD^{low} sarcoma cell models. Data in (A–C) are mean-centre and scaled.
Source data are available online for this figure.

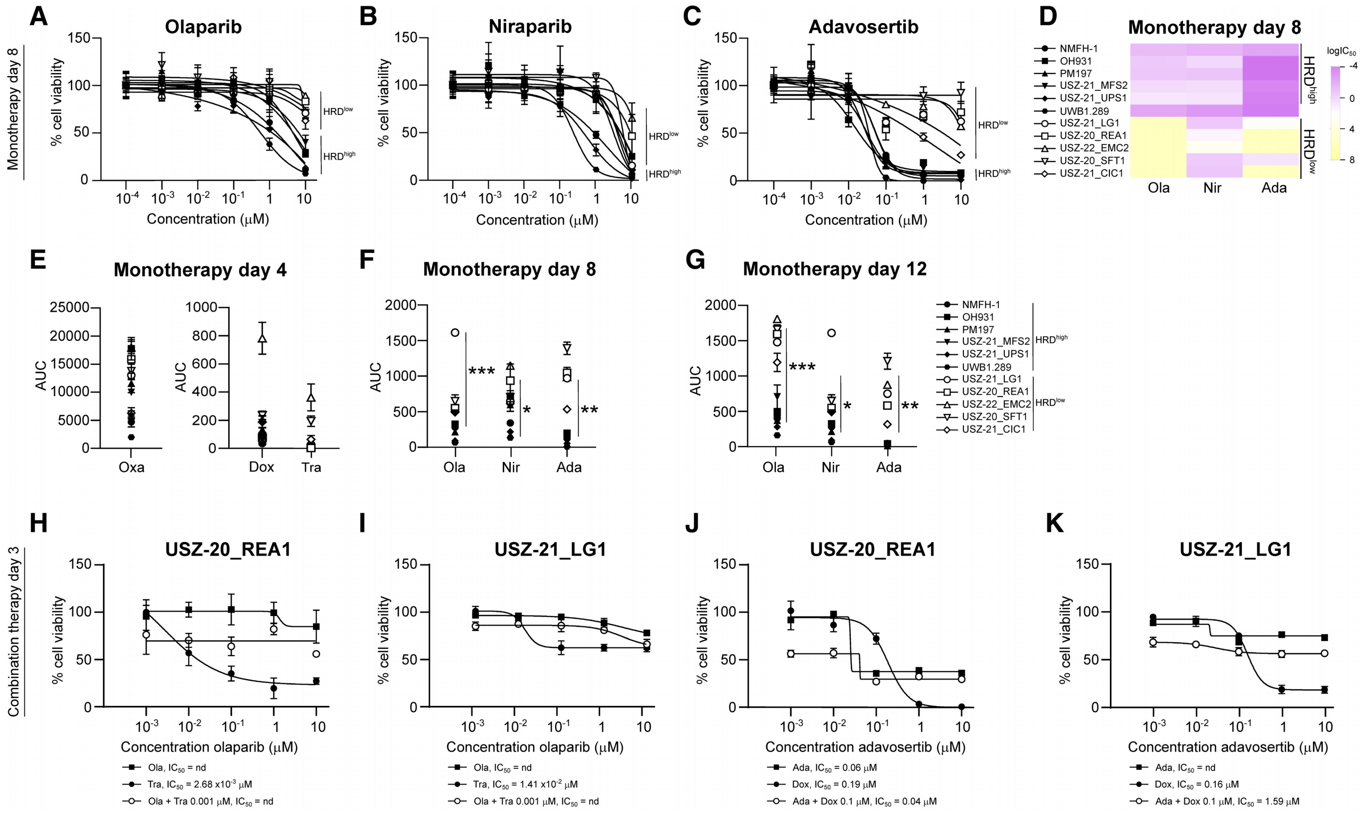

Figure EV5. PARPi and WEE1i monotherapy and combination therapy in patient-derived sarcoma cell models.

A, B HRD^{high} sarcoma cell models show sensitivity to the PARPi olaparib (A) and niraparib (B) when treated for 8 days.
C HRD^{high} sarcoma cell models show sensitivity to the WEE1 inhibitor adavosertib when treated for 8 days.
D Heatmap of IC_{50} showing sensitivity to PARPi and WEE1i in HRD^{high} but not HRD^{low} sarcoma cell models. The ovarian carcinoma cell line UWB1.289 with *BRCA1* mutations was used as positive control for PARPi response and HRD*ness*.
E Area under the curve (AUC) for oxaliplatin, doxorubicin and trabectedin upon 4 days treatment; corresponding dose–response curves depicted in Fig 5E–G.
F AUC for olaparib, niraparib and adavosertib upon 8 days treatment; corresponding dose–response curves depicted in Fig EV5A–C.
G AUC for olaparib, niraparib and adavosertib upon 12 days treatment; corresponding dose–response curves depicted in Fig 5I–K.
H, I HRD^{low} sarcoma cell models treated for 3 days with five doses olaparib alone and in combination with 1 nM trabectedin.
J, K HRD^{low} sarcoma cell models treated for 3 days with five doses adavosertib alone and in combination with 100 nM doxorubicin.

Data information: $n = 5$ HRD^{high} and 5 HRD^{low} sarcoma cell models (A–G), n indicates biological replicates. Data are mean ± s.d. One-tailed unpaired t-test comparing AUC of HRD^{high} and HRD^{low} sarcoma cell models showed significant differences in olaparib, niraparib and adavosertib response in HRD^{high} and HRD^{low} cell models; *$P < 0.05$; **$P < 0.01$; ***$P < 0.0001$; ns, not significant ($P > 0.05$); $P = 2.75 \times 10^{-5}$ (Ola, F), $P = 0.039$ (Nir, F), $P = 1.49 \times 10^{-4}$ (Ada, F), $P = 1.17 \times 10^{-5}$ (Ola, G), $P = 0.028$ (Nir, G), $P = 6.82 \times 10^{-4}$ (Ada, G).
Source data are available online for this figure.

 © 2023 The Authors
