# Supplementary figures and images for: Unravelling homologous recombination repair deficiency and therapeutic opportunities in soft tissue and bone sarcoma
Source: EMBO Mol Med. 2023 Feb 13;15(4):e16863. doi: 10.15252/emmm.202216863 (PMC10086583; doi:10.15252/emmm.202216863)

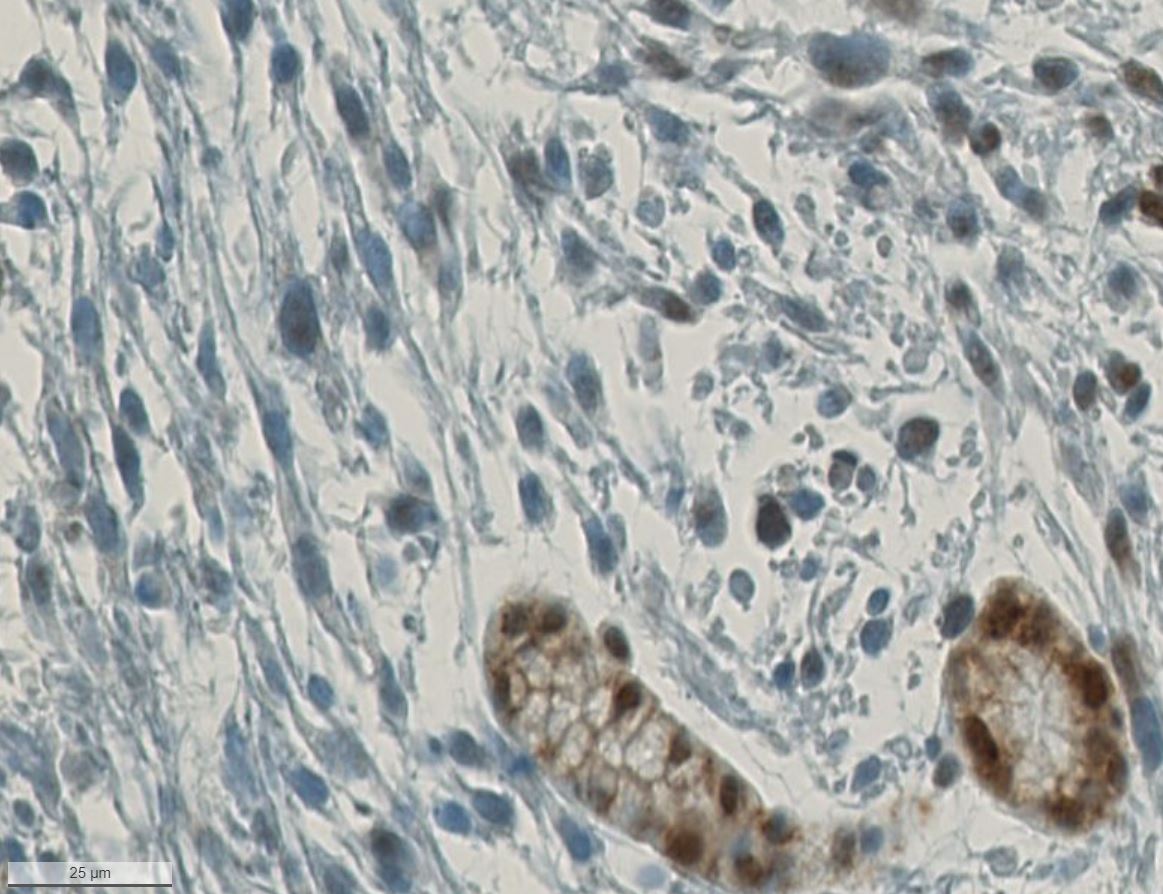

Supplement: Supplementary file 10 — Source Data for Figure 6 [file EMMM-15-e16863-s001.zip › Fig6/Fig6J.jpg]

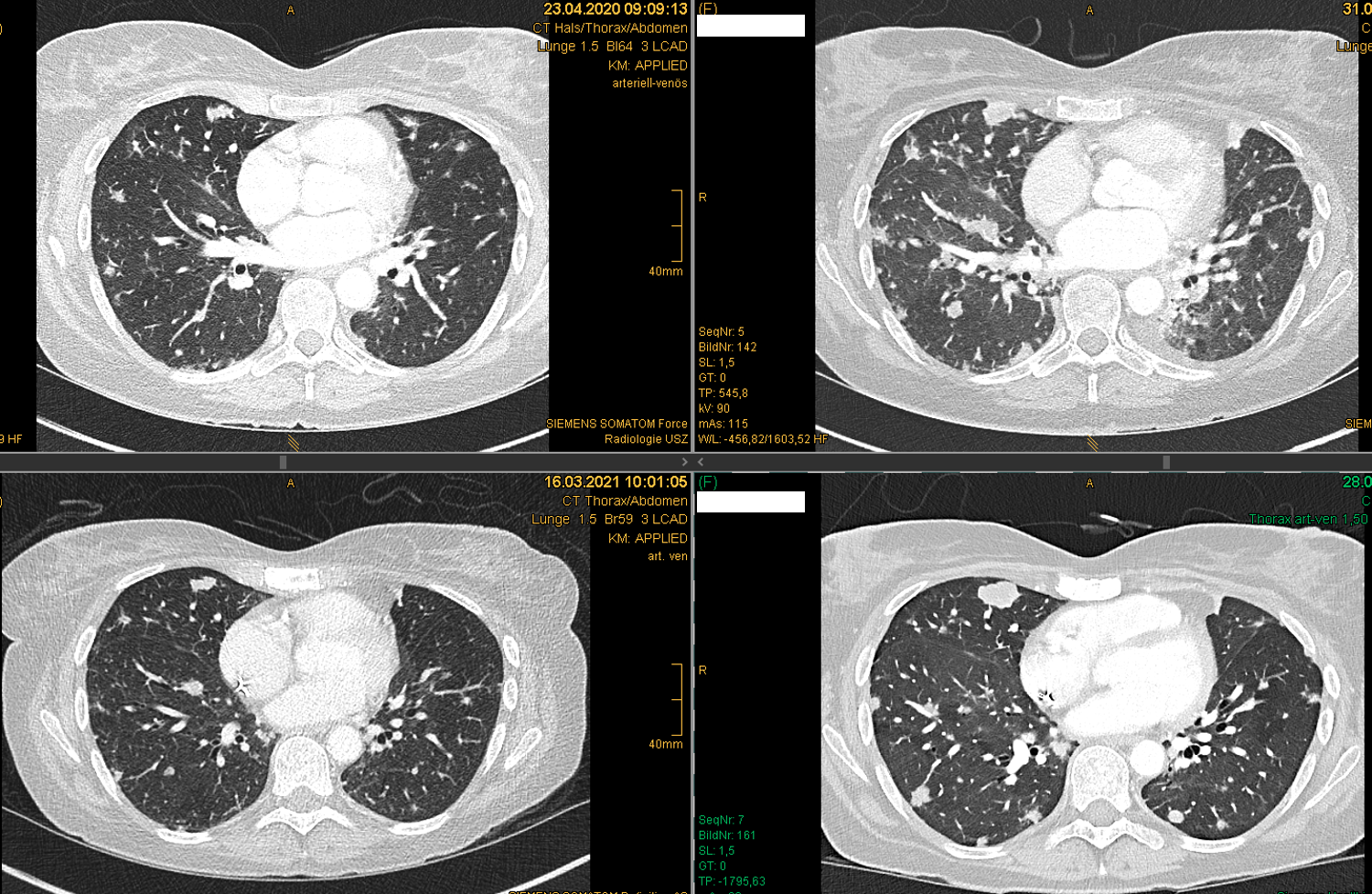

Supplement: Supplementary file 10 — Source Data for Figure 6 [file EMMM-15-e16863-s001.zip › Fig6/Fig6H.png]

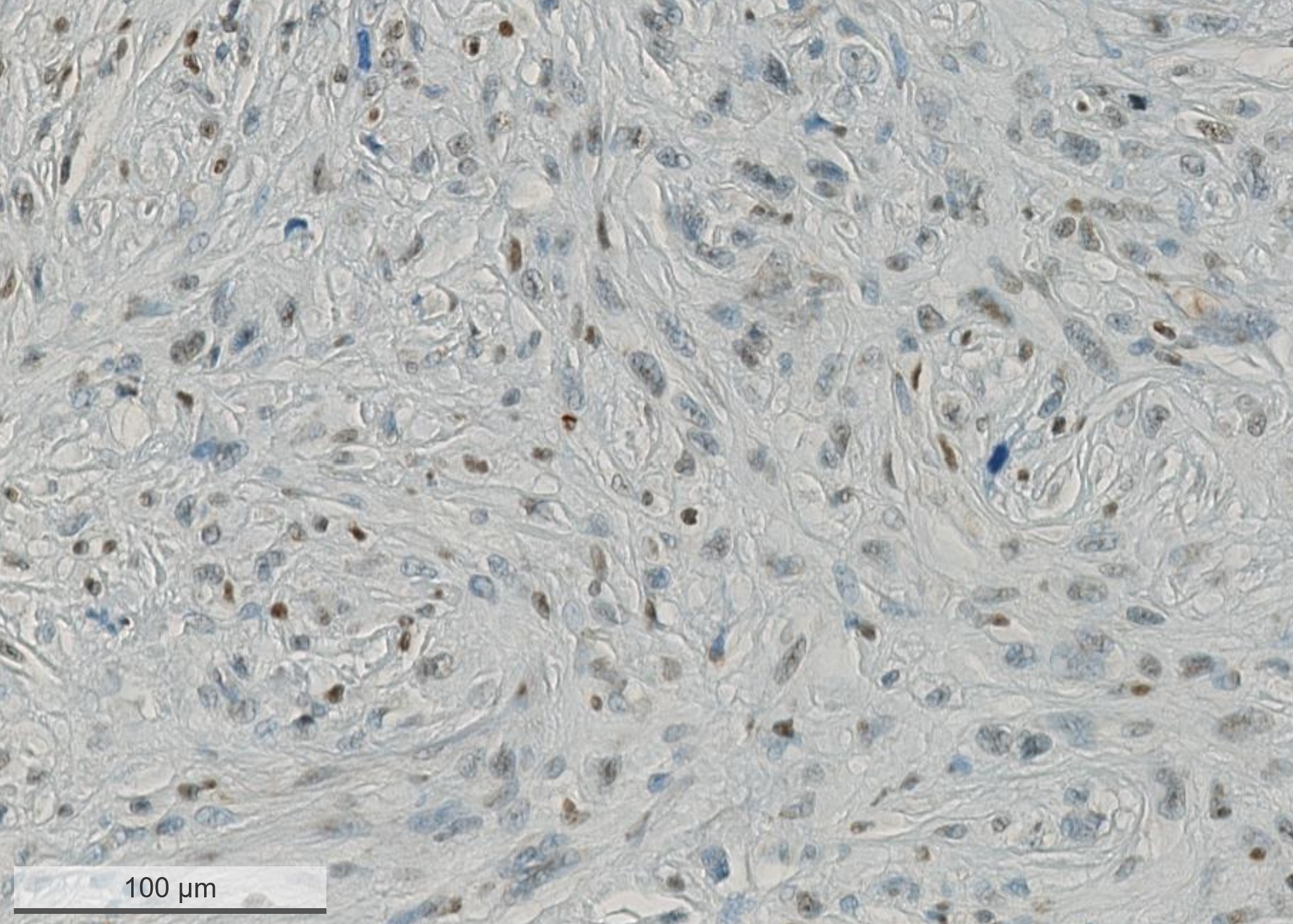

Supplement: Supplementary file 10 — Source Data for Figure 6 [file EMMM-15-e16863-s001.zip › Fig6/Fig6E/Fig6E_MFS2.jpg]

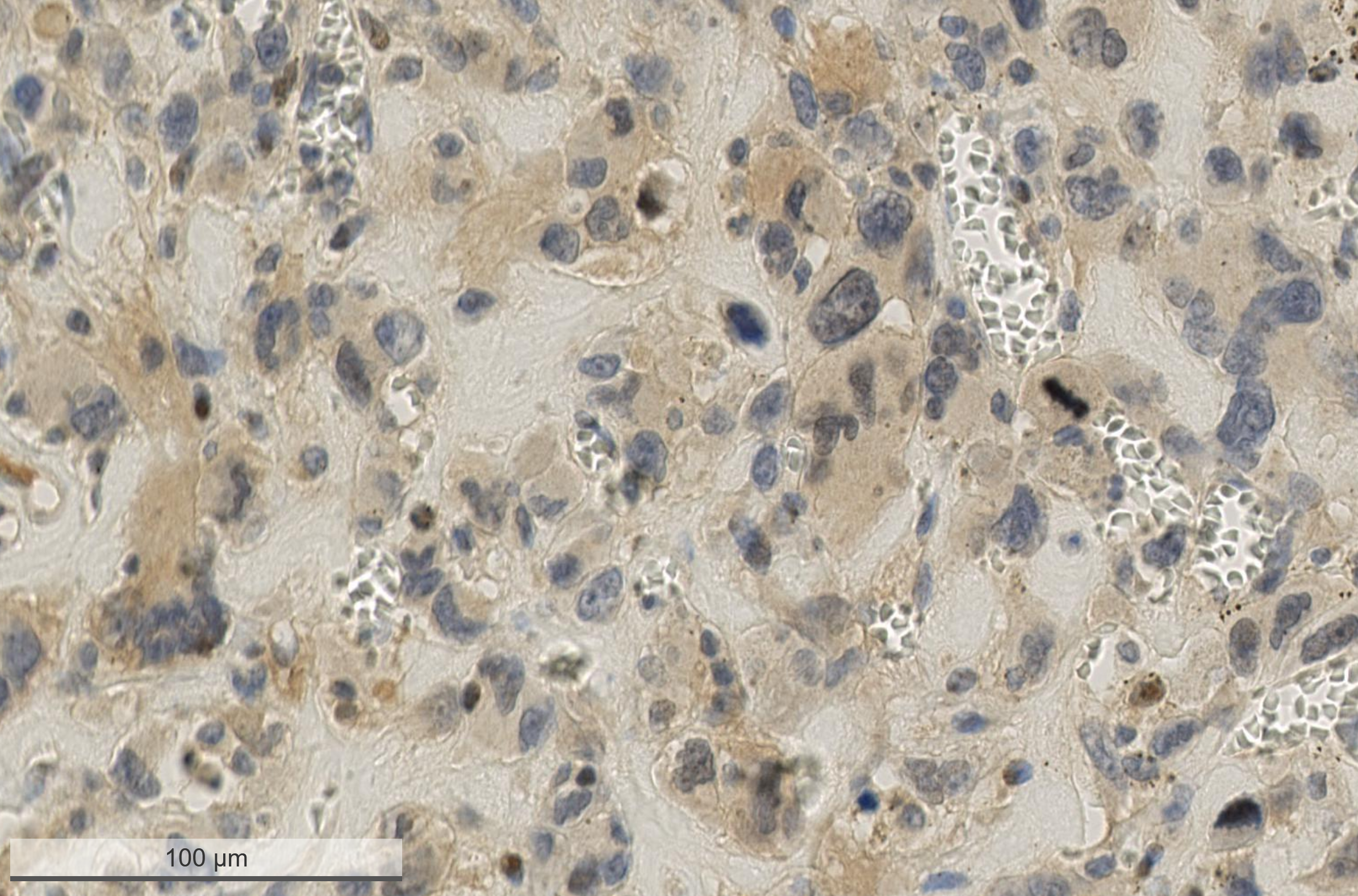

Supplement: Supplementary file 10 — Source Data for Figure 6 [file EMMM-15-e16863-s001.zip › Fig6/Fig6E/Fig6E_UPS1.png]

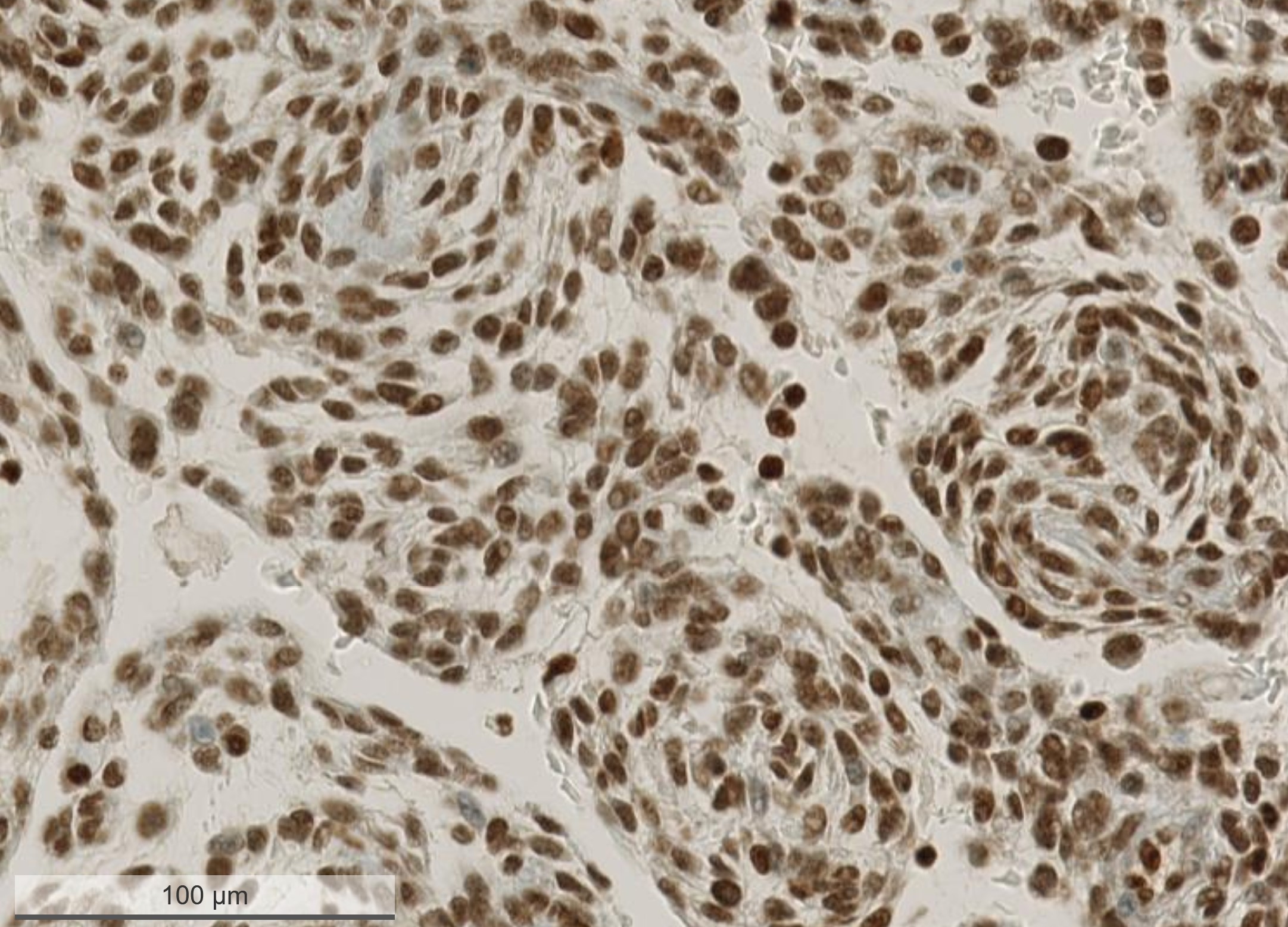

Supplement: Supplementary file 10 — Source Data for Figure 6 [file EMMM-15-e16863-s001.zip › Fig6/Fig6E/Fig6E_LG1.jpg]

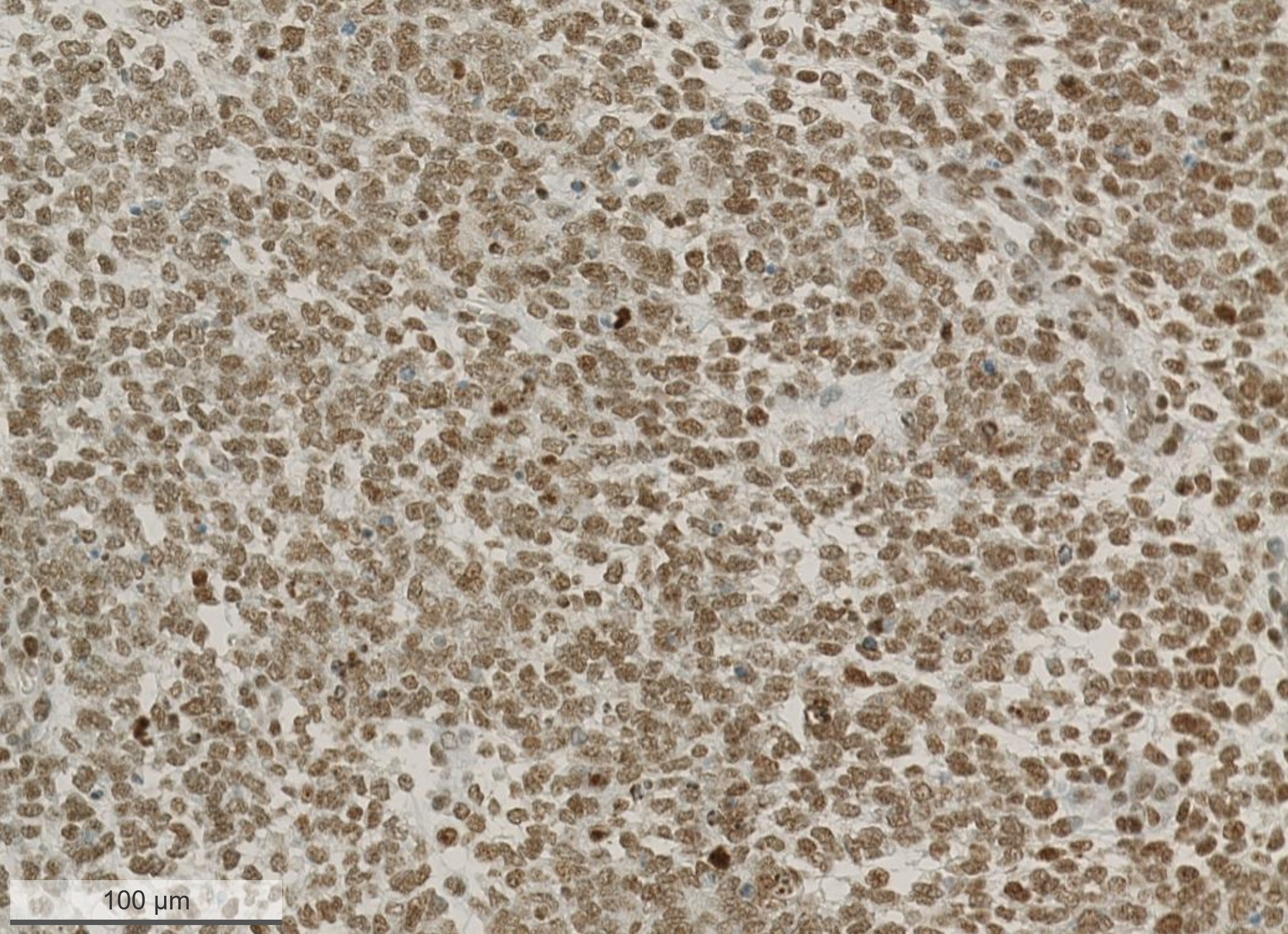

Supplement: Supplementary file 10 — Source Data for Figure 6 [file EMMM-15-e16863-s001.zip › Fig6/Fig6E/Fig6E_REA1.jpg]

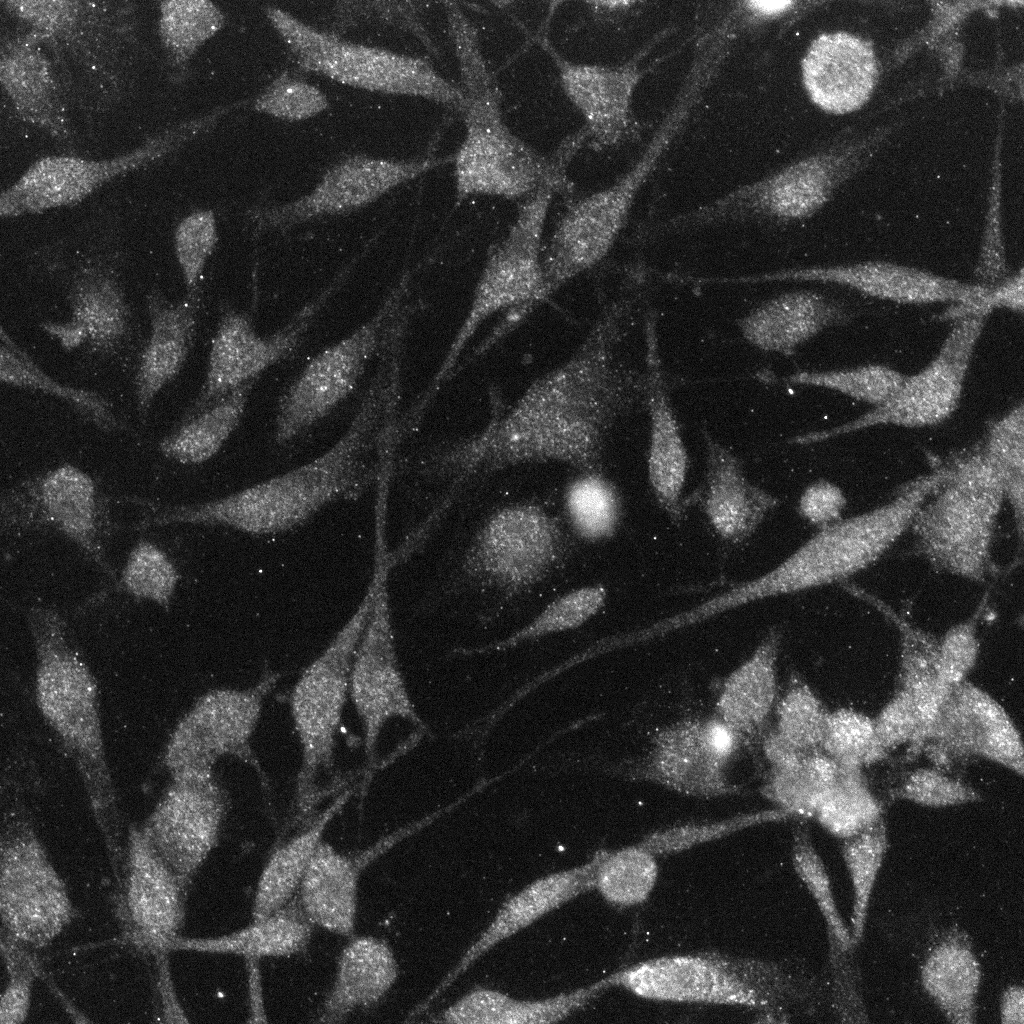

Supplement: Supplementary file 10 — Source Data for Figure 6 [file EMMM-15-e16863-s001.zip › Fig6/Fig6B/Fig6B_MFS2-C-RAD51.tif]

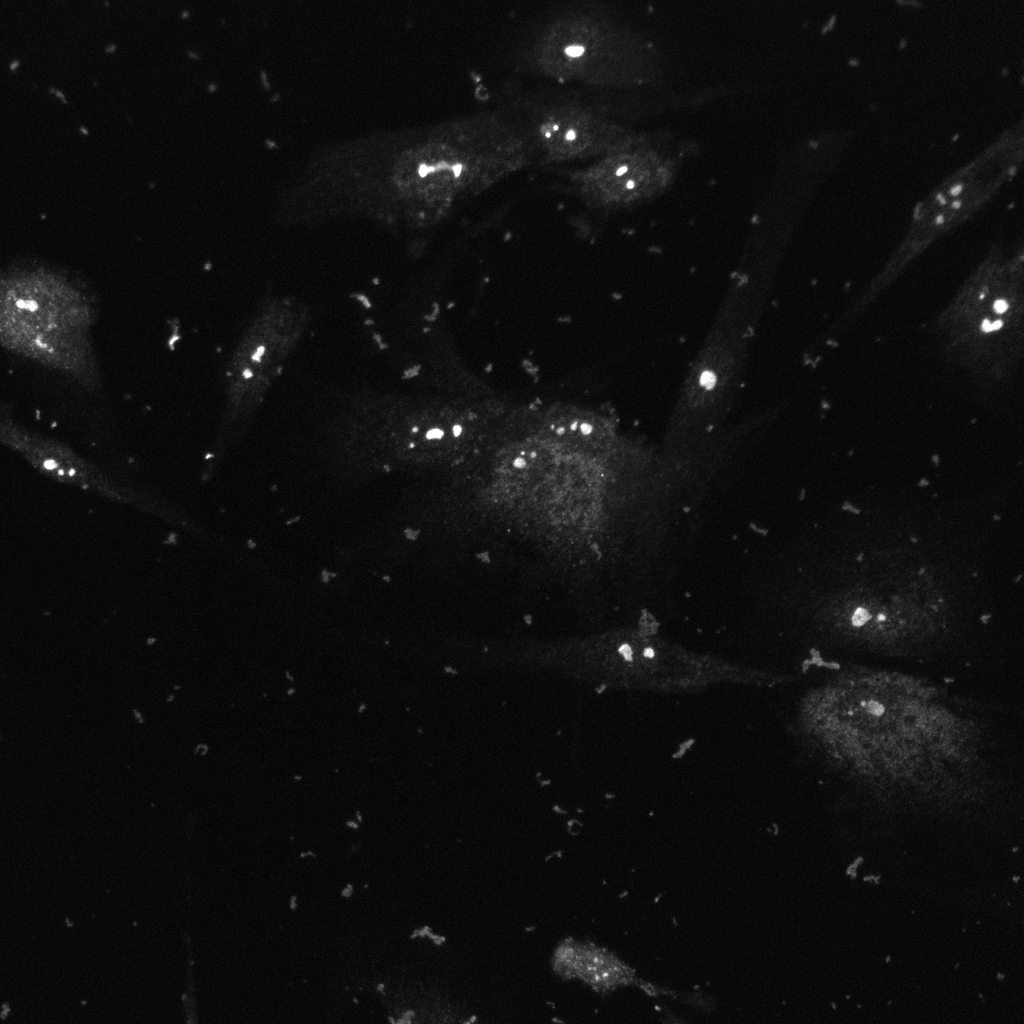

Supplement: Supplementary file 10 — Source Data for Figure 6 [file EMMM-15-e16863-s001.zip › Fig6/Fig6B/Fig6B_LG1-C-RAD51.tif]

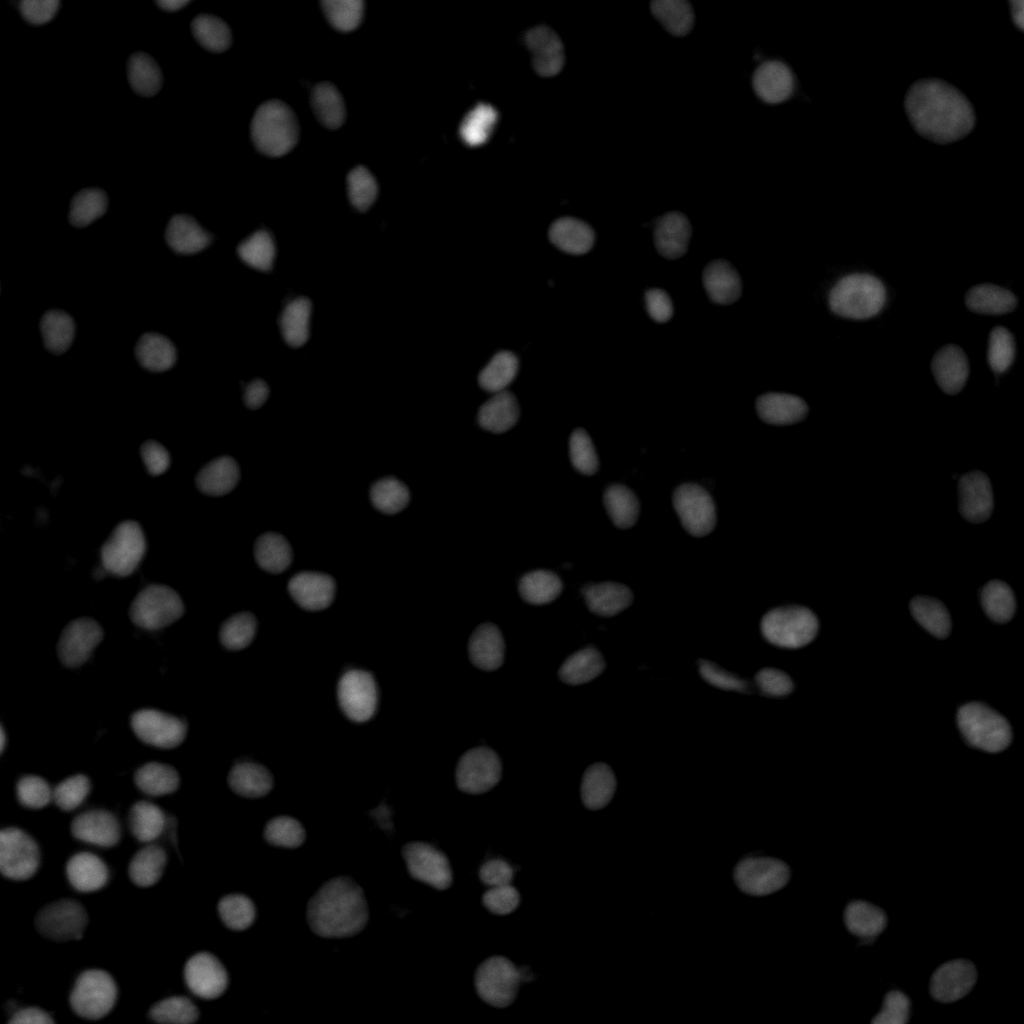

Supplement: Supplementary file 10 — Source Data for Figure 6 [file EMMM-15-e16863-s001.zip › Fig6/Fig6B/Fig6B_UPS1-C-DAPI.tif]

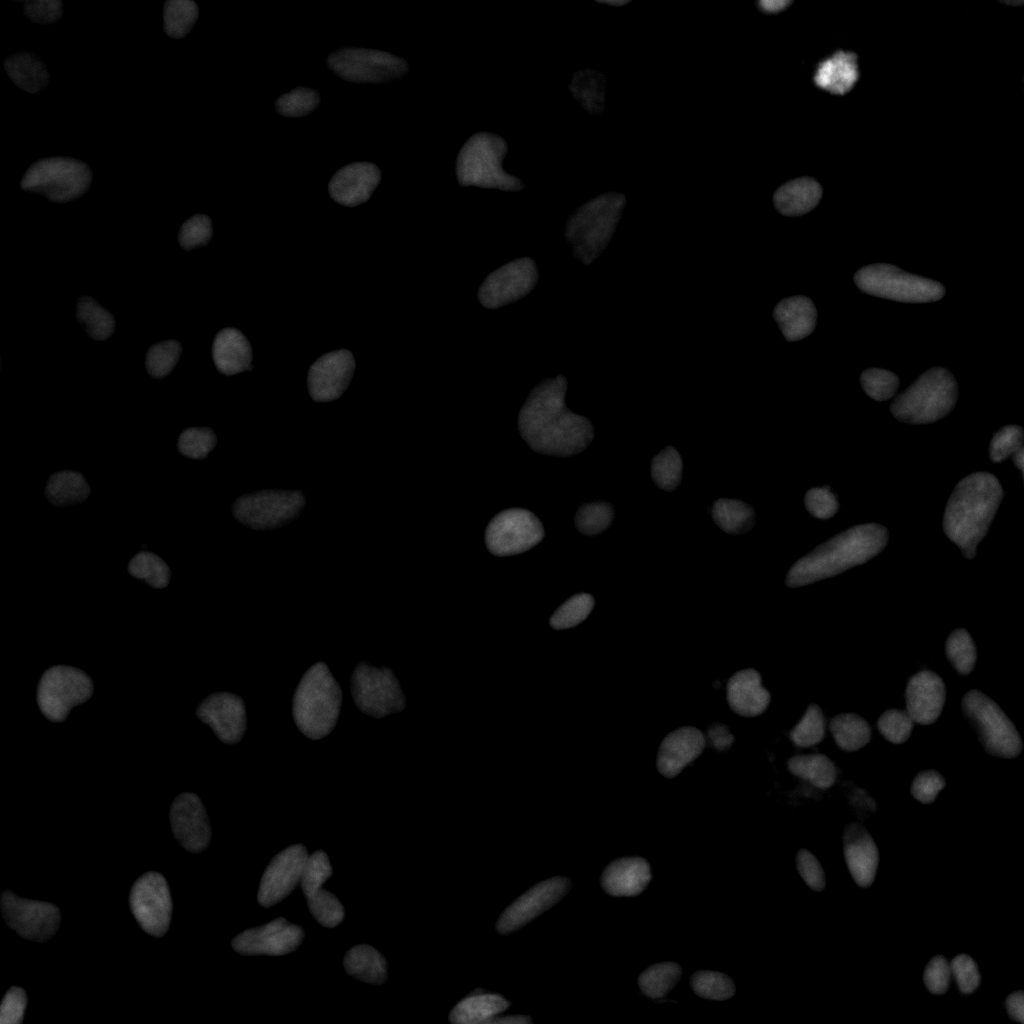

Supplement: Supplementary file 10 — Source Data for Figure 6 [file EMMM-15-e16863-s001.zip › Fig6/Fig6B/Fig6B_MFS2-C-DAPI.tif]

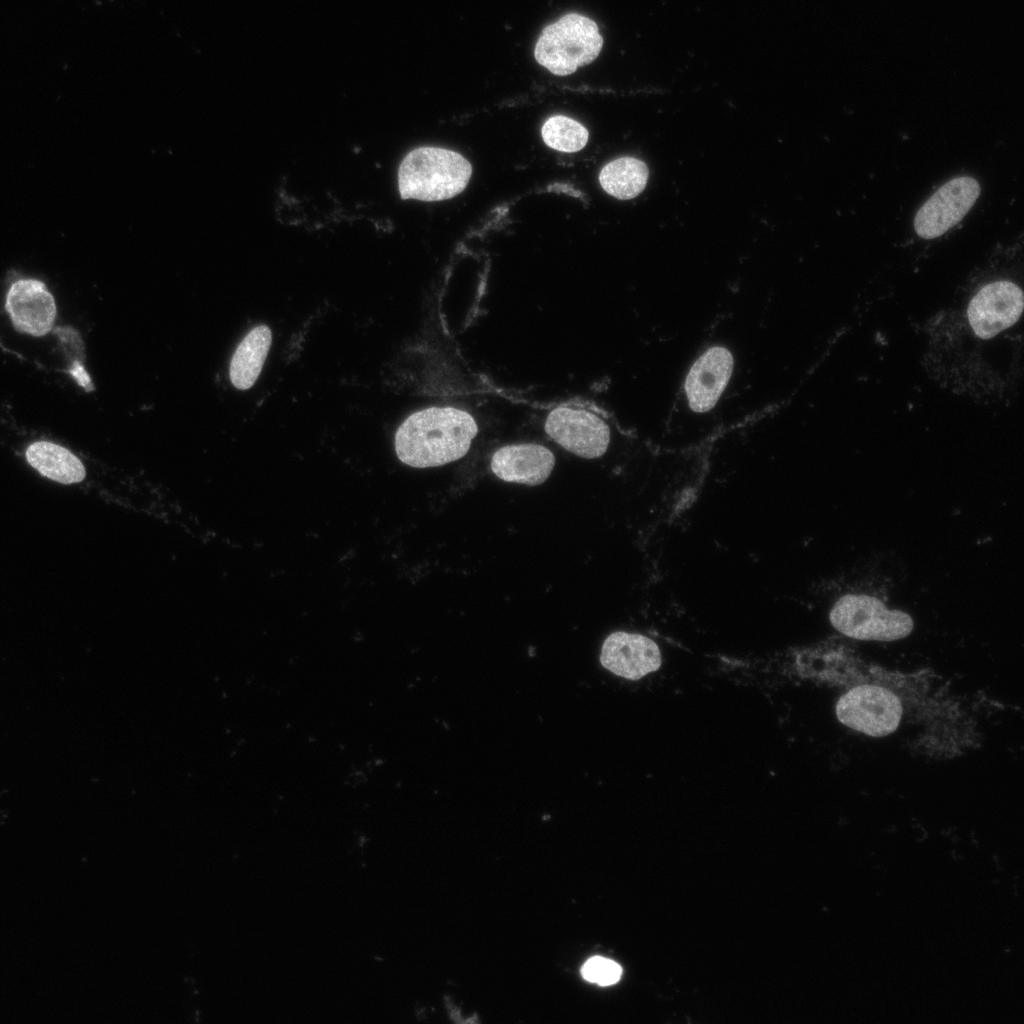

Supplement: Supplementary file 10 — Source Data for Figure 6 [file EMMM-15-e16863-s001.zip › Fig6/Fig6B/Fig6B_LG1-C-DAPI.tif]

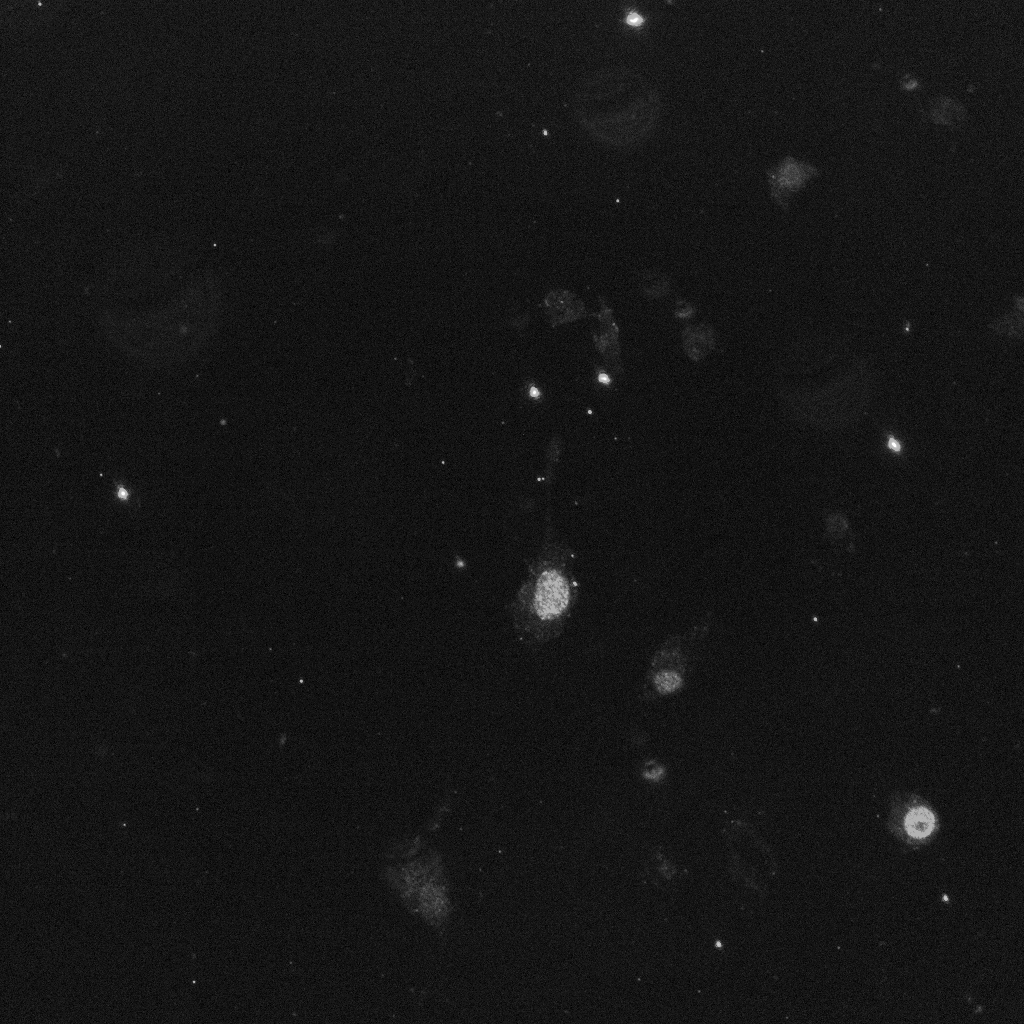

Supplement: Supplementary file 10 — Source Data for Figure 6 [file EMMM-15-e16863-s001.zip › Fig6/Fig6B/Fig6B_UWB1-C-RAD51.tif]

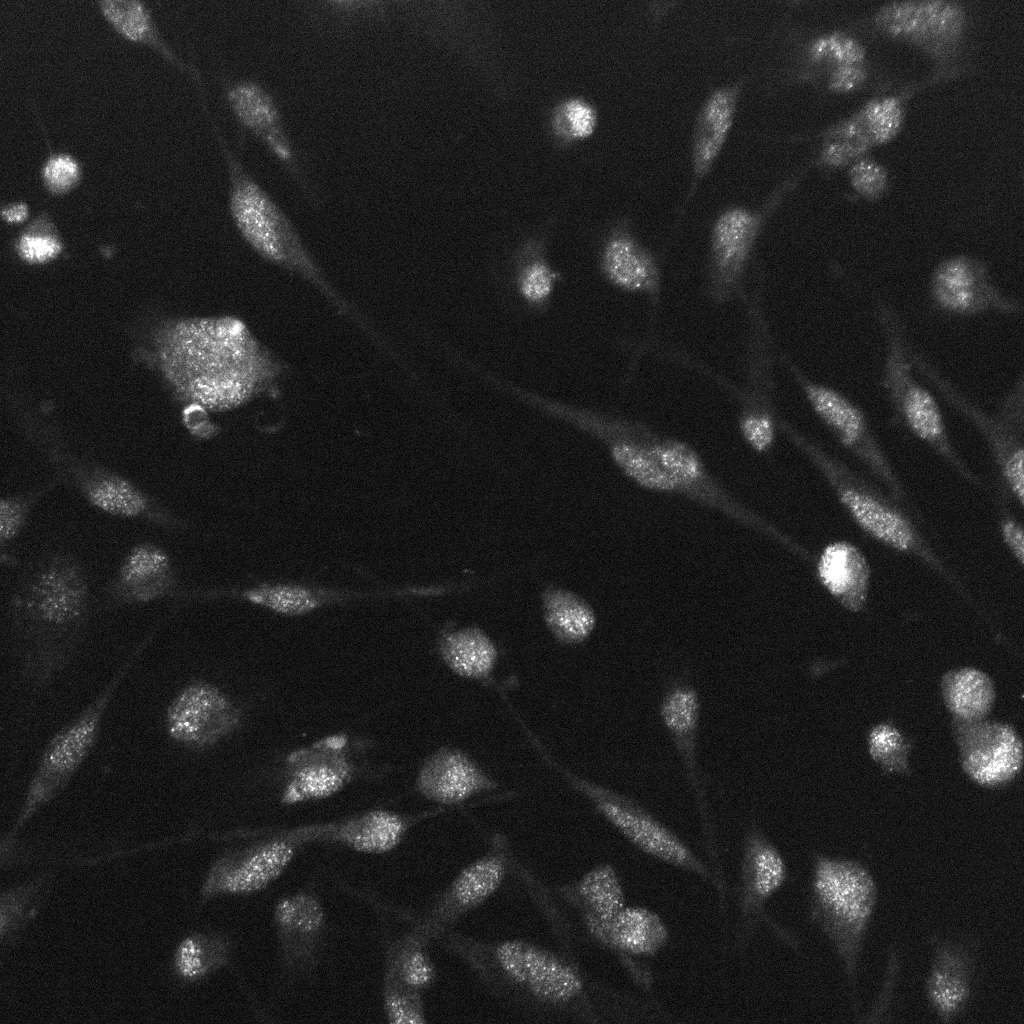

Supplement: Supplementary file 10 — Source Data for Figure 6 [file EMMM-15-e16863-s001.zip › Fig6/Fig6B/Fig6B_MFS2-TO-RAD51.tif]

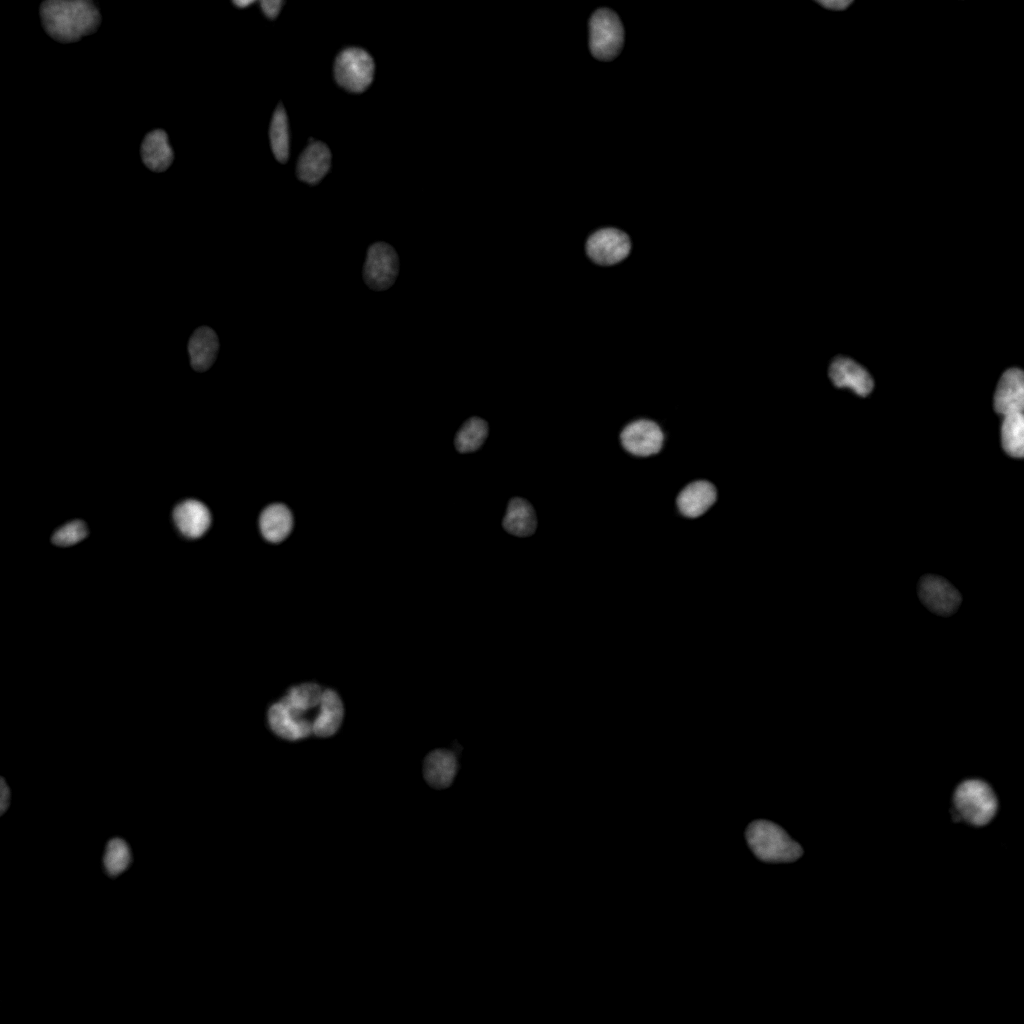

Supplement: Supplementary file 10 — Source Data for Figure 6 [file EMMM-15-e16863-s001.zip › Fig6/Fig6B/Fig6B_UPS1-TO-DAPI.tif]

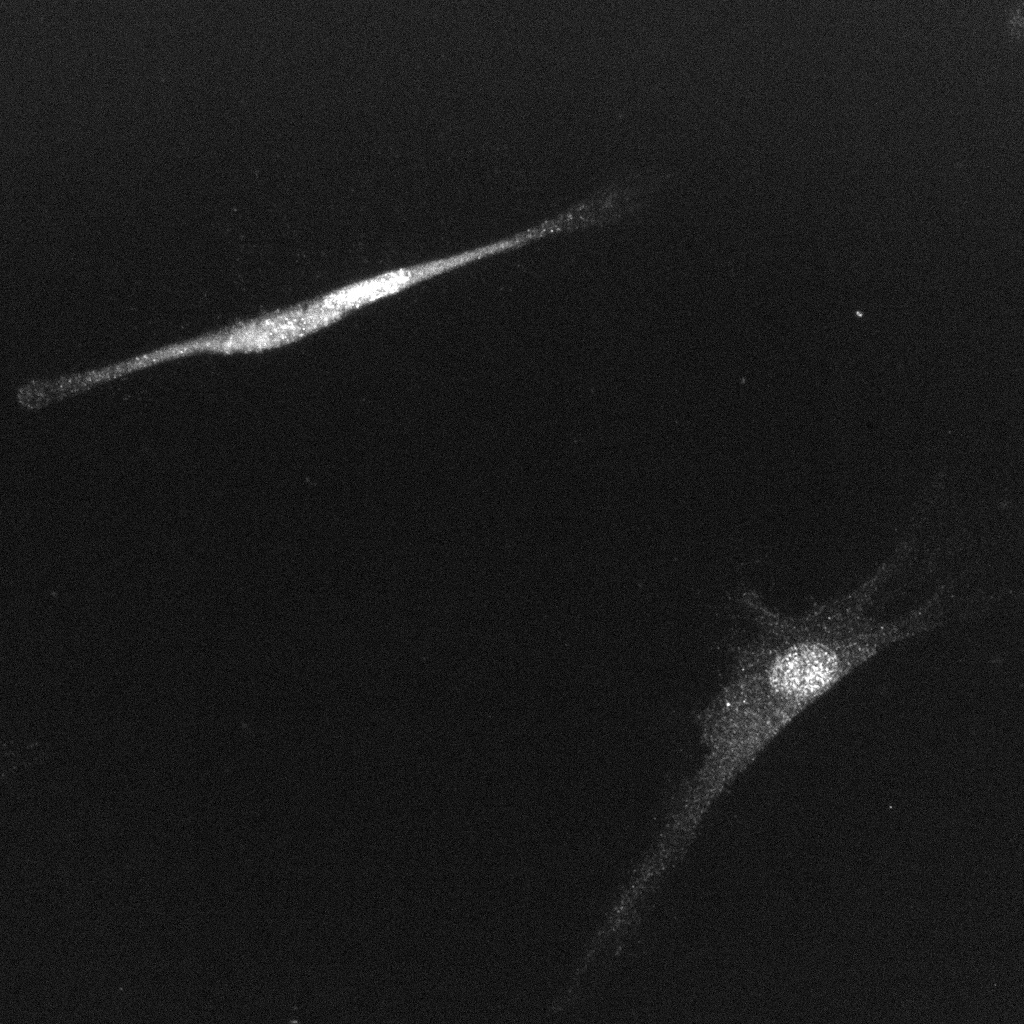

Supplement: Supplementary file 10 — Source Data for Figure 6 [file EMMM-15-e16863-s001.zip › Fig6/Fig6B/Fig6B_LG1-TO-DAPI.tif]

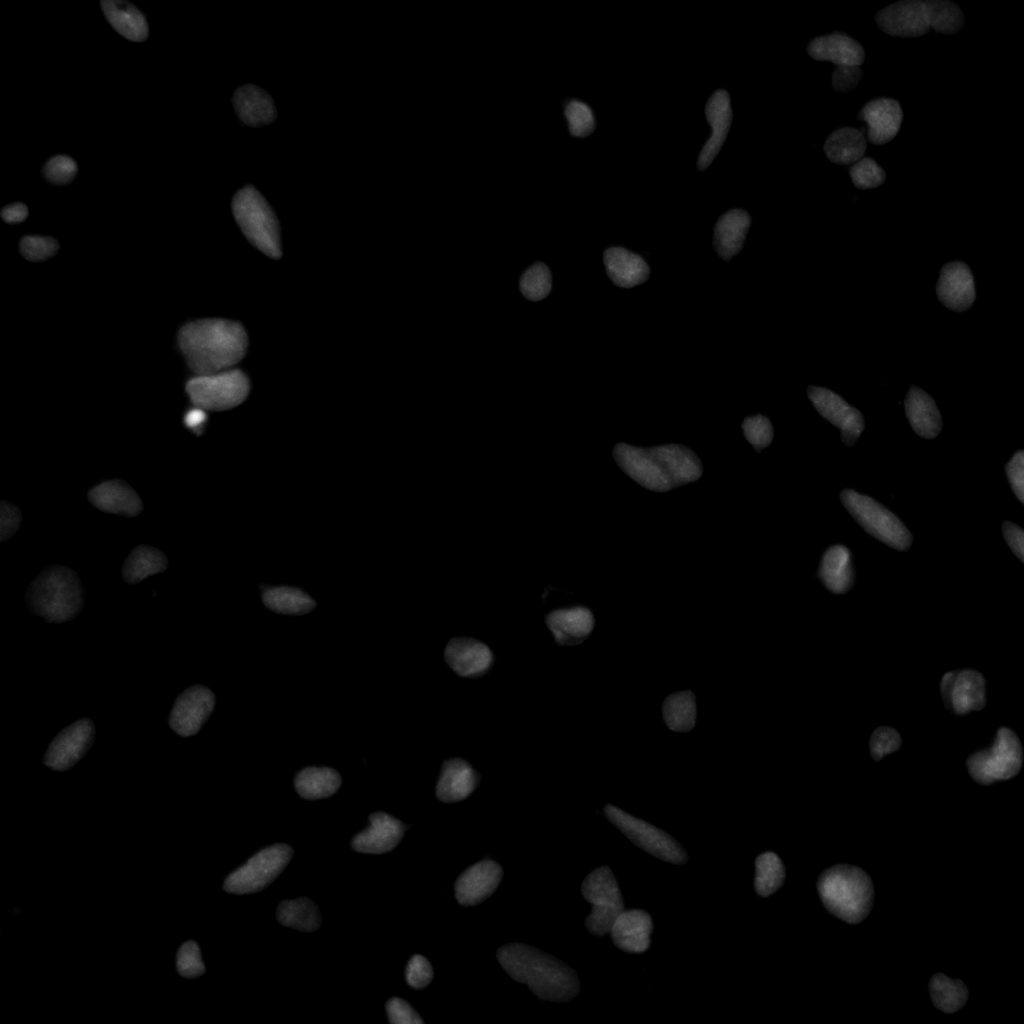

Supplement: Supplementary file 10 — Source Data for Figure 6 [file EMMM-15-e16863-s001.zip › Fig6/Fig6B/Fig6B_MFS2-TO-DAPI.tif]

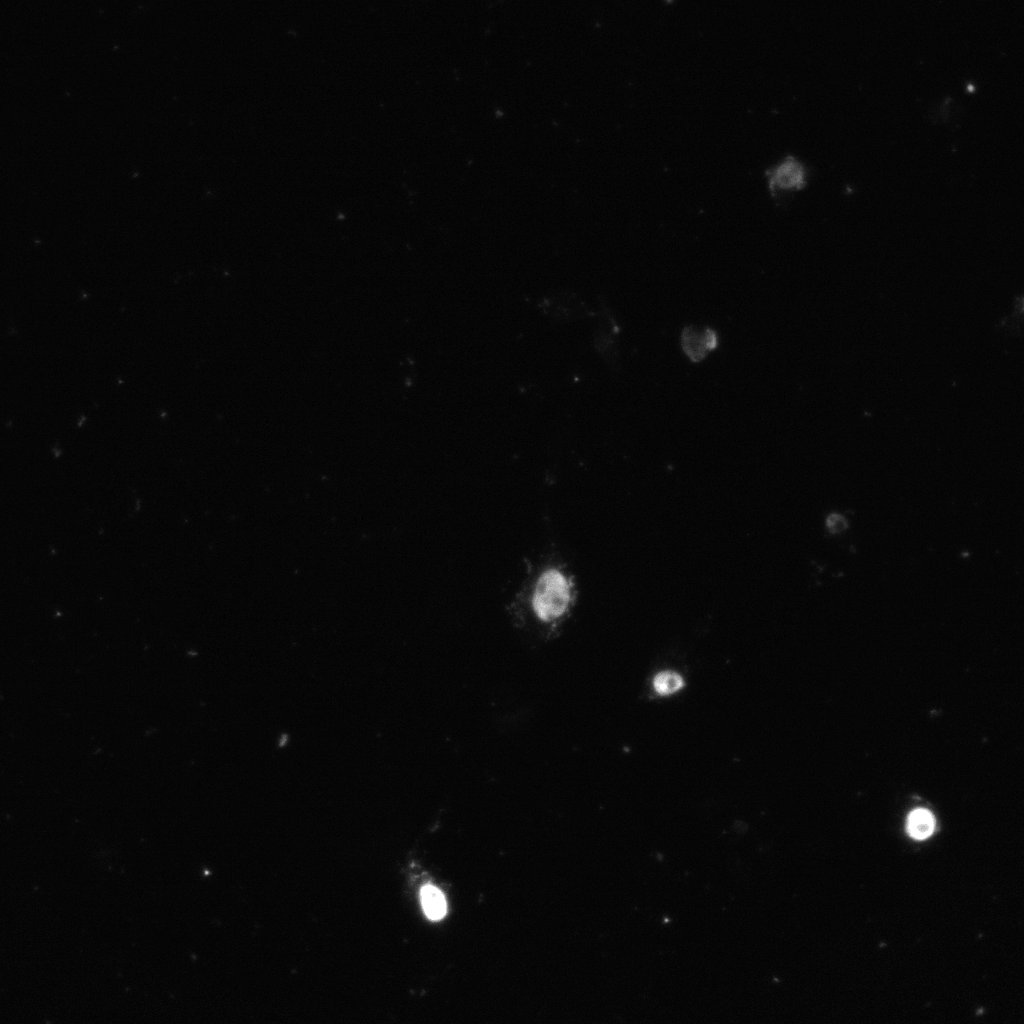

Supplement: Supplementary file 10 — Source Data for Figure 6 [file EMMM-15-e16863-s001.zip › Fig6/Fig6B/Fig6B_UWB1-C-DAPI.tif]

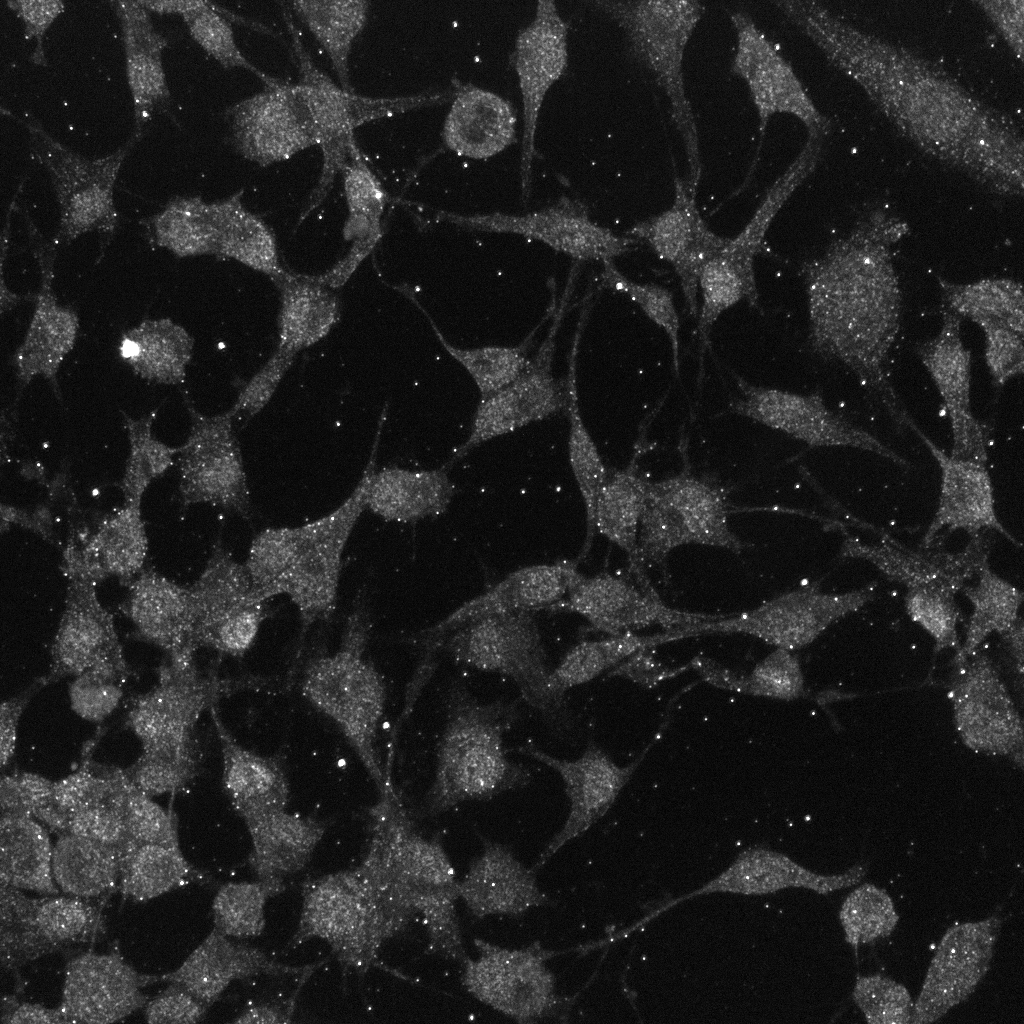

Supplement: Supplementary file 10 — Source Data for Figure 6 [file EMMM-15-e16863-s001.zip › Fig6/Fig6B/Fig6B_UPS1-C-RAD51.tif]

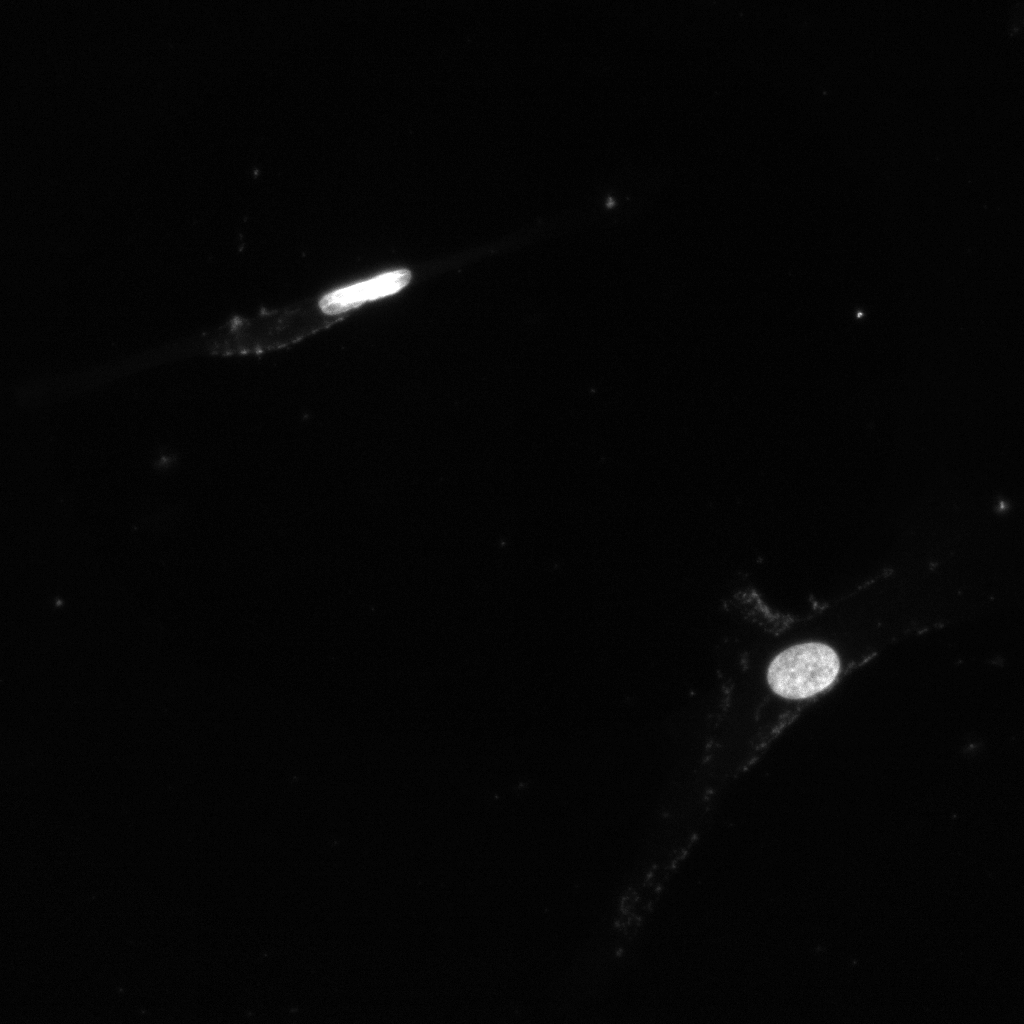

Supplement: Supplementary file 10 — Source Data for Figure 6 [file EMMM-15-e16863-s001.zip › Fig6/Fig6B/Fig6B_LG1-TO-RAD51.tif]

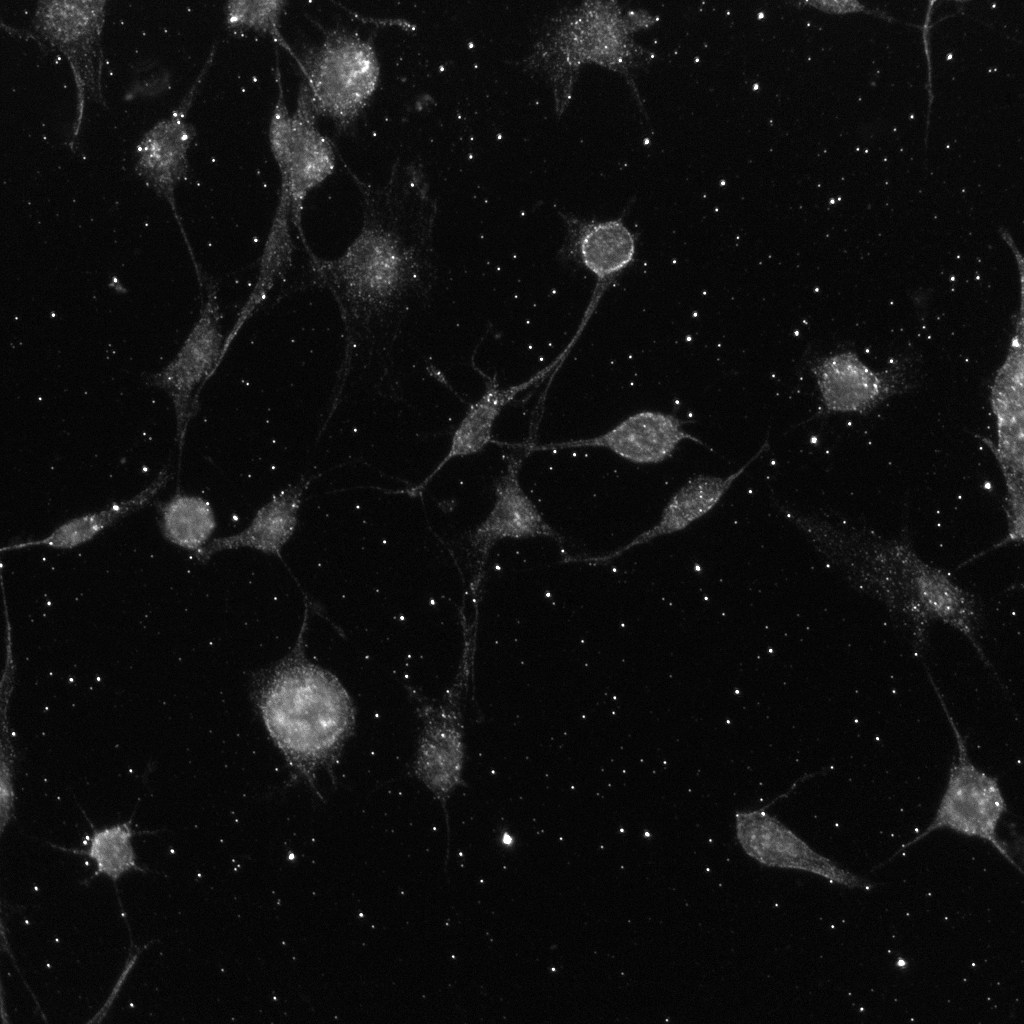

Supplement: Supplementary file 10 — Source Data for Figure 6 [file EMMM-15-e16863-s001.zip › Fig6/Fig6B/Fig6B_UPS1-TO-RAD51.tif]

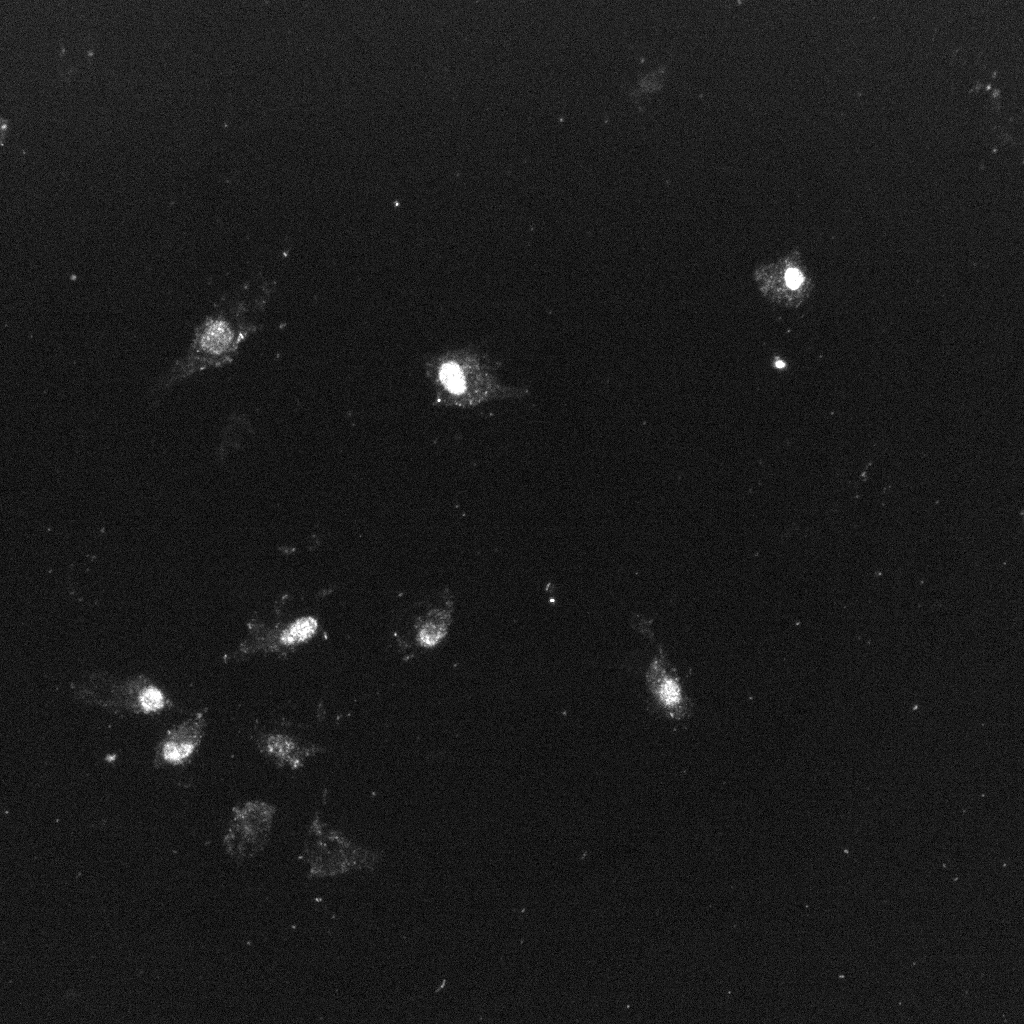

Supplement: Supplementary file 10 — Source Data for Figure 6 [file EMMM-15-e16863-s001.zip › Fig6/Fig6B/Fig6B_UWB1-TO-RAD51.tif]

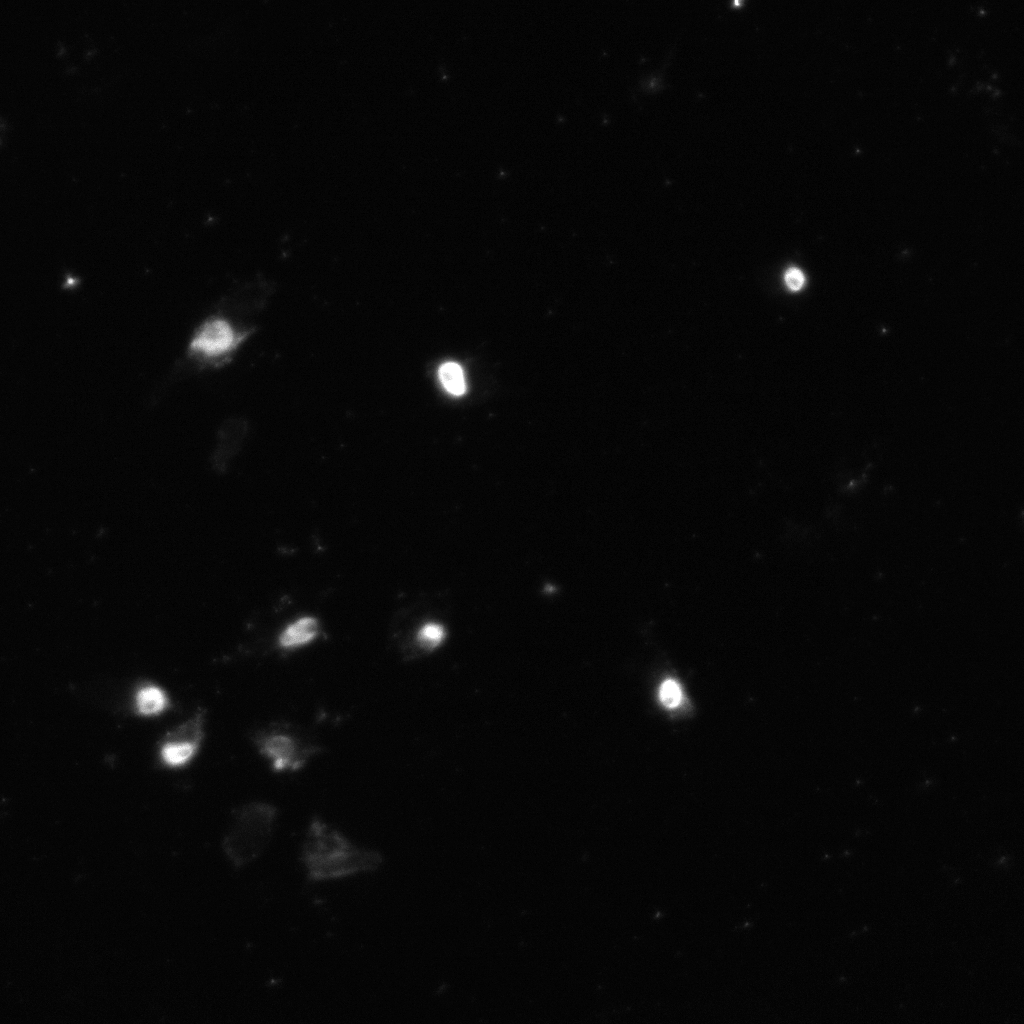

Supplement: Supplementary file 10 — Source Data for Figure 6 [file EMMM-15-e16863-s001.zip › Fig6/Fig6B/Fig6B_UWB1-TO-DAPI.tif]

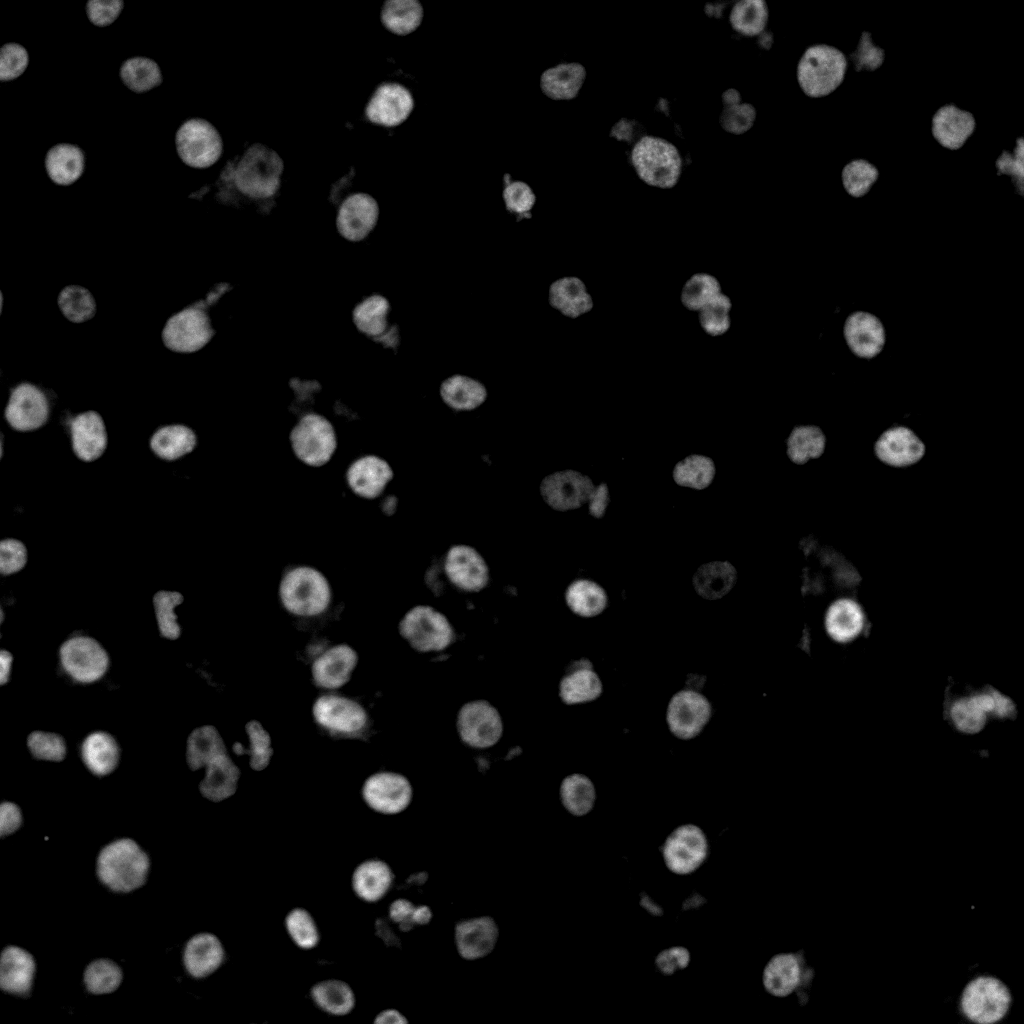

Supplement: Supplementary file 10 — Source Data for Figure 6 [file EMMM-15-e16863-s001.zip › Fig6/Fig6A/Fig6A_UWB1-C-DAPI.tif]

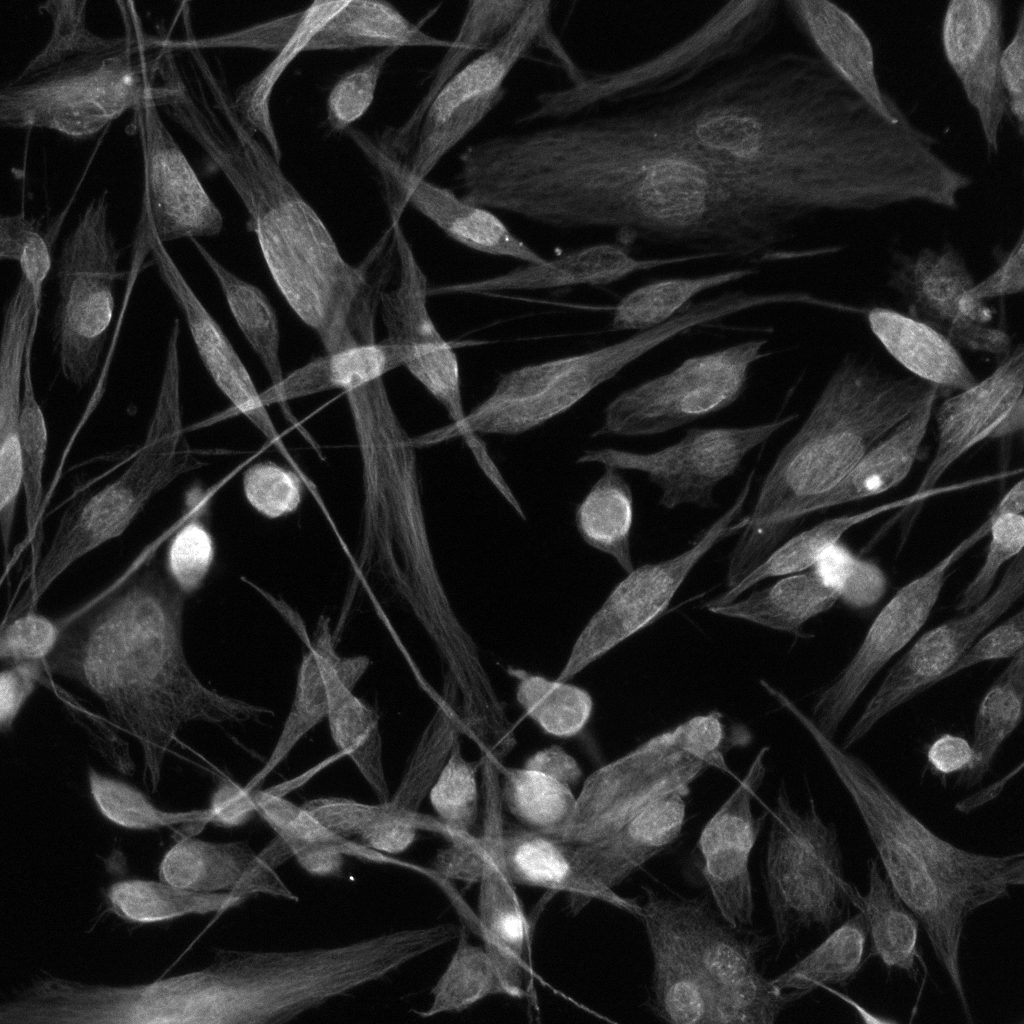

Supplement: Supplementary file 10 — Source Data for Figure 6 [file EMMM-15-e16863-s001.zip › Fig6/Fig6A/Fig6A_MFS2-TO-H2A.tif]

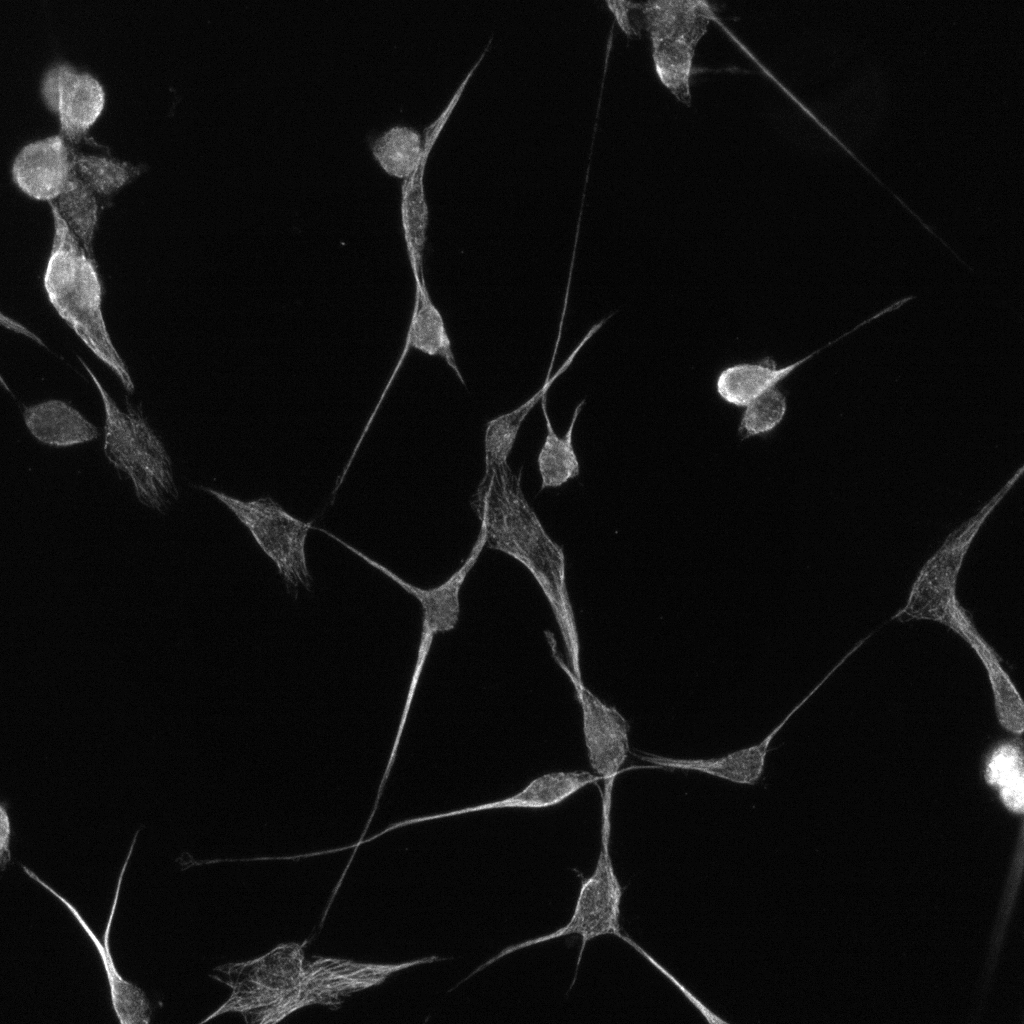

Supplement: Supplementary file 10 — Source Data for Figure 6 [file EMMM-15-e16863-s001.zip › Fig6/Fig6A/Fig6A_UPS1-C-H2A.tif]

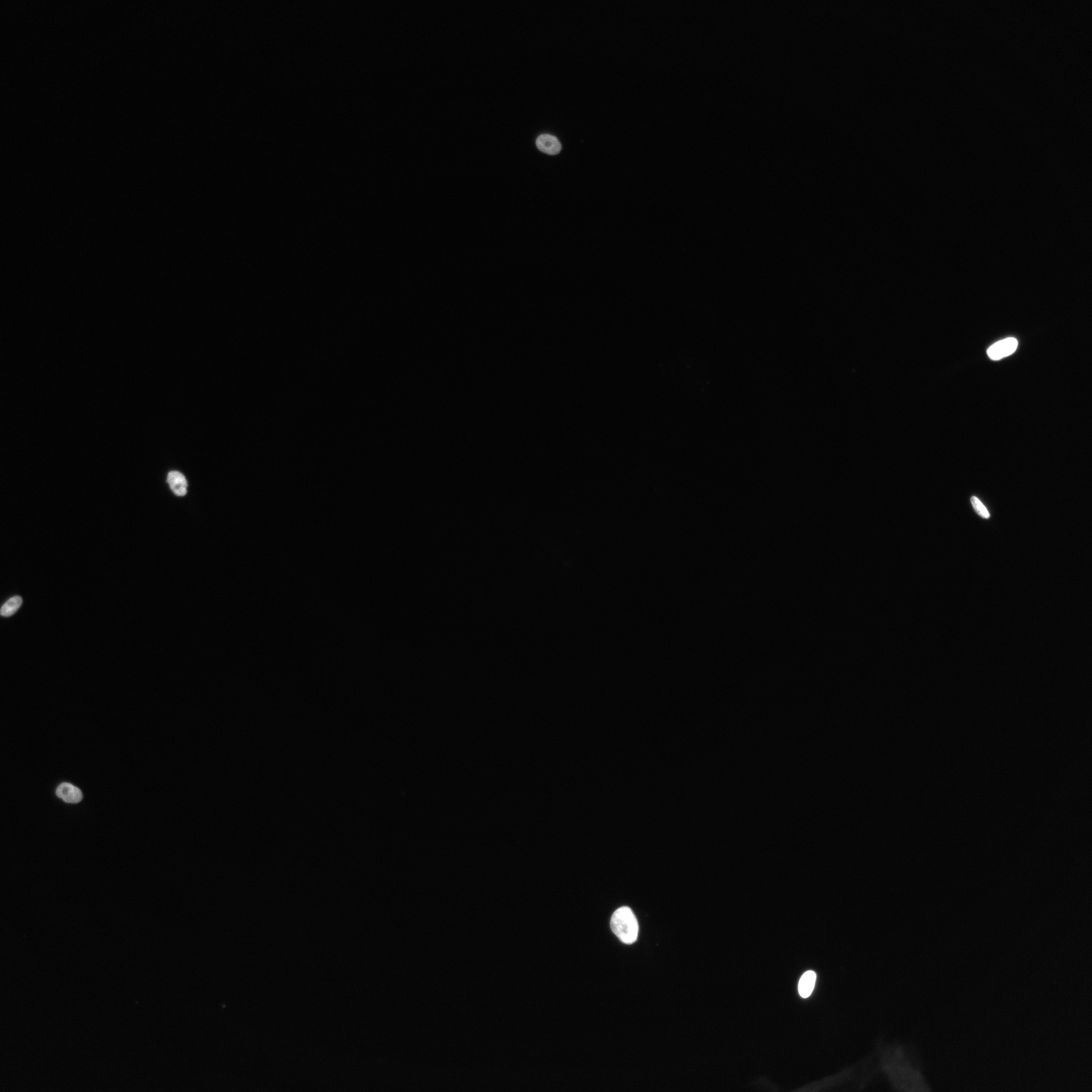

Supplement: Supplementary file 10 — Source Data for Figure 6 [file EMMM-15-e16863-s001.zip › Fig6/Fig6A/Fig6A_LG1-C-DAPI.tif]

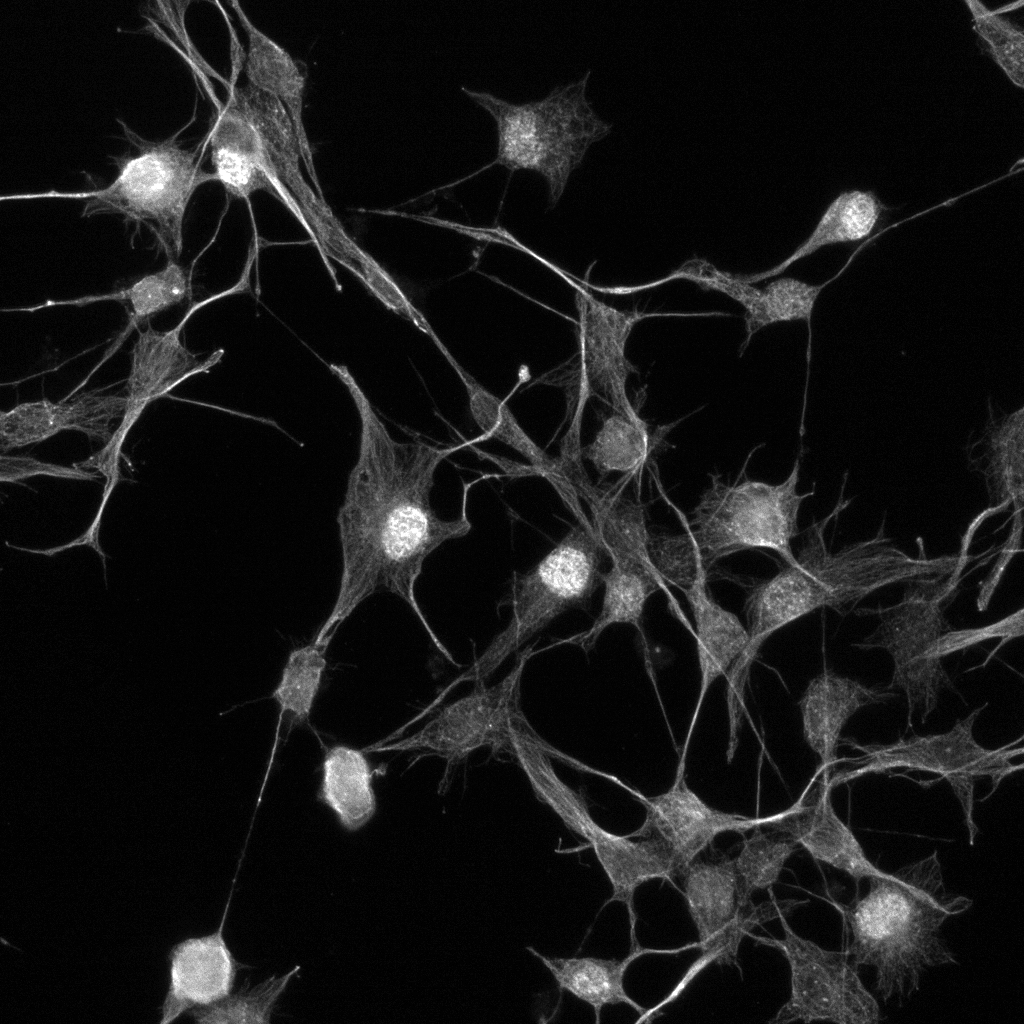

Supplement: Supplementary file 10 — Source Data for Figure 6 [file EMMM-15-e16863-s001.zip › Fig6/Fig6A/Fig6A_UPS1-TO-H2A.tif]

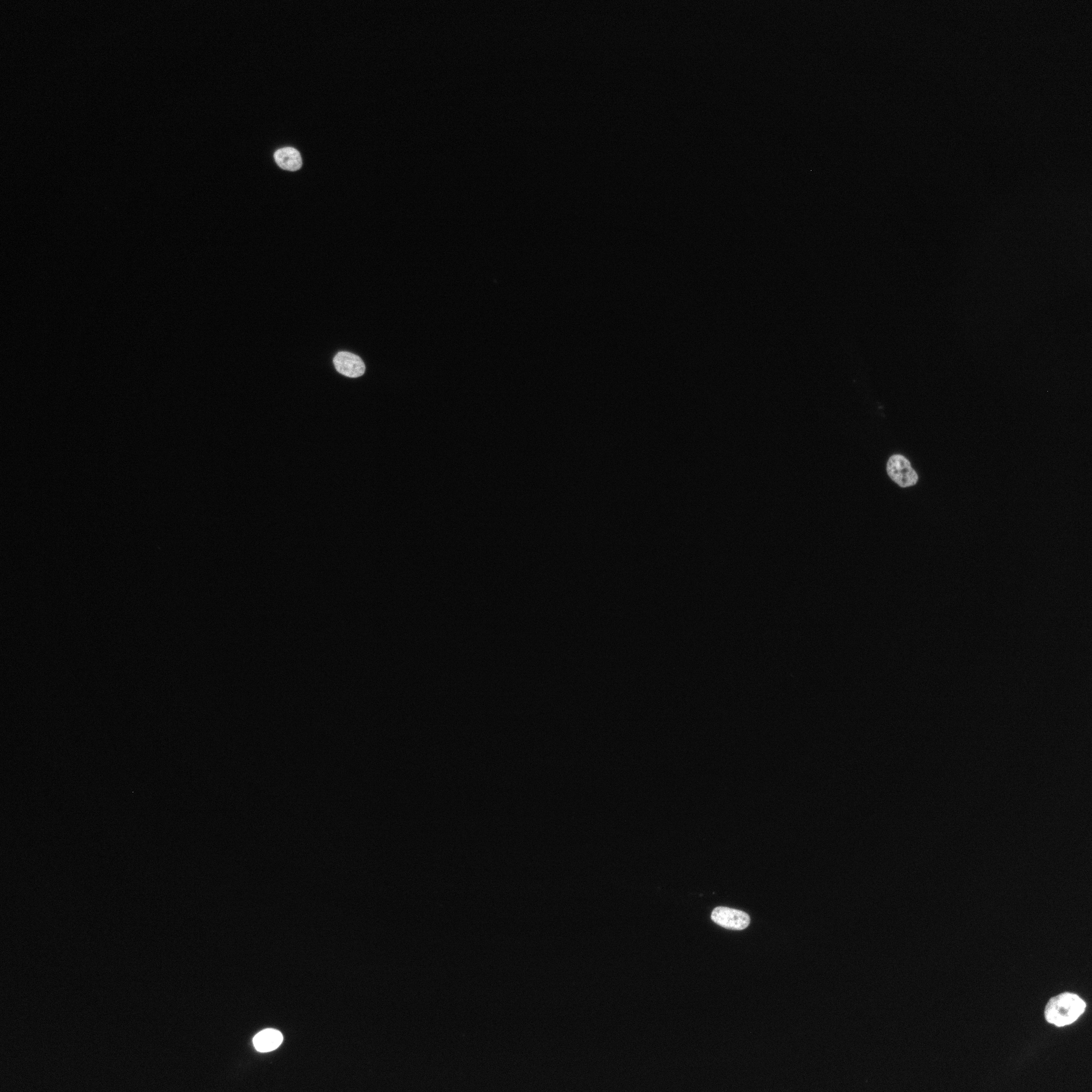

Supplement: Supplementary file 10 — Source Data for Figure 6 [file EMMM-15-e16863-s001.zip › Fig6/Fig6A/Fig6A_LG1-TO-DAPI.tif]

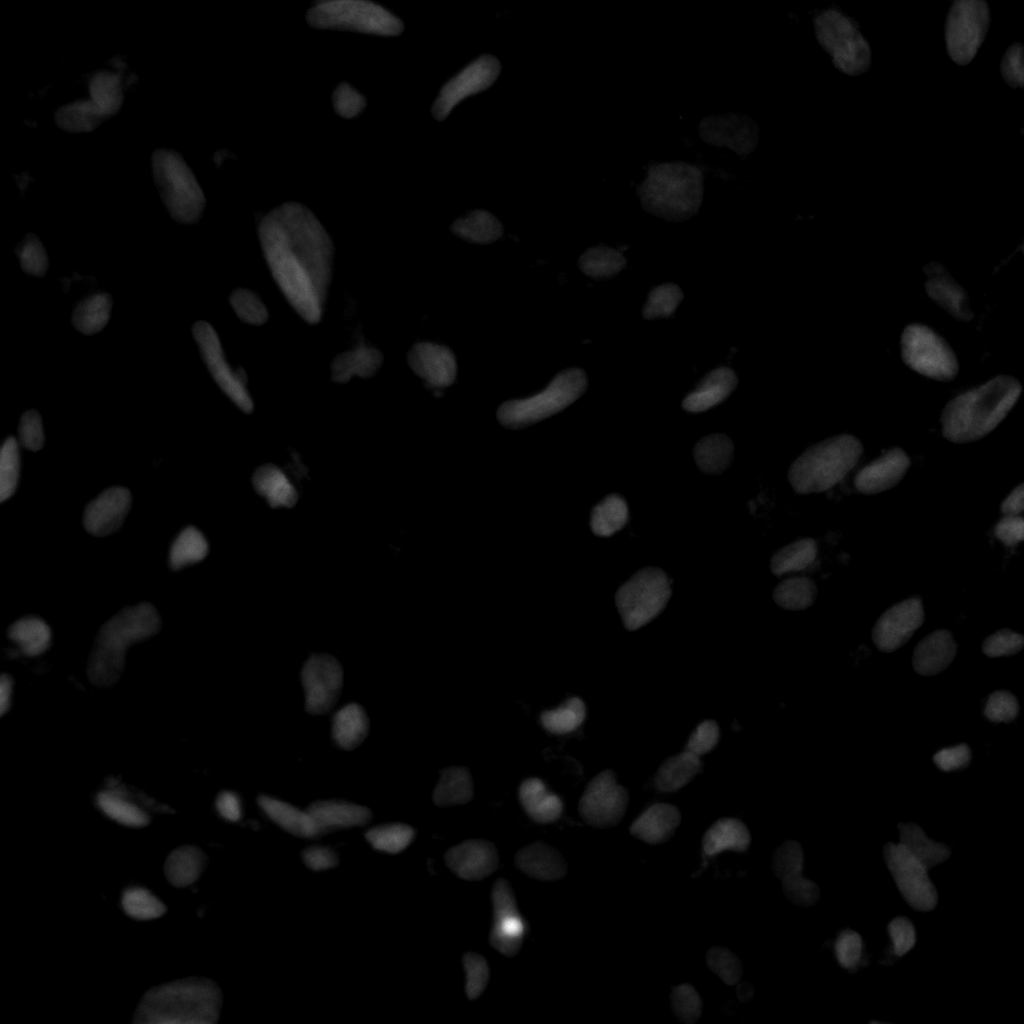

Supplement: Supplementary file 10 — Source Data for Figure 6 [file EMMM-15-e16863-s001.zip › Fig6/Fig6A/Fig6A_MFS2-TO-DAPI.tif]

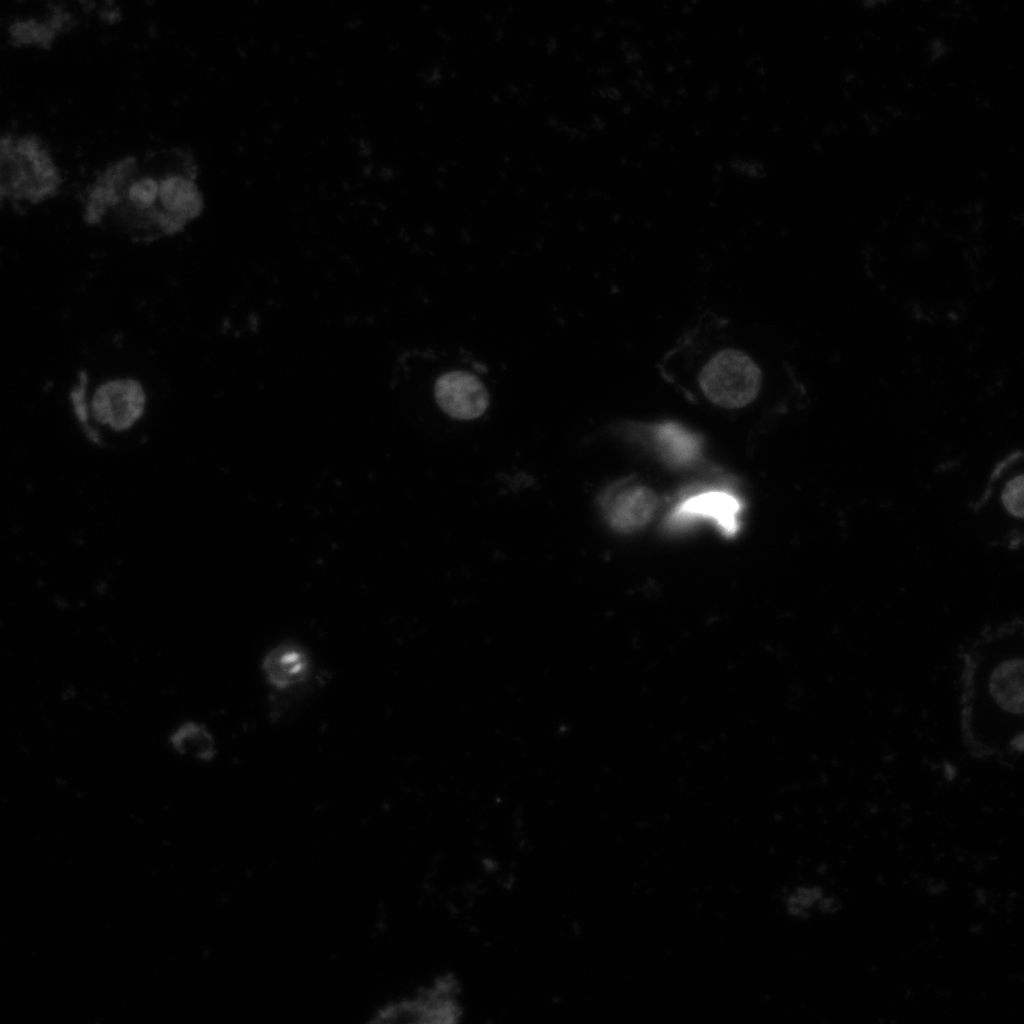

Supplement: Supplementary file 10 — Source Data for Figure 6 [file EMMM-15-e16863-s001.zip › Fig6/Fig6A/Fig6A-UWB1-TO-DAPI.tif]

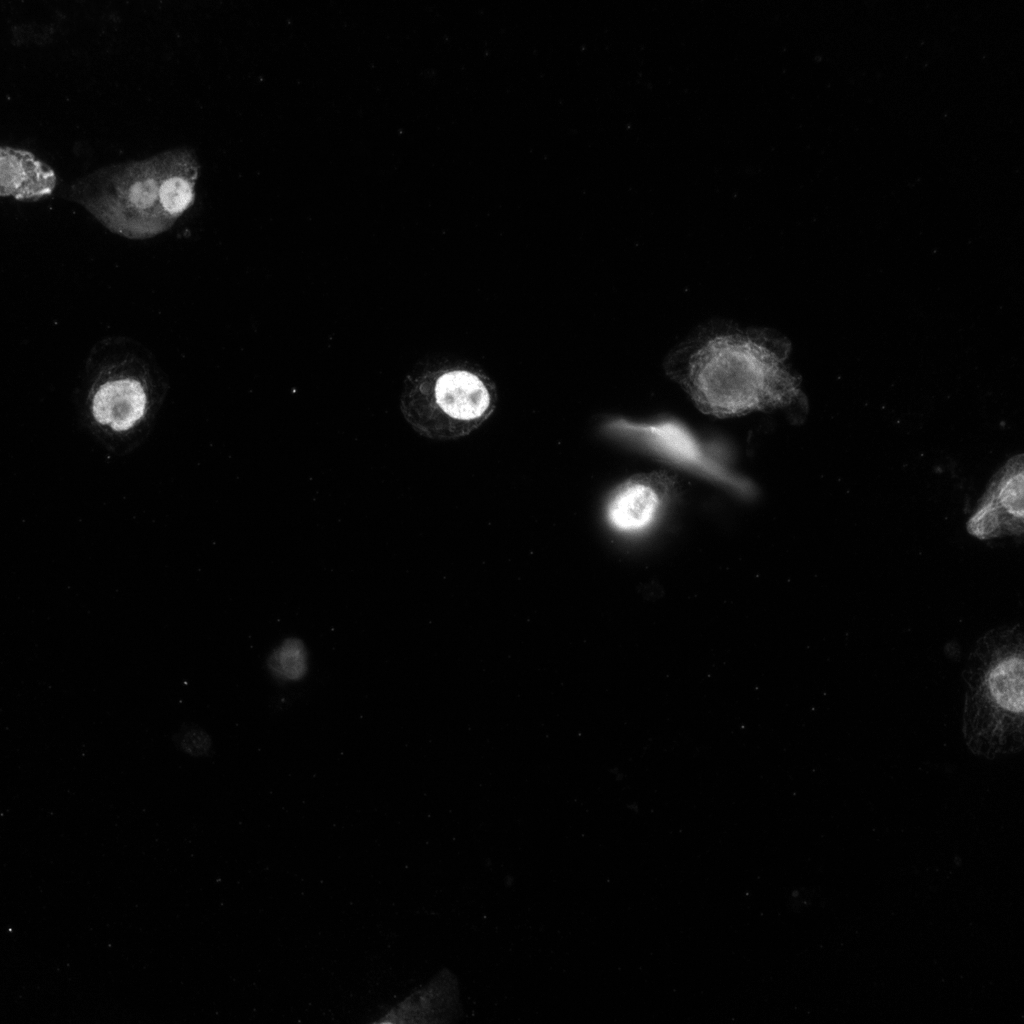

Supplement: Supplementary file 10 — Source Data for Figure 6 [file EMMM-15-e16863-s001.zip › Fig6/Fig6A/Fig6A-UWB1-TO-H2A.tif]

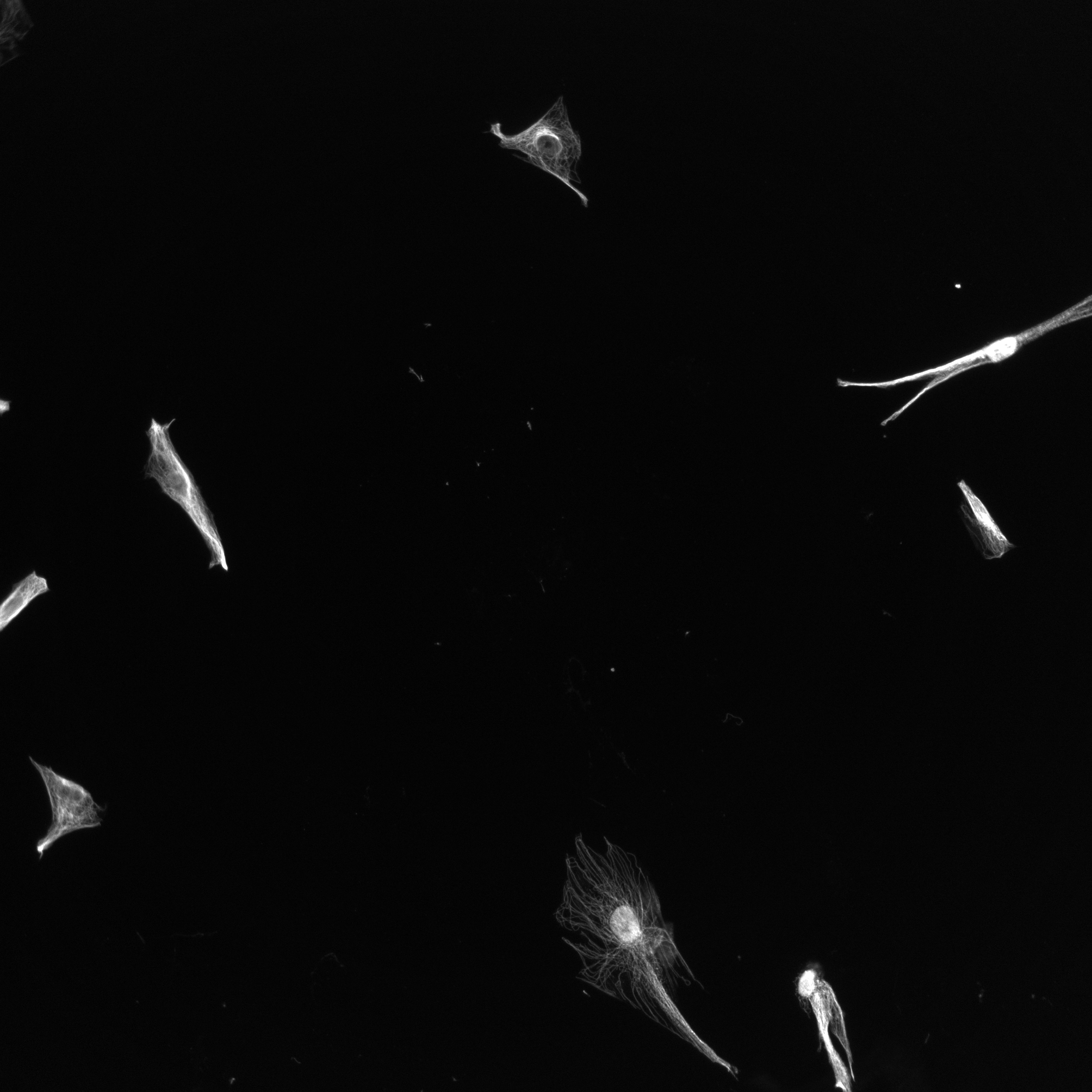

Supplement: Supplementary file 10 — Source Data for Figure 6 [file EMMM-15-e16863-s001.zip › Fig6/Fig6A/Fig6A_LG1-C-H2A.tif]

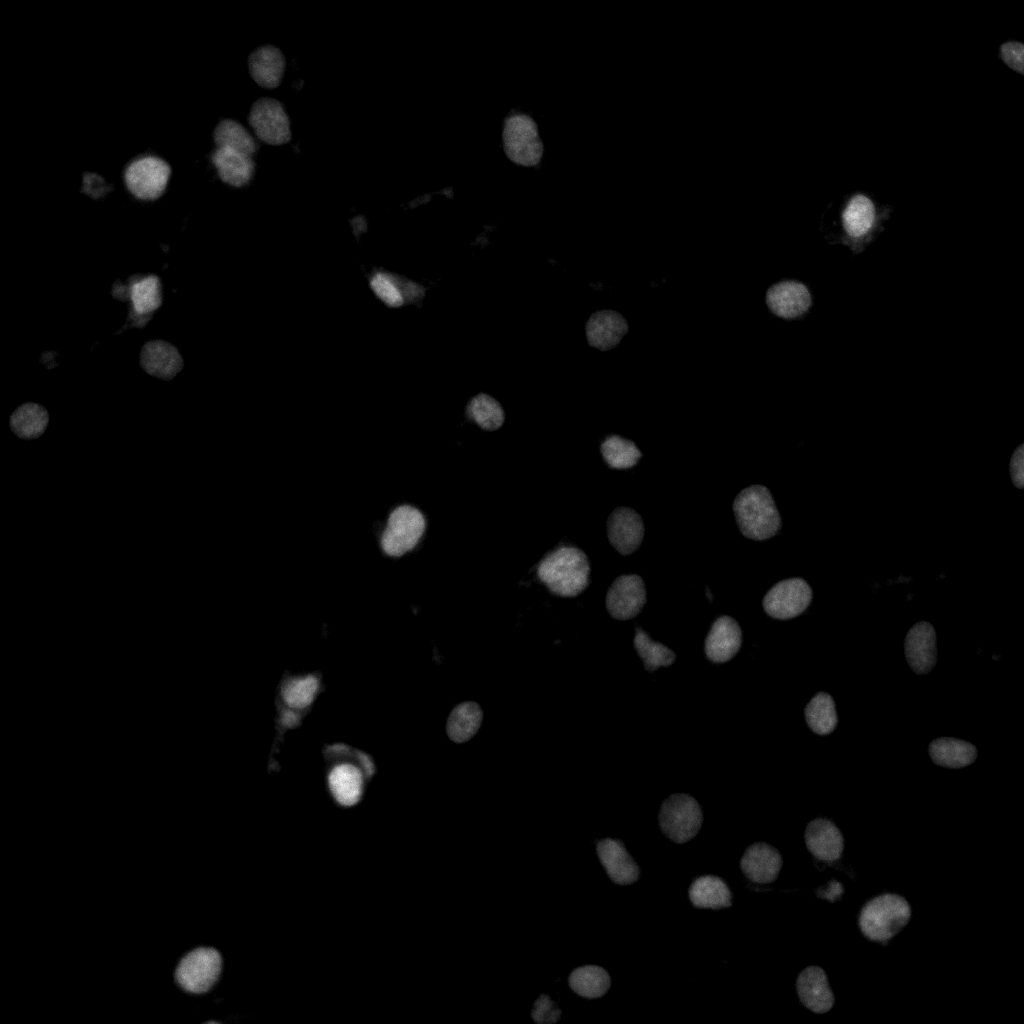

Supplement: Supplementary file 10 — Source Data for Figure 6 [file EMMM-15-e16863-s001.zip › Fig6/Fig6A/Fig6A_UPS1-TO-DAPI.tif]

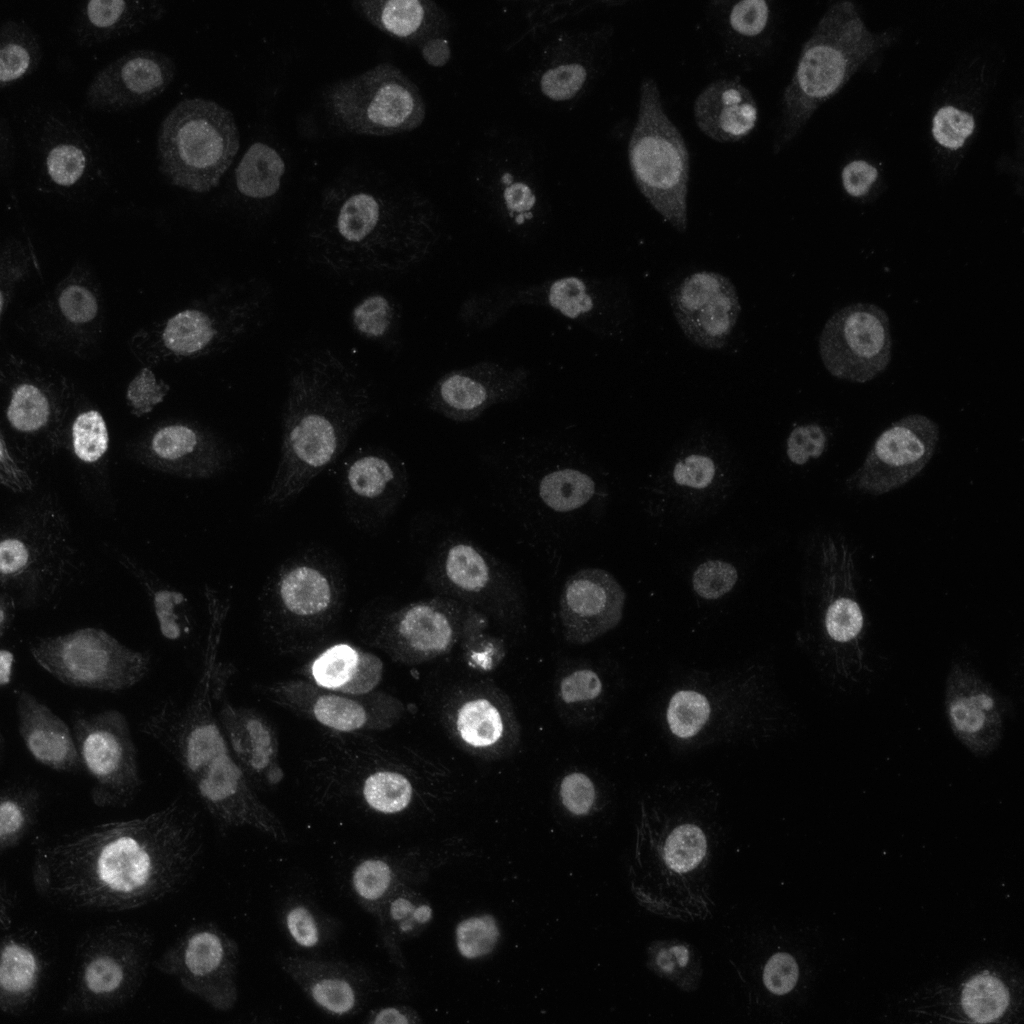

Supplement: Supplementary file 10 — Source Data for Figure 6 [file EMMM-15-e16863-s001.zip › Fig6/Fig6A/Fig6A_UWB1-C-H2A.tif]

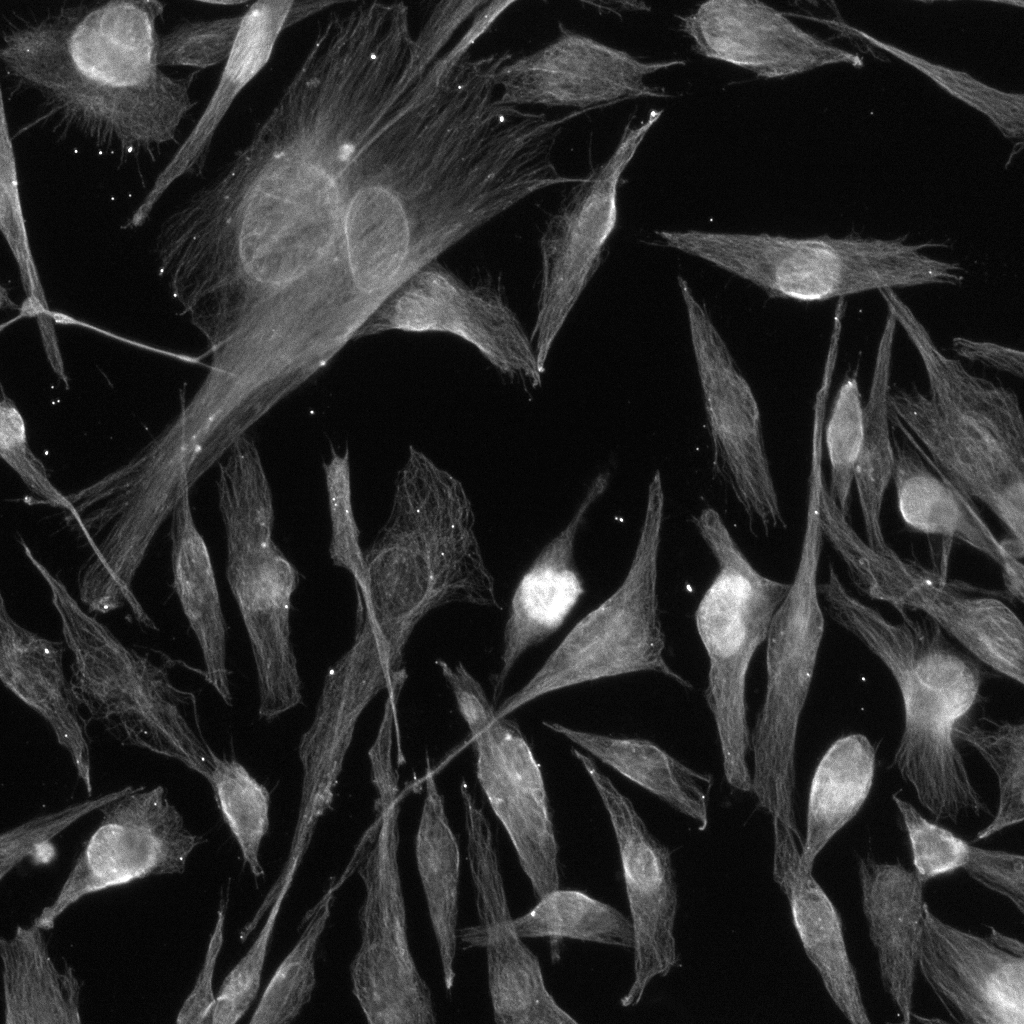

Supplement: Supplementary file 10 — Source Data for Figure 6 [file EMMM-15-e16863-s001.zip › Fig6/Fig6A/Fig6A_MFS2-C-H2A.tif]

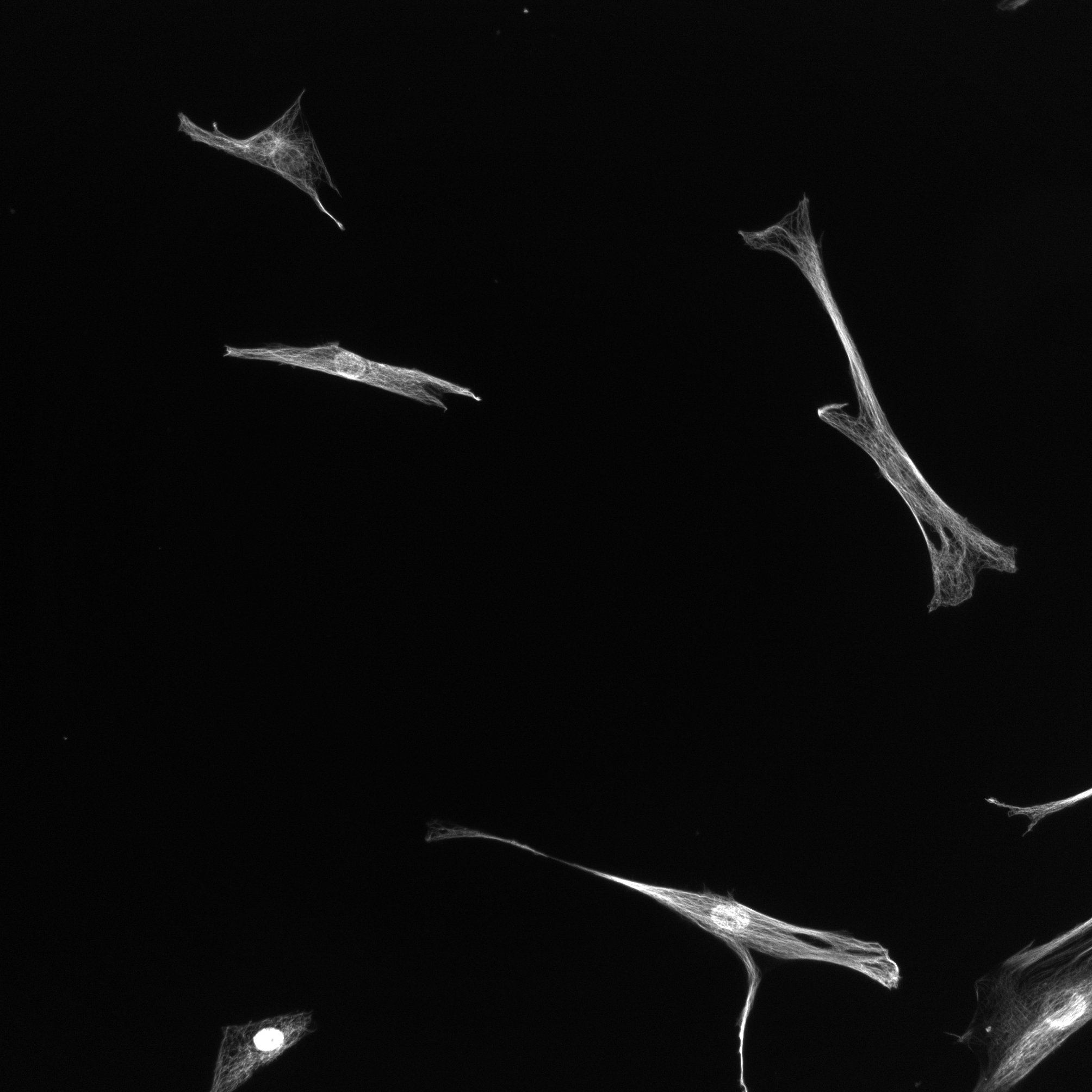

Supplement: Supplementary file 10 — Source Data for Figure 6 [file EMMM-15-e16863-s001.zip › Fig6/Fig6A/Fig6A_LG1-TO-H2A.tif]

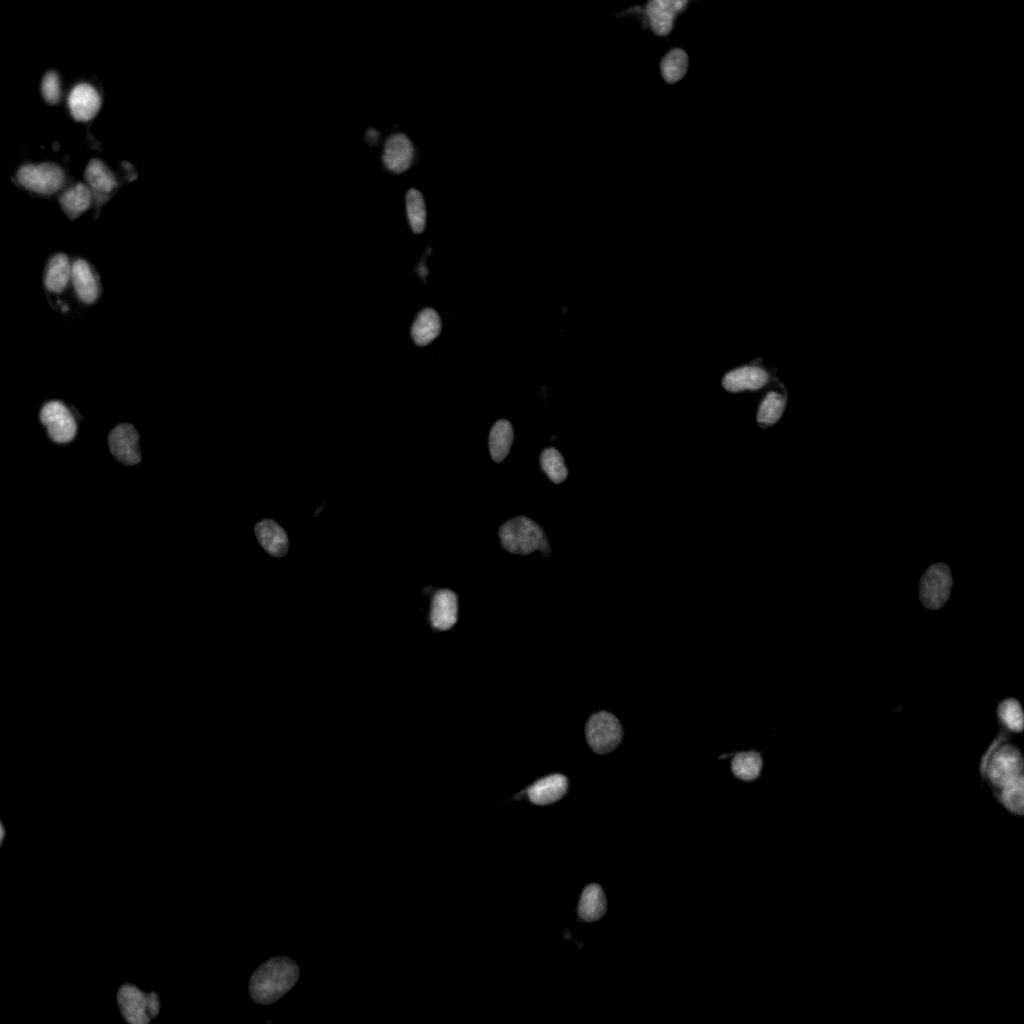

Supplement: Supplementary file 10 — Source Data for Figure 6 [file EMMM-15-e16863-s001.zip › Fig6/Fig6A/Fig6A_UPS1-C-DAPI.tif]

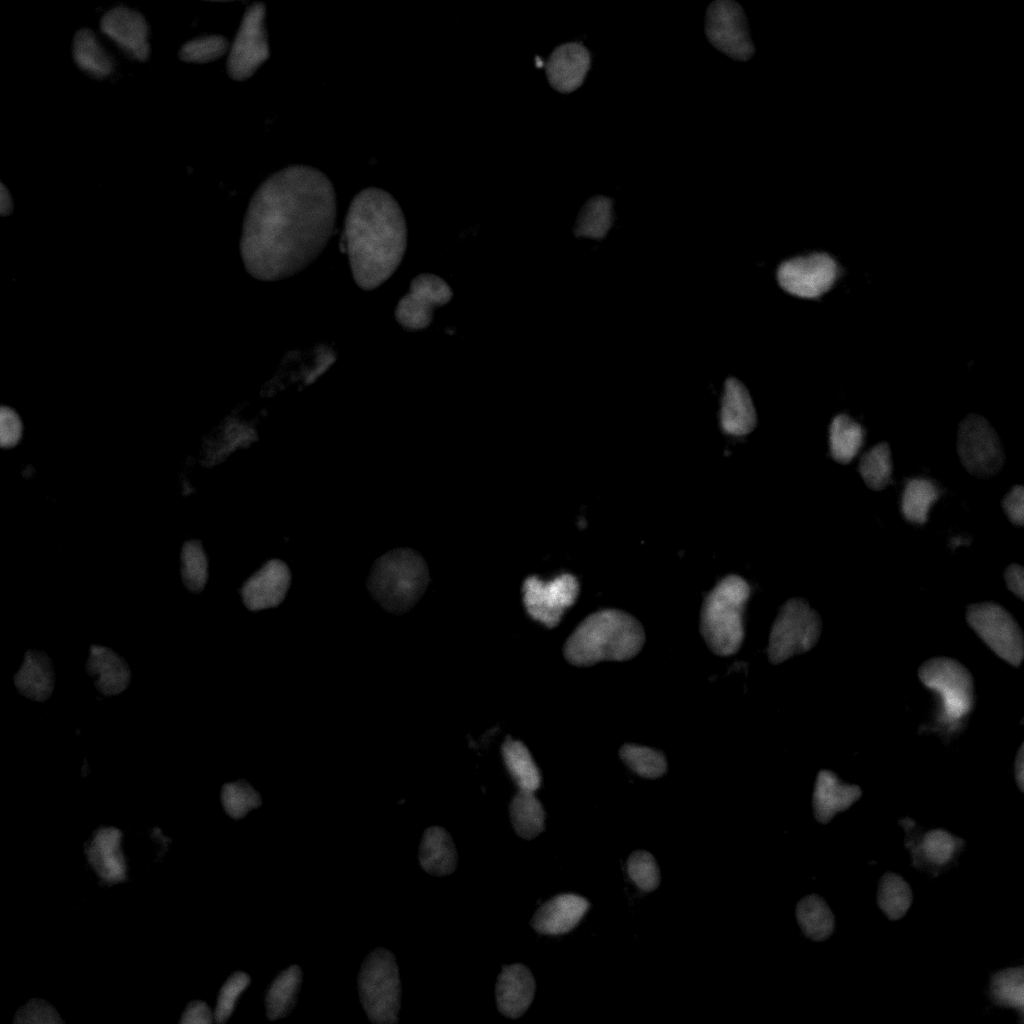

Supplement: Supplementary file 10 — Source Data for Figure 6 [file EMMM-15-e16863-s001.zip › Fig6/Fig6A/Fig6A_MFS2-C-DAPI.tif]

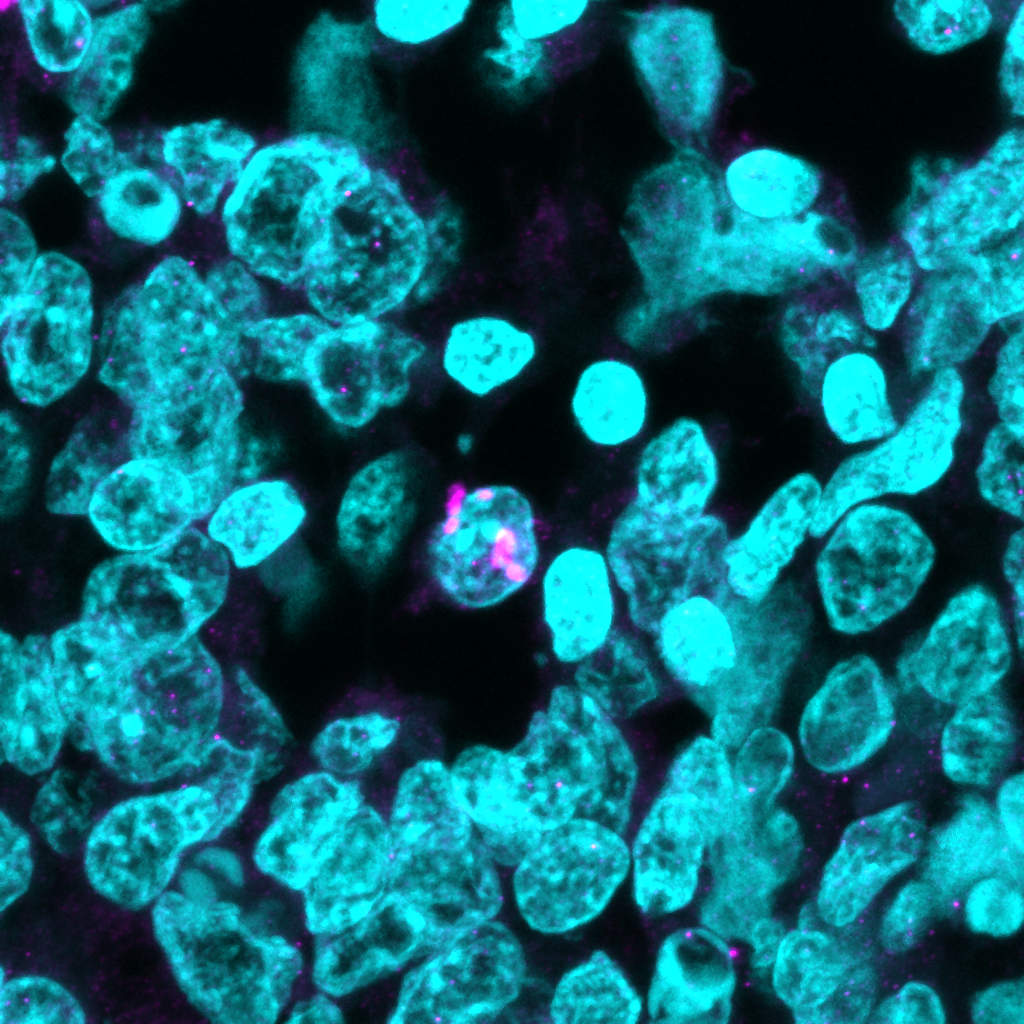

Supplement: Supplementary file 10 — Source Data for Figure 6 [file EMMM-15-e16863-s001.zip › Fig6/Fig6F/Fig6F_REA1.tif]

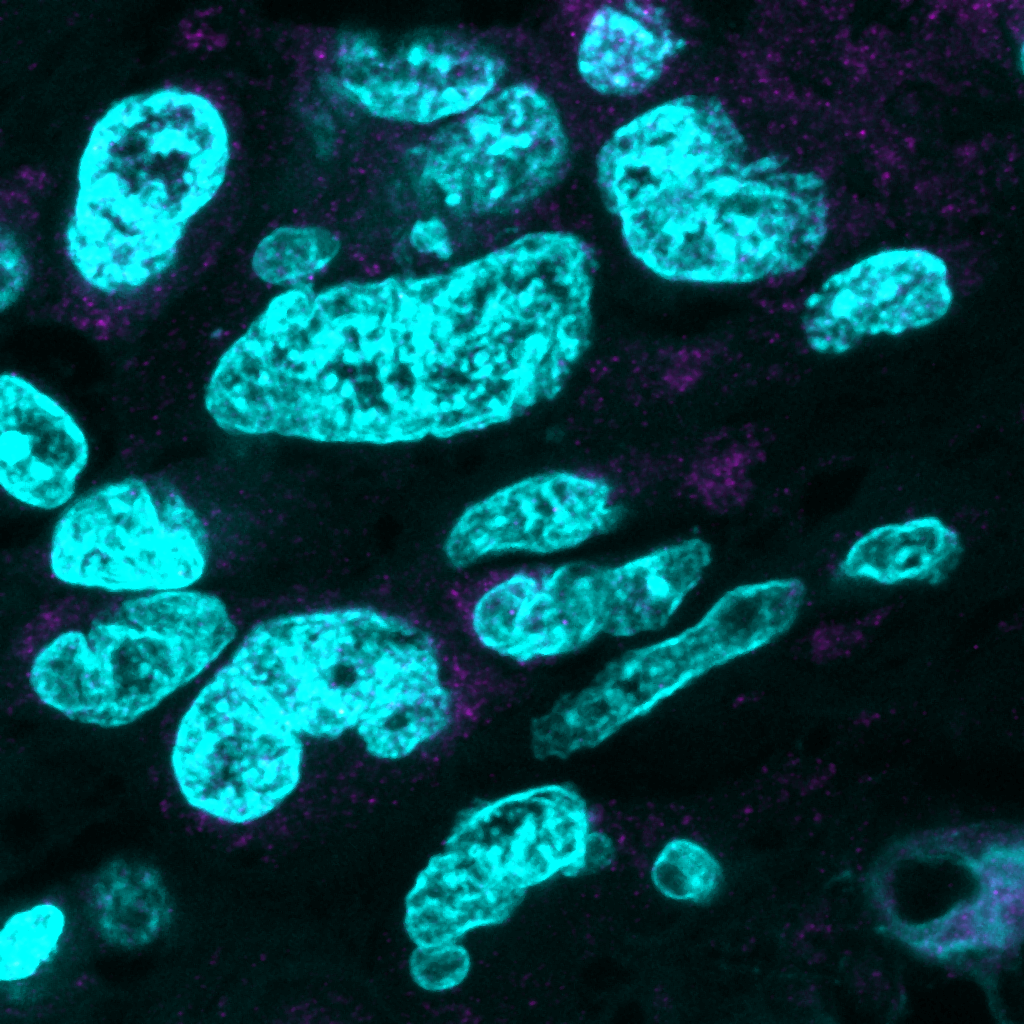

Supplement: Supplementary file 10 — Source Data for Figure 6 [file EMMM-15-e16863-s001.zip › Fig6/Fig6F/Fig6F_UPS1.tif]
