# Supplementary material for: Unravelling homologous recombination repair deficiency and therapeutic opportunities in soft tissue and bone sarcoma
Source: EMBO Mol Med. 2023 Feb 13;15(4):e16863. doi: 10.15252/emmm.202216863 (PMC10086583; doi:10.15252/emmm.202216863)
Supplement: Supplementary file 11 [file EMMM-15-e16863-s005.pdf]

EMBO Molecular Medicine

Unravelling HRDness and therapeutic opportunities in soft tissue and bone sarcoma

Lara Planas-Paz, Alicia Pliego-Mendieta, Catherine Hagedorn, Domingo Aquilera-Garcia, Martina Haberecker, Fabian Arnold, Marius Herzog, Lorenz Bankel, Roman Guggenberger, Sabrina Steiner, Yanjiang Chen, Abdullah Kahraman, Martin Zoche, Mark Rubin, Holger Moch, Christian Britschgi, and Chantal Pauli
DOI: 10.15252/emmm.202216863

Corresponding author: Chantal Pauli (chantal.pauli@usz.ch)

Review Timeline:

Submission Date:	9th Sep 22
Editorial Decision:	6th Oct 22
Revision Received:	22nd Dec 22
Editorial Decision:	17th Jan 23
Revision Received:	18th Jan 23
Accepted:	24th Jan 23

Editor: Lise Roth

Transaction Report:

(Note: With the exception of the correction of typographical or spelling errors that could be a source of ambiguity, letters and reports are not edited. Depending on transfer agreements, referee reports obtained elsewhere may or may not be included in this compilation. Referee reports are anonymous unless the Referee chooses to sign their reports.)

6th Oct 2022

Dear Prof. Pauli,

Thank you for the submission of your manuscript to EMBO Molecular Medicine. We have now received feedback from the three reviewers who agreed to evaluate your manuscript. As you will see from the reports below, the referees acknowledge the interest of the study and are overall supporting publication of your work pending appropriate revisions.

Addressing the reviewers' concerns in full will be necessary for further considering the manuscript in our journal, and acceptance of the manuscript will entail a second round of review. In agreement with the referees, please note that in vivo experiments (as mentioned by referee #2) will NOT be required for further consideration of your manuscript. EMBO Molecular Medicine encourages a single round of revision only and therefore, acceptance or rejection of the manuscript will depend on the completeness of your responses included in the next, final version of the manuscript. For this reason, and to save you from any frustrations in the end, I would strongly advise against returning an incomplete revision.

Revised manuscripts should be submitted within three months of a request for revision; they will otherwise be treated as new submissions, except under exceptional circumstances in which a short extension is obtained from the editor.

When submitting your revised manuscript, please carefully review the instructions that follow below. We perform an initial quality control of all revised manuscripts before re-review; failure to include requested items will delay the evaluation of your revision.

We require:

1) A .docx formatted version of the manuscript text (including legends for main figures, EV figures and tables). Please make sure that the changes are highlighted to be clearly visible.

2) Individual production quality figure files as .eps, .tif, .jpg (one file per figure). For guidance, download the 'Figure Guide PDF' (https://www.embopress.org/page/journal/17574684/authorguide#figureformat).

3) At EMBO Press we ask authors to provide source data for the main and EV figures. Our source data coordinator will contact you to discuss which figure panels we would need source data for and will also provide you with helpful tips on how to upload and organize the files.
Numerical data should be provided as individual .xls or .csv files (including a tab describing the data). For blots or microscopy, uncropped images should be submitted (using a zip archive if multiple images need to be supplied for one panel). Additional information on source data and instruction on how to label the files are available at
.

4) A .docx formatted letter INCLUDING the reviewers' reports and your detailed point-by-point responses to their comments. As part of the EMBO Press transparent editorial process, the point-by-point response is part of the Review Process File (RPF), which will be published alongside your paper.

5) A complete author checklist, which you can download from our author guidelines (https://www.embopress.org/page/journal/17574684/authorguide#submissionofrevisions). Please insert information in the checklist that is also reflected in the manuscript. The completed author checklist will also be part of the RPF.

6) Please note that all corresponding authors are required to supply an ORCID ID for their name upon submission of a revised manuscript.

7) It is mandatory to include a 'Data Availability' section after the Materials and Methods. Before submitting your revision, primary datasets produced in this study need to be deposited in an appropriate public database, and the accession numbers and database listed under 'Data Availability'. Please remember to provide a reviewer password if the datasets are not yet public (see https://www.embopress.org/page/journal/17574684/authorguide#dataavailability).

In case you have no data that requires deposition in a public database, please state so in this section. Note that the Data Availability Section is restricted to new primary data that are part of this study.

8) For data quantification: please specify the name of the statistical test used to generate error bars and P values, the number (n) of independent experiments (specify technical or biological replicates) underlying each data point and the test used to calculate p-values in each figure legend. The figure legends should contain a basic description of n, P and the test applied. Graphs must include a description of the bars and the error bars (s.d., s.e.m.). Please provide exact p values.

9) Our journal encourages inclusion of *data citations in the reference list* to directly cite datasets that were re-used and

obtained from public databases. Data citations in the article text are distinct from normal bibliographical citations and should directly link to the database records from which the data can be accessed. In the main text, data citations are formatted as follows: "Data ref: Smith et al, 2001" or "Data ref: NCBI Sequence Read Archive PRJNA342805, 2017". In the Reference list, data citations must be labeled with "[DATASET]". A data reference must provide the database name, accession number/identifiers and a resolvable link to the landing page from which the data can be accessed at the end of the reference. Further instructions are available at .

10) We replaced Supplementary Information with Expanded View (EV) Figures and Tables that are collapsible/expandable online. A maximum of 5 EV Figures can be typeset. EV Figures should be cited as 'Figure EV1, Figure EV2" etc... in the text and their respective legends should be included in the main text after the legends of regular figures.
- For the figures that you do NOT wish to display as Expanded View figures, they should be bundled together with their legends in a single PDF file called *Appendix*, which should start with a short Table of Content. Appendix figures should be referred to in the main text as: "Appendix Figure S1, Appendix Figure S2" etc.
- Additional Tables/Datasets should be labeled and referred to as Table EV1, Dataset EV1, etc. Legends have to be provided in a separate tab in case of .xls files. Alternatively, the legend can be supplied as a separate text file (README) and zipped together with the Table/Dataset file.
See detailed instructions here:

11) For more information: There is space at the end of each article to list relevant web links for further consultation by our readers. Could you identify some relevant ones and provide such information as well? Some examples are patient associations, relevant databases, OMIM/proteins/genes links, author's websites, etc...

12) Author contributions: CRediT has replaced the traditional author contributions section because it offers a systematic machine readable author contributions format that allows for more effective research assessment. Please remove the Authors Contributions from the manuscript and use the free text boxes beneath each contributing author's name in our system to add specific details on the author's contribution. More information is available in our guide to authors.

13) Every published paper now includes a 'Synopsis' to further enhance discoverability. Synopses are displayed on the journal webpage and are freely accessible to all readers. They include a short stand first (maximum of 300 characters, including space) as well as 2-5 one-sentences bullet points that summarizes the paper. Please write the bullet points to summarize the key NEW findings. They should be designed to be complementary to the abstract - i.e. not repeat the same text. We encourage inclusion of key acronyms and quantitative information (maximum of 30 words / bullet point). Please use the passive voice. Please attach these in a separate file or send them by email, we will incorporate them accordingly.
Please also suggest a striking image or visual abstract to illustrate your article as a PNG file 550 px wide x 300-600 px high.

14) As part of the EMBO Publications transparent editorial process initiative (see our Editorial at http://embomolmed.embopress.org/content/2/9/329), EMBO Molecular Medicine will publish online a Review Process File (RPF) to accompany accepted manuscripts.
In the event of acceptance, this file will be published in conjunction with your paper and will include the anonymous referee reports, your point-by-point response and all pertinent correspondence relating to the manuscript. Let us know whether you agree with the publication of the RPF and as here, if you want to remove or not any figures from it prior to publication.
Please note that the Authors checklist will be published at the end of the RPF.

EMBO Molecular Medicine has a "scooping protection" policy, whereby similar findings that are published by others during review or revision are not a criterion for rejection. Should you decide to submit a revised version, I do ask that you get in touch after three months if you have not completed it, to update us on the status.

I look forward to receiving your revised manuscript.

Yours sincerely,

Lise Roth

Lise Roth, PhD
Senior Editor
EMBO Molecular Medicine

***** Reviewer's comments *****

Referee #1 (Comments on Novelty/Model System for Author):

The study was carefully executed and includes an appropriate type and number of sarcoma samples. The study shows that sarcomas with homologous recombination deficiency are more sensitive to PARP1/2 and WEE1 inhibitors, and that combinations between PARPi and trabectedin, as well as WEE1 inhibitors with doxorubicin show synergistic effects. These findings support ongoing clinical trials.

Referee #1 (Remarks for Author):

In this manuscript Planas-Paz et al investigated whether soft tissue and bone sarcomas could respond to PARPi treatment based on their homologous recombination deficiency (HRD). Many sarcomas have a high HRD score with mutations in FANC and BRCA2 and chromosomal instability in the form of chromosome duplications and losses. These patterns were found across different samples: TCGA cohorts, five myxofibrosarcoma patients, 21 angiosarcomas and 282 soft tissue and bone sarcoma patients. High HRD score is associated with increased expression of DNA repair genes and genes regulated by Myc and E2F transcription factors. The authors established patient-derived sarcoma cell models with high or low HRD score and tested their sensitivity to PARPi. HRD high samples showed impaired RAD51 foci formation, in accordance with defective homologous recombination. HRD high samples were more sensitive to PARP and WEE1 inhibitors compared to HRD low samples, which does not seem to be the case for oxaliplatin, doxorubicin and trabectedin. PARPi combined with trabectedin as well as WEE1i combined with doxorubicin showed a synergistic effect. A patient with extra-uterine leiomyosarcoma (LMS) showed partial remission after treatment with PARPi combined with trabectedin, indicating potential benefits of this combinatorial treatment for sarcomas. This study was carefully executed, well written, and reports medically relevant findings.
Major critique:
1) Fig. 5 should include an equal number of HRD high and low samples for proper comparison.
Minor critique:
1) line 207-209 - syntax issue
2) Figure legends: please indicate how many samples were analysed and their source in each figure or figure panel
3) Fig. 5a-c: why weren't all sarcoma models analysed as in panels d-l?

Referee #2 (Comments on Novelty/Model System for Author):

Well-executed, well-written. Elegant design. Clinically highly relevant. I strongly suggest acceptance of this manuscript and publication with high priority.

Referee #2 (Remarks for Author):

In this elegant study, Planas-Paz and Mendieta et al use the TCGA sarcoma dataset to demonstrate homologous recombination repair deficiency (HRRD) in sarcomas. They go on to validate their findings in a cohort of 5 myxoid fibrocarcomas and in another cohort of 21 angiosarcomas. They use PDX cell cultures established from 4 HRRDhigh and 2 HRRDlow sarcomas to show higher in vitro efficacy of PARP inhibitors (+/- trabectedin) and WEE1 inhibitors (+/- doxorubicin), and they evaluate formation of RAD51 nuclear foci as a functional biomarker of HRR in sarcoma tissue. Finally, they report prolonged stable disease over a period of 18 months in a patient with refractory leiomyosarcoma treated with trabectedin and olaparib. This is a very nice and clinically highly relevant study. I strongly suggest acceptance of this manuscript and publication with high priority.

It would be great to see some data on the effects of olaparib +/- trabectedin and adavosertib (+/- doxorubicin) on patient-derived xenografts in mice. However, I would like to emphasize that the body of evidence is convincing without these experiments, and I feel strongly that the manuscript should be accepted without additional mouse studies.

I have a few comments/ suggestions for improvements:

- Title and introduction, line 100: The authors discuss HRDness. What is HRDness? And how does it compare to BRCAness? There are all these catchy terms flying around, which are barely defined and very hard to handle in practical life.
- It is very striking that 22% of sarcomas carry BRCA2 alterations. BRCA2 germline variants also turn out to be relatively frequent in individuals diagnosed with sarcomas. I would appreciate further discussion.
- Results, line 54-61: The manuscript would benefit from a bit more detail about how these rather important threshold values were calculated.
- I appreciate the case report at the end of the manuscript. Why did the authors not apply the SARC-HRD score introduced in the manuscript and perform RAD51 staining? It would be very nice to see these data.

Minor issues:
- Introduction, line 80-84: Pazopanib is not necessarily second-line for all sarcomas; please rephrase and provide more accuracy.
- Introduction, line 60: "PARP is a specific vulnerability in cancer cells that cannot adequately repair DSB DNA damage...". Pleas rephrase and provide more clarity.
- Introduction, line 75: "soft tissue" instead of "soft tissues"

Referee #3 (Comments on Novelty/Model System for Author):

In 2020, Li et al (PMID: 32863940) have previously explored BRCAness and PARP inhibitor sensitivity in 22 STS tumors and compared their findings to analysis of the TCGA data, similar to this study. Their work also included synergy studies with TMZ; however, in that case, so this study is not novel per se. It represents validation of already established data from Li et al publication with new cohort of patients, including angiosarcoma. Importantly, the authors add a new cohort of 282 STS patients where 409 genes were analyzed via targeted panel genome sequencing. Interestingly, some angiosarcoma and Ewing's sarcoma tumors also exhibit high LOH. I appreciated the inclusion of two HRD-low patient derived cell models from BCOR-rearranged sarcoma and unclassified low-grade sarcoma.

Referee #3 (Remarks for Author):

Sarcoma remains a treatment refractory cancer and thus there is an unmet clinical need to identify effective therapeutic modalities for these rare cancers of heterogeneous origins. A hallmark feature of many soft tissue and bone sarcomas is chromosomal instability likely due to defects in homologous recombination repair mechanisms. While this has been studied in other cancers, the precise extent of HRDness and how that correlates to sensitivity of these cells to agents like PARP or WEE1 inhibitors is not well established. HRD extends beyond BRCA1/2 deficiencies, which are well established markers of homologous recombination repair deficits in cancer. Other genes involved in this include ATM, ATR, CHK1, CHK2, PALB2, RAD51 and FANC family. Since deficits in homologous recombination repair may sensitize cells via synthetic lethal mechanisms to above mentioned agents, it is important to accurately predict or profile the HRD signature that may be difference in cancers of diverse origins. In this manuscript, Planas-Plaz and team interrogated these ideas using patient samples and clinical cohorts using a multi-omics approach.

They used pre-existing TCGA-SARC cohort data (247 cases) and TARGET-OS (69 cases) to explore loss of heterozygosity (LOH), large scale transitions (LST) and telomeric allelic imbalance (TAI). They validated their approach using high-grade serous ovarian cancer, triple negative breast cancer and colorectal cancer as comparison, since HRD parameters are well established in these entities. Authors found varying HRD scores and instability signatures between the different sarcoma subtypes. UPS, OS, MFS and ULMS had higher HRD parameter scores compared to MPNST, LMS, DDLPS as well as the fusion driven synovial sarcoma. I am not sure if these differences are statistically or biologically significant given large error bar/deviance. Figure 2 is incrementally looking at chromosomal gains and losses. Essentially this figure corroborates that HRD high sarcomas had high CIN. Some anecdotal interesting observations are - DDLPS had high Ch. 12 amplification and CIN.

A notable finding is that the total number of alterations in HRD genes per sarcoma case shows a bimodal distribution. Based on these data, the HRD cut-off value was established to be 32 for authors here for the sarcoma cohort, independent of the subtype. I would like to have understood exactly how the authors used the biomodal distribution data to then compute the HRR specific CIN score ... it was not apparent to me how that was done. Authors then assessed CIN in HRD-high versus HRD-low STS and bone sarcoma cases. Positive controls were serous ovarian cancer and BRAC1/2 aberrant triple negative breast cancer. CRC was used as a negative control. As expected, HRD high STS and bone sarcoma had high aneuploidy and chromosomal duplication events. Authors also interrogated 21 angiosarcoma cases and found some to harbor HRD.

It was interesting and not unexpected that the authors found high TMB in sarcoma patients whose tumors/cancer arose in sun-exposed location and thus exhibited a UV-induced mutation pattern.

In 2020, Li et al (PMID: 32863940) have previously explored BRCAness and PARP inhibitor sensitivity in 22 STS tumors and compared their findings to analysis of the TCGA data, similar to this study. Their work also included synergy studies with TMZ; however, in that case, so this study is not novel per se. It represents validation of already established data from Li et al publication with new cohort of patients, including angiosarcoma. Importantly, the authors add a new cohort of 282 STS patients where 409 genes were analyzed via targeted panel genome sequencing. Interestingly, some angiosarcoma and Ewing's sarcoma tumors also exhibit high LOH. I appreciated the inclusion of two HRD-low patient derived cell models from BCOR-rearranged sarcoma and unclassified low-grade sarcoma.

Functional experiments were performed on the following patient-derived models: 4 MFS, 1 UPS cell line.
I find the study to be comprehensive, well written with appropriate positive and negative controls included. Although the findings are particularly or entirely novel, they represent an important validation cohort and advancement of understanding profile of HRD in sarcoma and correlated sensitivity to combination therapy employing PARPi and DNA damaging agents. The proof of concept

in an index patient case is also compelling for motivating future clinical trials with appropriate measurement metrics built in that will ultimately guide employment of these agents to treat sarcoma in patients selected based on biomarkers or HRD.

Minor Comments:
- The acronym CIN appears in Introduction without explanation. It will be beneficial for new reader or broader audience if you briefly elaborated on that.

***** Reviewer's comments *****

Referee #1 (Comments on Novelty/Model System for Author):
The study was carefully executed and includes an appropriate type and number of sarcoma samples. The study shows that sarcomas with homologous recombination deficiency are more sensitive to PARP1/2 and WEE1 inhibitors, and that combinations between PARPi and trabectedin, as well as WEE1 inhibitors with doxorubicin show synergistic effects. These findings support ongoing clinical trials.

Referee #1 (Remarks for Author):
In this manuscript Planas-Paz et al investigated whether soft tissue and bone sarcomas could respond to PARPi treatment based on their homologous recombination deficiency (HRD). Many sarcomas have a high HRD score with mutations in FANC and BRCA2 and chromosomal instability in the form of chromosome duplications and losses. These patterns were found across different samples: TCGA cohorts, five myxofibrosarcoma patients, 21 angiosarcomas and 282 soft tissue and bone sarcoma patients. High HRD score is associated with increased expression of DNA repair genes and genes regulated by Myc and E2F transcription factors. The authors established patient-derived sarcoma cell models with high or low HRD score and tested their sensitivity to PARPi. HRD high samples showed impaired RAD51 foci formation, in accordance with defective homologous recombination. HRD high samples were more sensitive to PARP and WEE1 inhibitors compared to HRD low samples, which does not seem to be the case for oxaliplatin, doxorubicin and trabectedin. PARPi combined with trabectedin as well as WEE1i combined with doxorubicin showed a synergistic effect. A patient with extra-uterine leiomyosarcoma (LMS) showed partial remission after treatment with PARPi combined with trabectedin, indicating potential benefits of this combinatorial treatment for sarcomas. This study was carefully executed, well written, and reports medically relevant findings.

Referee #1 Major critique
1) Fig. 5 should include an equal number of HRD high and low samples for proper comparison.
We now included three more sarcoma cell models (solitary fibrous tumour, extraskeletal myxoid chondrosarcoma and CIC-DUX fusion driven sarcoma) that we established in-house from patient-derived tissue samples. Based on our current understanding of HRD*ness* in sarcoma, we predicted all three cell models to be characterized by low HRD scores. We subjected the cells to whole-genome sequencing and RNA-sequencing as well as performed functional drug assays (Fig. 5A-L). All new sarcoma cell models exhibited low HRD scores and decreased expression of HRR pathway genes compared with HRD^{high}. Accordingly, HRD^{low} cell models did not respond to PARP or WEE1 inhibition.

Referee #1 Minor critique
1) line 207-209 - syntax issue
We adapted it.
2) Figure legends: please indicate how many samples were analysed and their source in each figure or figure panel

We have now updated all figure legends with detailed information on sample number and datasets analysed.
3) Fig. 5a-c: why weren't all sarcoma models analysed as in panels d-l?
We now performed whole-genome sequencing and RNA-sequencing of all sarcoma cell models including three new ones (Fig. 5A-D).

Referee #2 (Comments on Novelty/Model System for Author):
Well-executed, well-written. Elegant design. Clinically highly relevant. I strongly suggest acceptance of this manuscript and publication with high priority.

Referee #2 (Remarks for Author):
In this elegant study, Planas-Paz and Mendieta et al use the TCGA sarcoma dataset to demonstrate homologous recombination repair deficiency (HRRD) in sarcomas. They go on to validate their findings in a cohort of 5 myxoid fibrocarcomas and in another cohort of 21 angiosarcomas. They use PDX cell cultures established from 4 HRRDhigh and 2 HRRDlow sarcomas to show higher in vitro efficacy of PARP inhibitors (+/- trabectedin) and WEE1 inhibitors (+/- doxorubicin), and they evaluate formation of RAD51 nuclear foci as a functional biomarker of HRR in sarcoma tissue. Finally, they report prolonged stable disease over a period of 18 months in a patient with refractory leiomyosarcoma treated with trabectedin and olaparib. This is a very nice and clinically highly relevant study. I strongly suggest acceptance of this manuscript and publication with high priority.<br>It would be great to see some data on the effects of olaparib +/- trabectedin and adavosertib (+/- doxorubicin) on patient-derived xenografts in mice. However, I would like to emphasize that the body of evidence is convincing without these experiments, and I feel strongly that the manuscript should be accepted without additional mouse studies.

Referee #2 I have a few comments/ suggestions for improvements:
1) Title and introduction, line 100: The authors discuss HRDness. What is HRDness? And how does it compare to BRCAness? There are all these catchy terms flying around, which are barely defined and very hard to handle in practical life.
We believe the term BRCAness does neither reflect nor encompass the defects in homologous recombination repair (HRR) observed in sarcoma. The concept of BRCAness is by name related to breast cancer susceptibility, and by definition BRCAness is an HRR deficiency, mimicking *BRCA1* or *BRCA2* loss, which confers significantly elevated risks of breast, ovarian and other cancers (Lord & Ashworth. 2016. Nature Reviews Cancer 16:110-20, Byrum *et al*. 2019. Trends in Cell Biology 9:740-51, Liu *et al*. 2020. Scientific Reports 10:21173).<br>We have addressed the differences in nomenclature in the introduction (lines 56-59) and have spelled out the term HRD in the title.
2) It is very striking that 22% of sarcomas carry BRCA2 alterations. BRCA2 germline variants also turn out to be relatively frequent in individuals diagnosed with sarcomas. I would appreciate further discussion.

We agree with the reviewer that BRCA2 alterations are prevalent in all our sarcoma datasets. Notably, reversion mutations in *BRCA2* conferring PARPi resistance have been reported in other tumour types, which underscores the need to pre-emptively look for them. We advocate for the use of targeted genomic panels that are feasible to implement in clinical settings and will aid clinical decision-making.<br>We have now incorporated a few sentences to discuss this fact (lines 467-469).
3) Results, line 54-61: The manuscript would benefit from a bit more detail about how these rather important threshold values were calculated.
We incorporated a more detailed explanation in the methods and main text. In summary, we described how we statistically inferred optimal HRD score and LOH cut-off values from HRR-CIN data from the TCGA-SARC cohort:<br>"As molecular alterations in HRR genes in soft tissue sarcoma follow a bimodal distribution (two peaks), we applied a finite mixture model (probabilistic model for representing the presence of subpopulations within an overall population) to separate both subpopulations into HRR-CIN^{high} and HRR-CIN^{low}. The confidence interval from the cut-off was computed by Monte Carlo simulations and resulted in a cut-off value of 37. For each sample, an HRR-CIN status can be defined and associated with an HRD score. In order to infer a cut-off for the HRD score (which is one of the most clinically used biomarkers together with LOH), we plotted a receiver operating characteristic (ROC) curve with HRD score data considering the predicted HRR-CIN status. An ROC curve illustrates the diagnostic ability of a binary classifier as the classification thresholds are varied. ROC curves show the trade-off between sensitivity (x axis: true positive rate) and specificity (y axis: false positive rate, 1-specificity). While a random classifier gives curves along the diagonal, classifiers that give curves closer to the top-left corner indicate better performance. We next applied the Youden index to select the optimum HRD score cut-off point as it summarizes the ROC curve and shows the maximum potential effectiveness of a biomarker (in this case, the HRD score)."
4) I appreciate the case report at the end of the manuscript. Why did the authors not apply the SARC-HRD score introduced in the manuscript and perform RAD51 staining? It would be very nice to see these data.
To address this reviewer's point, (1) we showed absence of nuclear RAD51 staining in a gastric metastasis from this patient, further corroborating the validity of this diagnostic biomarker (Fig 6J); and (2) we performed whole-genome sequencing and obtained an HRD score of 83, a total of 22% of genomic alterations, and gains and losses in several HRR genes (Fig. 6I, 6K).<br>Due to lack of fresh frozen tissue from this patient, we performed RNA-seq with FFPE-derived material. However, we obtained only little amount of RNA with an unexpected pattern in nucleotide content. The sample did not pass our quality control requirements and prevented us from further analysis of the SARC-HRD gene expression score.

Referee #2 Minor comments
1) Introduction, line 80-84: Pazopanib is not necessarily second-line for all sarcomas; please rephrase and provide more accuracy.
We addressed this relevant comment by specifying that second-line treatment is increasingly tailored towards distinct histological subtypes and encompasses the use of multiple drugs (lines 82-85). We additionally cited two references (line 85).

2) Introduction, line 60: "PARP is a specific vulnerability in cancer cells that cannot adequately repair DSB DNA damage...". Pleas rephrase and provide more clarity.
We clarified the sentence as follows: "PARPi trap PARP1/DNA nucleoprotein complexes and stall the advancement of the replication fork. Cancer cells that cannot adequately repair DSB DNA damage via the high-fidelity HRR pathway alternatively use more error-prone DNA repair mechanisms." (lines 62-64)
3) Introduction, line 75: "soft tissue" instead of "soft tissues"
We corrected this; it can be now found in line 77, highlighted in yellow.

Referee #3 (Comments on Novelty/Model System for Author):
In 2020, Li et al (PMID: 32863940) have previously explored BRCAness and PARP inhibitor sensitivity in 22 STS tumors and compared their findings to analysis of the TCGA data, similar to this study. Their work also included synergy studies with TMZ; however, in that case, so this study is not novel per se. It represents validation of already established data from Li et al publication with new cohort of patients, including angiosarcoma. Importantly, the authors add a new cohort of 282 STS patients where 409 genes were analyzed via targeted panel genome sequencing. Interestingly, some angiosarcoma and Ewing's sarcoma tumors also exhibit high LOH. I appreciated the inclusion of two HRD-low patient derived cell models from BCOR-rearranged sarcoma and unclassified low-grade sarcoma.

Referee #3 (Remarks for Author):
Sarcoma remains a treatment refractory cancer and thus there is an unmet clinical need to identify effective therapeutic modalities for these rare cancers of heterogeneous origins. A hallmark feature of many soft tissue and bone sarcomas is chromosomal instability likely due to defects in homologous recombination repair mechanisms. While this has been studied in other cancers, the precise extent of HRDness and how that correlates to sensitivity of these cells to agents like PARP or WEE1 inhibitors is not well established. HRD extends beyond BRCA1/2 deficiencies, which are well established markers of homologous recombination repair deficits in cancer. Other genes involved in this include ATM, ATR, CHK1, CHK2, PALB2, RAD51 and FANC family. Since deficits in homologous recombination repair may sensitize cells via synthetic lethal mechanisms to above mentioned agents, it is important to accurately predict or profile the HRD signature that may be difference in cancers of diverse origins. In this manuscript, Planas-Plaz and team interrogated these ideas using patient samples and clinical cohorts using a multi-omics approach.

Referee #3
1) They used pre-existing TCGA-SARC cohort data (247 cases) and TARGET-OS (69 cases) to explore loss of heterozygosity (LOH), large scale transitions (LST) and telomeric allelic imbalance (TAI). They validated their approach using high-grade serous ovarian cancer, triple negative breast cancer and colorectal cancer as comparison, since HRD parameters are well established in these entities. Authors found varying HRD scores and instability signatures between the different sarcoma subtypes. UPS, OS, MFS and ULMS had higher HRD parameter scores compared to MPNST, LMS, DDLPS as well as the fusion driven synovial sarcoma. I am not sure if these differences are **statistically or biologically significant** given large error bar/deviance.
We performed statistics comparing the genomic instability signatures for the different sarcoma entities in Fig.1A-D. Statistical analysis revealed significant differences between:<br>- LOH values in UPS, OS, MFS, LMS, ULMS and MPNST when compared with SS<br>- LOH values in UPS, OS and MFS when compared with DT<br>- LST values in UPS, OS, MFS, LMS and ULMS when compared with SS<br>- LST values in OS when compared with DT<br>- TAI and HRD score values in UPS, OS, MFS, LMS, ULMS, MPNST and DDLPS when compared with SS<br>- TAI and HRD score values in UPS, OS and MFS when compared with DT<br>Detailed statistical analysis can be found in the source data associated with this figure.
2) Figure 2 is incrementally looking at chromosomal gains and losses. Essentially this figure corroborates that HRD high sarcomas had high CIN. Some anecdotal interesting observations are - DDLPS had high Ch. 12 amplification and CIN. A notable finding is that the total number of alterations in HRD genes per sarcoma case shows a bimodal distribution. Based on these data, the **HRD cut-off** value was established to be 32 for authors here for the sarcoma cohort, independent of the subtype. I would like to have understood exactly how the authors used the biomodal distribution data to then compute the HRR specific CIN score ... it was not apparent to me how that was done. Authors then assessed CIN in HRD-high versus HRD-low STS and bone sarcoma cases. Positive controls were serous ovarian cancer and BRAC1/2 aberrant triple negative breast cancer. CRC was used as a negative control. As expected, HRD high STS and bone sarcoma had high aneuploidy and chromosomal duplication events. Authors also interrogated 21 angiosarcoma cases and found some to harbor HRD. It was interesting and not unexpected that the authors found high TMB in sarcoma patients whose tumors/cancer arose in sun-exposed location and thus exhibited a UV-induced mutation pattern.
We incorporated a more detailed explanation of HRD score cut-off calculations in the methods and main text. In summary, we described how we statistically inferred optimal HRD score and LOH cut-off values from HRR-CIN data from the TCGA-SARC cohort: "As molecular alterations in HRR genes in soft tissue sarcoma follow a bimodal distribution (two peaks), we applied a finite mixture model (probabilistic model for representing the presence of subpopulations within an overall population) to separate both subpopulations into HRR-CIN^{high} and HRR-CIN^{low}. The confidence interval from the cut-off was computed by Monte Carlo simulations and resulted in a cut-off value of 37. For each sample, an HRR-CIN status can be defined and associated with an HRD score. In order to infer a cut-off for the HRD score (which is one of the most clinically used biomarkers together with LOH), we plotted a receiver operating characteristic (ROC) curve with HRD score data considering the predicted HRR-CIN status. An ROC curve illustrates the diagnostic ability of a binary classifier as the classification thresholds are varied. ROC curves show the trade-off between sensitivity (x axis: true positive rate) and specificity (y

axis: false positive rate, 1-specificity). While a random classifier gives curves along the diagonal, classifiers that give curves closer to the top-left corner indicate better performance. We next applied the Youden index to select the optimum HRD score cut-off point as it summarizes the ROC curve and shows the maximum potential effectiveness of a biomarker (in this case, the HRD score)".

3) In 2020, Li et al (PMID: 32863940) have previously explored BRCAness and PARP inhibitor sensitivity in 22 STS tumors and compared their findings to analysis of the TCGA data, similar to this study. Their work also included synergy studies with TMZ; however, in that case, so this study is not novel per se. It represents validation of already established data from Li et al publication with new cohort of patients, including angiosarcoma. Importantly, the authors add a new cohort of 282 STS patients where 409 genes were analyzed via targeted panel genome sequencing. Interestingly, some angiosarcoma and Ewing's sarcoma tumors also exhibit high LOH.

We acknowledge the reviewer's comparison of both pieces of work. We were aware of the Li et al. 2020 publication and had cited it accordingly in our manuscript multiple times. As highlighted by the reviewer, we present data from multiple additional sarcoma patients that were unpublished until now: 1) a myxofibrosarcoma cohort of 5 patients in Fig. 3A-F, 2) an angiosarcoma cohort of 21 patients in Fig. 3G-L, and 3) 282 sarcoma patients diagnosed and sequenced at the University Hospital Zurich in Fig. 3M. Moreover, we performed an additional transcriptomic analysis of the TCGA-SARC cohort and showed a distinct gene expression signature in HRD^{high} sarcoma patients (that upregulate HRR pathway genes) and that could validated in our in-house established patient-derived sarcoma cell models, where it predicted PARPi sensitivity.

4) I appreciated the inclusion of **<u>two HRD-low patient derived cell models</u>** from BCOR-rearranged sarcoma and unclassified low-grade sarcoma.

   Functional experiments were performed on the following patient-derived models: 4 MFS, 1 UPS cell line.
   I find the study to be comprehensive, well written with appropriate positive and negative controls included. Although the findings are particularly or entirely novel, they represent an important validation cohort and advancement of understanding profile of HRD in sarcoma and correlated sensitivity to combination therapy employing PARPi and DNA damaging agents. The proof of concept in an index patient case is also compelling for motivating future clinical trials with appropriate measurement metrics built in that will ultimately guide employment of these agents to treat sarcoma in patients selected based on biomarkers or HRD.

We thank the reviewer for this comment. To achieve the same number of HRD^{high} and HRD^{low} sarcoma cell models, we now increased the number of HRD^{low} sarcoma cell models with an extraskeletal myxoid chondrosarcoma, a CIC-DUX fusion sarcoma, solitary fibrous tumour, for which we performed whole-genome sequencing, bulk RNA-sequencing and functional assays (Fig. 5). HRD^{low} sarcoma cell models exhibited few alterations in HRR genes and showed reduced expression of HRR pathway genes compared with HRD^{high} sarcoma cells. In addition, they did not respond to PARP or WEE1 inhibition (Fig. 5).

Referee #3 Minor comments
The acronym CIN appears in Introduction without explanation. It will be beneficial for new reader or broader audience if you briefly elaborated on that.
We briefly described CIN as the high rate of structural and numerical abnormalities in cancer cells compared with normal cells (lines 67-68).

17th Jan 2023

Dear Prof. Pauli,

Thank you for the submission of your revised manuscript to EMBO Molecular Medicine. We have now received the enclosed reports from the two referees who re-reviewed your manuscript. As you will see, they are fully supportive of publication, and I am therefore pleased to inform you that we will be able to accept your manuscript once the following editorial points will be addressed:

1/ Main manuscript text:
- Please address the queries from our data editors in track changes mode in the main manuscript file labelled 'Data edited MS file'. Please use this file for any further modification (in track changes mode, remove the yellow highlights).
- Methods:
o Please rename this section "Materials and Methods"
o Patient samples: please include the full sentence confirming that informed consent was obtained from all subjects and that the experiments conformed to the principles set out in the WMA Declaration of Helsinki and the Department of Health and Human Services Belmont Report.
- The 'Data Availability': Thank you for depositing your bulk mRNA sequencing data. Please note that the datasets must be publicly available before acceptance of the manuscript. All datasets newly generated as part of this manuscript must be deposited in a public repository.
- Acknowledgements: please add the funding information to this section.
- Author contributions: CRediT has replaced the traditional author contributions section because it offers a systematic machine readable author contributions format that allows for more effective research assessment. Please remove the Authors Contributions from the manuscript and use the free text boxes beneath each contributing author's name in our system to add specific details on the author's contribution. More information is available in our guide to authors.
- Please rename "Disclosure and competing interests statement" by "Disclosure and competing interests".
- Please remove "Materials and Correspondence".

2/ Figures and Appendix:
- Exact p values should be provided in the figures or in their legends, or alternatively as a supplemental table in the Appendix.
- Please upload your figures as .eps, .tif, .jpg and remove the figure titles from the figure files.
- The Appendix should be a single pdf file with a Table of Contents and page numbers on the first page.
- Please make sure all figures and figure panels are referenced in the main text, and in chronological order (i.e. Fig 3N is called out before 3M,
Fig 5 panel callouts are not alphabetical, Fig 6K is called out before 6J).

3/ Thank you for providing a nice synopsis picture. Please resize as a PNG/JPEG/TIF file 550 px wide x 300-600 px high, and make sure that the text remains legible.

4/ As part of the EMBO Publications transparent editorial process initiative (see our Editorial at http://embomolmed.embopress.org/content/2/9/329), EMBO Molecular Medicine will publish online a Review Process File (RPF) to accompany accepted manuscripts.
This file will be published in conjunction with your paper and will include the anonymous referee reports, your point-by-point response and all pertinent correspondence relating to the manuscript. Let us know whether you agree with the publication of the RPF and as here, if you want to remove or not any figures from it prior to publication.
Please note that the Authors checklist will be published at the end of the RPF.

I look forward to receiving your revised manuscript.

Yours sincerely,

Lise Roth

Lise Roth, PhD
Senior Editor
EMBO Molecular Medicine

***** Reviewer's comments *****

Referee #1 (Comments on Novelty/Model System for Author):

The manuscript satisfies all criteria for publication in this journal.

Referee #1 (Remarks for Author):

The authors have addressed all the points that were previously raised and the manuscript is now suitable for publication.

Referee #3 (Comments on Novelty/Model System for Author):

The authors added more cell models for functional testing in the revision. Specifically,extraskeletal myxoid chondrosarcoma, a CIC-DUX fusion sarcoma, solitary fibrous tumour.

Referee #3 (Remarks for Author):

The authors satisfactorily addressed comments and suggestions. This is a nicely done paper that can be accepted for publication. As said before, the novelty is not high, but the field needs a definitive large cohort study done well for clinical movement.

The authors addressed the minor editorial issues.

24th Jan 2023

Dear Prof. Pauli,

I am pleased to inform you that your manuscript is accepted for publication and is now being sent to our publisher to be included in the next available issue of EMBO Molecular Medicine.

Please read below for additional IMPORTANT information regarding your article, its publication and the production process.

Congratulations on your interesting work!

With kind regards,

Lise Roth

Lise Roth, Ph.D
Senior Editor
EMBO Molecular Medicine

Follow us on Twitter @EmboMolMed
Sign up for eTOCs at embopress.org/alertsfeeds

EMBO Press Author Checklist

Corresponding Author Name: Chantal Pauli	
Journal Submitted to: EMBO Molecular Medicine	
Manuscript Number: EMM-2022-16863	

USEFUL LINKS FOR COMPLETING THIS FORM
[The EMBO Journal - Author Guidelines]
[EMBO Reports - Author Guidelines]
[Molecular Systems Biology - Author Guidelines]
[EMBO Molecular Medicine - Author Guidelines]

Reporting Checklist for Life Science Articles (updated January 2022)

This checklist is adapted from Materials Design Analysis Reporting (MDAR) Checklist for Authors. MDAR establishes a minimum set of requirements in transparent reporting in the life sciences (see Statement of Task: 10.31222/osf.io/9sm4x). Please follow the journal's guidelines in preparing your manuscript.

Please note that a copy of this checklist will be published alongside your article.

Abridged guidelines for figures

1. Data

The data shown in figures should satisfy the following conditions:

- the data were obtained and processed according to the field's best practice and are presented to reflect the results of the experiments in an accurate and unbiased manner.
- ideally, figure panels should include only measurements that are directly comparable to each other and obtained with the same assay.
- plots include clearly labeled error bars for independent experiments and sample sizes. Unless justified, error bars should not be shown for technical replicates.
- if n<5, the individual data points from each experiment should be plotted. Any statistical test employed should be justified.
- Source Data should be included to report the data underlying figures according to the guidelines set out in the authorship guidelines on Data Presentation.

2. Captions

Each figure caption should contain the following information, for each panel where they are relevant:

- a specification of the experimental system investigated (eg cell line, species name).
- the assay(s) and method(s) used to carry out the reported observations and measurements.
- an explicit mention of the biological and chemical entity(ies) that are being measured.
- an explicit mention of the biological and chemical entity(ies) that are altered/varied/perturbed in a controlled manner.
- the exact sample size (n) for each experimental group/condition, given as a number, not a range;
- a description of the sample collection allowing the reader to understand whether the samples represent technical or biological replicates (including how many animals, litters, cultures, etc.).
- a statement of how many times the experiment shown was independently replicated in the laboratory.
- definitions of statistical methods and measures:
  - common tests, such as t-test (please specify whether paired vs. unpaired), simple χ^2 tests, Wilcoxon and Mann-Whitney tests, can be unambiguously identified by name only, but more complex techniques should be described in the methods section;
  - are tests one-sided or two-sided?
  - are there adjustments for multiple comparisons?
  - exact statistical test results, e.g., P values = x but not P values < x;
  - definition of 'center values' as median or average;
  - definition of error bars as s.d. or s.e.m.

Please complete ALL of the questions below.
Select "Not Applicable" only when the requested information is not relevant for your study.

Materials

Newly Created Materials	Information included in the manuscript?	In which section is the information available? (Reagents and Tools Table, Materials and Methods, Figures, Data Availability Section)
New materials and reagents need to be available; do any restrictions apply?	Not Applicable	

Antibodies	Information included in the manuscript?	In which section is the information available? (Reagents and Tools Table, Materials and Methods, Figures, Data Availability Section)
For **antibodies** provide the following information:<br>- Commercial antibodies: RRID (if possible) or supplier name, catalogue number and or/clone number<br>- Non-commercial: RRID or citation	Yes	Materials and Methods

DNA and RNA sequences	Information included in the manuscript?	In which section is the information available? (Reagents and Tools Table, Materials and Methods, Figures, Data Availability Section)
**Short novel DNA or RNA including primers, probes**: provide the sequences.	Not Applicable	

Cell materials	Information included in the manuscript?	In which section is the information available? (Reagents and Tools Table, Materials and Methods, Figures, Data Availability Section)
**Cell lines:** Provide species information, strain. Provide accession number in repository **OR** supplier name, catalog number, clone number, and/**OR** RRID.	Yes	Materials and Methods
**Primary cultures:** Provide species, strain, sex of origin, genetic modification status.	Yes	Materials and Methods
Report if the cell lines were recently **authenticated** (e.g., by STR profiling) and tested for mycoplasma contamination.	Yes	Materials and Methods

Experimental animals	Information included in the manuscript?	In which section is the information available? (Reagents and Tools Table, Materials and Methods, Figures, Data Availability Section)
**Laboratory animals or Model organisms:** Provide species, strain, sex, age, genetic modification status. Provide accession number in repository **OR** supplier name, catalog number, clone number, **OR** RRID.	Not Applicable	
**Animal observed in or captured from the field:** Provide species, sex, and age where possible.	Not Applicable	
Please detail **housing and husbandry conditions**.	Not Applicable	

Plants and microbes	Information included in the manuscript?	In which section is the information available? (Reagents and Tools Table, Materials and Methods, Figures, Data Availability Section)
**Plants:** provide species and strain, ecotype and cultivar where relevant, unique accession number if available, and source (including location for collected wild specimens).	Not Applicable	
**Microbes:** provide species and strain, unique accession number if available, and source.	Not Applicable	

Human research participants	Information included in the manuscript?	In which section is the information available? (Reagents and Tools Table, Materials and Methods, Figures, Data Availability Section)
If collected and within the bounds of privacy constraints report on age, sex and gender or ethnicity for all study participants.	Yes	Materials and Methods

Core facilities	Information included in the manuscript?	In which section is the information available? (Reagents and Tools Table, Materials and Methods, Figures, Data Availability Section)
If your work benefited from core facilities, was their service mentioned in the acknowledgments section?	Yes	Acknowledgements

Design

Study protocol	Information included in the manuscript?	In which section is the information available? (Reagents and Tools Table, Materials and Methods, Figures, Data Availability Section)
If study protocol has been **pre-registered, provide DOI in the manuscript**. For clinical trials, provide the trial registration number **OR** cite DOI.	Not Applicable	
Report the **clinical trial registration number** (at ClinicalTrials.gov or equivalent), where applicable.	Not Applicable	

Laboratory protocol	Information included in the manuscript?	In which section is the information available? (Reagents and Tools Table, Materials and Methods, Figures, Data Availability Section)
Provide DOI OR other citation details if **external detailed step-by-step protocols** are available.	Not Applicable	

Experimental study design and statistics	Information included in the manuscript?	In which section is the information available? (Reagents and Tools Table, Materials and Methods, Figures, Data Availability Section)
Include a statement about **sample size** estimate even if no statistical methods were used.	Yes	Material and Methods
Were any steps taken to minimize the effects of subjective bias when allocating animals/samples to treatment (e.g. **randomization procedure**)? If yes, have they been described?	Not Applicable	
Include a statement about **blinding** even if no blinding was done.	Yes	Material and Methods
Describe **inclusion/exclusion criteria** if samples or animals were excluded from the analysis. Were the criteria pre-established?<br><br>If sample or data points were omitted from analysis, report if this was due to attrition or intentional exclusion and provide justification.	Yes	Material and Methods
For every figure, are **statistical tests** justified as appropriate? Do the data meet the assumptions of the tests (e.g., normal distribution)? Describe any methods used to assess it. Is there an estimate of variation within each group of data? Is the variance similar between the groups that are being statistically compared?	Yes	Material and Methods

Sample definition and in-laboratory replication	Information included in the manuscript?	In which section is the information available? (Reagents and Tools Table, Materials and Methods, Figures, Data Availability Section)
In the figure legends: state number of times the experiment was **replicated** in laboratory.	Yes	Material and Methods, Figure legends
In the figure legends: define whether data describe **technical or biological replicates**.	Yes	Material and Methods, Figure legends

Ethics

Ethics	Information included in the manuscript?	In which section is the information available? (Reagents and Tools Table, Materials and Methods, Figures, Data Availability Section)
Studies involving **human participants**: State details of **authority granting ethics approval** (IRB or equivalent committee(s), provide reference number for approval.	Yes	Materials and Methods
Studies involving **human participants**: Include a statement confirming that **informed consent** was obtained from all subjects and that the experiments conformed to the principles set out in the WMA Declaration of Helsinki and the Department of Health and Human Services Belmont Report.	Yes	Materials and Methods
Studies involving **human participants:** For publication of **patient photos**, include a statement confirming that consent to publish was obtained.	Not Applicable	
Studies involving experimental **animals**: State details of **authority granting ethics approva**l (IRB or equivalent committee(s), provide reference number for approval. Include a statement of compliance with ethical regulations.	Not Applicable	
Studies involving **specimen and field samples:** State if relevant **permits** obtained, provide details of authority approving study; if none were required, explain why.	Not Applicable	

Dual Use Research of Concern (DURC)	Information included in the manuscript?	In which section is the information available? (Reagents and Tools Table, Materials and Methods, Figures, Data Availability Section)
Could your study fall under dual use research restrictions? Please check biosecurity documents and list of **select agents and toxins** (CDC): https://www.selectagents.gov/sat/list.htm	Not Applicable	
If you used a select agent, is the security level of the lab appropriate and reported in the manuscript?	Not Applicable	
If a study is subject to dual use research of concern regulations, is the name of the **authority granting approval and reference number** for the regulatory approval provided in the manuscript?	Not Applicable	

Reporting

The MDAR framework recommends adoption of discipline-specific guidelines, established and endorsed through community initiatives. Journals have their own policy about requiring specific guidelines and recommendations to complement MDAR.

Adherence to community standards	Information included in the manuscript?	In which section is the information available? (Reagents and Tools Table, Materials and Methods, Figures, Data Availability Section)
State if relevant guidelines or checklists (e.g., **ICMJE, MIBBI, ARRIVE, PRISMA**) have been followed or provided.	Yes	Materials and Methods
For **tumor marker prognostic studies**, we recommend that you follow the **REMARK** reporting guidelines (see link list at top right). See author guidelines, under 'Reporting Guidelines'. Please confirm you have followed these guidelines.	Not Applicable	
For **phase II and III randomized controlled trials**, please refer to the **CONSORT** flow diagram (see link list at top right) and submit the CONSORT checklist (see link list at top right) with your submission. See author guidelines, under 'Reporting Guidelines'. Please confirm you have submitted this list.	Not Applicable	

Data Availability

Data availability	Information included in the manuscript?	In which section is the information available? (Reagents and Tools Table, Materials and Methods, Figures, Data Availability Section)
Have **primary datasets** been deposited according to the journal's guidelines (see 'Data Deposition' section) and the respective accession numbers provided in the Data Availability Section?	Yes	Data availability
Were **human clinical and genomic datasets** deposited in a public access-controlled repository in accordance to ethical obligations to the patients and to the applicable consent agreement?	Not Applicable	
Are **computational models** that are central and integral to a study available without restrictions in a machine-readable form? Were the relevant accession numbers or links provided?	Not Applicable	
If publicly available data were reused, provide the respective **data citations in the reference list**.	Yes	Manuscript main text, References
